# Novel 3D Structure Based Model for Activity Prediction and Design of Antimicrobial Peptides

**DOI:** 10.1038/s41598-018-29566-5

**Published:** 2018-07-25

**Authors:** Shicai Liu, Jingxiao Bao, Xingzhen Lao, Heng Zheng

**Affiliations:** School of Life Science and Technology, China Pharmaceutical University, Nanjing, 210009 China

## Abstract

The emergence and worldwide spread of multi-drug resistant bacteria makes an urgent challenge for the development of novel antibacterial agents. A perspective weapon to fight against severe infections caused by drug-resistant microorganisms is antimicrobial peptides (AMPs). AMPs are a diverse class of naturally occurring molecules that are produced as a first line of defense by all multi-cellular organisms. Limited by the number of experimental determinate 3D structure, most of the prediction or classification methods of AMPs were based on 2D descriptors, including sequence, amino acid composition, peptide net charge, hydrophobicity, amphiphilic, etc. Due to the rapid development of structural simulation methods, predicted models of proteins (or peptides) have been successfully applied in structure based drug design, for example as targets of virtual ligand screening. Here, we establish the activity prediction model based on the predicted 3D structure of AMPs molecule. To our knowledge, it is the first report of prediction method based on 3D descriptors of AMPs. Novel AMPs were designed by using the model, and their antibacterial effect was measured by *in vitro* experiments.

## Introduction

In 2014, the WHO’s (World Health Organization) report about global surveillance of antimicrobial resistance reveals that antibiotic resistance is no longer a prediction for the future^1^. It is happening now, across the world. With the emergence of more and more multi-drug resistant bacteria, the development of new antibacterial drugs turns into an urgent challenge^2^. A perspective weapon to fight against severe infections caused by drug-resistant microorganisms is antimicrobial peptides (AMPs)^3–7^. AMPs are a diverse class of naturally occurring molecules that are produced as a first line of defense by all multi-cellular organisms^8^. These peptides can have broad activity to kill bacteria, fungi, yeasts, viruses and even cancer cells. In addition, AMPs have been found to display immunomodulatory functions such as wound healing, chemotactic, angiogenic^7,9^, which make them even more attractive templates for the new-generation antibiotics.

There are more than 2,500 AMPs found in nature^10^, such as single-celled organisms, plants, insects, animals. Most of the AMPs information is included in the DRAMP database^11^ established by our laboratory. Although AMPs have become as promising candidates to traditional antibiotics for treatment of bacterial diseases, many potential problems should be solved before they can be put in clinic and commerce, including instable and easy to be digested by enzyme *in vivo*, relatively low activity comparing with antibiotics, toxicity against eukaryotic cells, high production costs^12^. There still needs much effort on designing novel AMPs to overcome these limitations. In recent years, machine learning has been applied in AMPs analysis, which may become useful tool to speed up the classification, prediction and design of AMPs^13^. By using the database resources, the AMPs information was extracted to establish the activity prediction model. At present, most of the activity prediction models are established based on the primary structure of AMPs^14–18^, the amino acid composition, peptide net charge, hydrophobicity, amphiphilic, helix and other structural parameters are all critical for AMPs’ activity. Changing any of these parameters can lead to AMPs’ activity reduced or even lost. There is a strong correlation between all parameters, and it is not comprehensive enough to predict its antibacterial activity by a given amino acid sequence of AMPs. Feature extraction of AMPs is an important step in data analysis and machine learning. Even the most sophisticated algorithms would perform poorly if inappropriate features are used, while simple methods can potentially perform well when they are fed with the appropriate features. Therefore, in this study, we will establish the activity prediction model based on 3D structure of the AMPs molecule. However, the 3D structure of most AMPs is unknown, only a small part of the 3D structure of the AMPs is determined. Only 5.5% of the AMPs’ 3D structure were determined in General dataset of DRAMP database^11^. A variety of methods have been developed for the prediction of proteins’ 3D structure in the field of computational biology, including homology modeling^19^, folding recognition^20^, and *ab initio* calculations^21–23^. The first two methods are based on the known protein structure as a template to generate the structure by sequence alignment. With the advancement of molecular dynamics simulation technology, the modeled structure is generally considered to be reasonable and credible after a period of molecular dynamics simulation^24^. Therefore, we plan to predict the 3D structure of AMPs by homology modeling and molecular dynamics simulation.

In this study, molecular dynamics simulations for 84 peptides have been performed, and we establish the activity prediction model based on the predicted 3D structure of the AMPs molecule. To our knowledge, it is the first report of prediction method based on 3D descriptors of AMPs. Novel AMPs were designed by using the model, and their antibacterial effect was measured by *in vitro* experiments.

## Methods

### Molecular dynamics simulations

The starting 3D structure model of AMPs was generated based on homology modeling using MOE^25^. The GB/VI^26^ was used as the scoring standard of the model. Other parameters were set as the default values. The homology modeling templates of AMPs are in Supplemental Table S1.

The starting 3D structure models were then optimized with molecular dynamics (MD) simulations. MD simulations of AMPs were performed in AMBER package^27^ using the FF14SB force field^28^. The starting 3D structure model was first solvated with a truncated octahedron box of TIP3P water molecules^29^ that extended 10 Å from the atoms and Na^+^ and Cl^−^ neutralizing counterions. Prior to the start of the production simulation, 5000 steps of energy minimization were performed using steepest descent and conjugate gradient method, respectively. Long range electrostatic interactions were addressed by particle mesh Ewald summation, with a real space cutoff of 1.0 nm.

Production runs were conducted at 300 K for 100 ns with data collected every 100 ps. For all simulations, a time step of 2 fs was employed. A Langevin thermostat was used to maintain temperature and a Monte carlo at 1 atm was used to control pressure.

### Datasets

**Figure 1:**
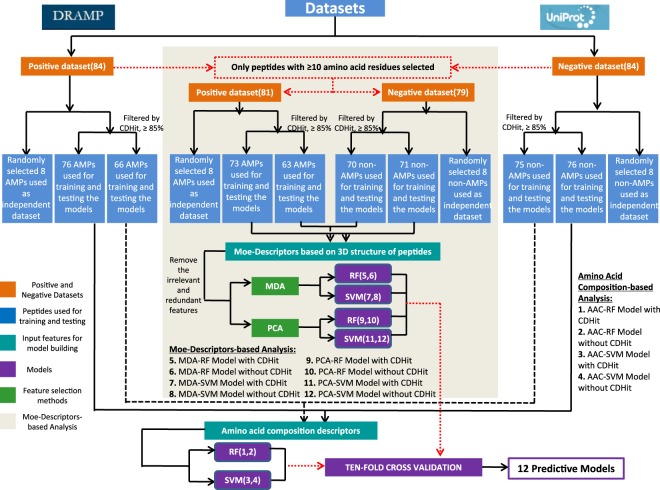
Flowchart depicting the overall approach implemented as the establishment of activity prediction model. The flowchart gives an overview of the steps followed in building the predictive models.

We have extracted 84 experimentally validated anti-listerial peptides from DRAMP databases^11^. All these peptides were unique and considered as positive examples (Supplementary Table S1). Since there are very few experimentally proved non-antilisterial peptides, we derived 84 random peptides from SwissProt^30^ proteins with the keywords, “not antimicrobial activity”, “not antibactreial activity”, “not antilisiterial activity”, “a length range of 5–70 amino acids” and “have 3D structure”. In this study, we assign these random peptides as non-antilisterial peptides (negative examples, Supplementary Table S2), though it is possible that some of these random peptides have antimicrobial properties. After obtaining the positive dataset and negative dataset, the training set and the testing set were screened with CD-Hit^31^, with sequence identity cut-off of 85% in order to remove sequence redundancy in the set. Then, the screened and unscreened data sets were used to establish the prediction model, respectively (Fig. 1).

### Feature extraction

Both local and global descriptors were used to characterize peptide structures. The amino acid descriptors amino acid composition (AAC) was employed as local characterization to parameterize peptides. From some literature we know that the AAC is the most important factor for peptide classification and design, so it may be a good choice. For AAC calculation only 20 naturally amino acids are considered, and it has been successfully used for many protein classification problems^32^. AAC can be calculated using the formula below:

$${\rm{AAC}}({\rm{i}})=\frac{{\rm{Total}}\,{\rm{number}}\,\mathrm{of}\,\,{\rm{amino}}\,{\rm{acid}}({\rm{i}})}{{\rm{Total}}\,{\rm{number}}\,{\rm{of}}\,{\rm{all}}\,{\rm{possible}}\,{\rm{amino}}\,{\rm{acids}}}$$

Global structure characterization named MOE-Descriptors was carried out using MOE (https://www.chemcomp.com/)^25^ based on 3D structure of AMPs: The peptide structures were converted to three classes of molecular descriptors as 2D Molecular Descriptors, Protein Property Descriptors and 3D Molecular Descriptors by using MOE program. The 3D Molecular Descriptors include Potential Energy Descriptors, MOPAC Descriptors, Conformation Dependent Charge Descriptors, Surface Area, Volume and Shape Descriptors^33–36^. For example, the energy descriptors use the MOE potential energy model to calculate energetic quantities (in kcal/mol) from stored 3D conformations. For detailed information about the 3D descriptors, see the MOE manual (http://www.chemcomp.com/MOE-Cheminformatics_and_QSAR.htm#MolecularDescriptors). Some of these features may not be relevant to the prediction of AMPs and they could be also redundant with each other. So, we performed two feature selection methods, the mean decrease in accuracy (MDA) and principal component analysis (PCA), to remove or merge the irrelevant and redundant features, which was calculated using the randomForest package and SciViews package in R (http://cran.r-project.org//), respectively. MDA represents the average decrease of classification accuracy on the OOB samples when the values of a particular feature are randomly permuted. Thus the permutation based MDA can be utilized to evaluate the contribution of each feature to the classification. After excluding collinear and irrelevant descriptors, 90 molecular descriptors selected by MDA and 26 principal components derived by PCA were used for further analysis.

### Regression modeling

Two machine learning methods, support vector machine (SVM)^37^ and random forest (RF)^38^, were employed to conduct regression modeling of the multivariate correlation between the peptide structural parameters and antibacterial activity. SVM was implemented by using e1071 package in R (http://cran.r-project.org//). SVM is a classification algorithm based on statistical learning theory, which aims at the structural risk minimization rather than the traditional empirical risk minimization and is especially suitable for small-sample, high-dimensional and strong collinear problems. RF was implemented using random Forest package in R. RF uses an ensemble of unpruned decision trees, each grown using a bootstrap sample of the training data, and randomly selected subsets of predictor variables as candidates for splitting tree nodes, which is to maintain the “strength” of the trees while reducing their correlation with each other.

### Evaluating performance

Once the models were ready, their performance was tested in terms of the sensitivity, specificity, accuracy, and Mathew’s Correlation Coefficient (MCC). They can be calculated using the formula below:

$${\rm{Sensitivity}}=\frac{{\rm{TP}}}{{\rm{TP}}+{\rm{FN}}}$$

$${\rm{Specificity}}=\frac{{\rm{TN}}}{{\rm{TN}}+{\rm{FP}}}$$

$${\rm{Accuracy}}=\frac{{\rm{TP}}+{\rm{TN}}}{{\rm{TP}}+{\rm{FP}}+{\rm{TN}}+{\rm{FN}}}$$

$${\rm{MCC}}=\frac{{\rm{TP}}\times {\rm{TN}}-{\rm{FP}}\times {\rm{FN}}}{\sqrt{({\rm{TP}}+{\rm{FP}})({\rm{TP}}+{\rm{FN}})({\rm{TN}}+{\rm{FP}})({\rm{TN}}+{\rm{FN}})}}$$

where TP, FP, TN and FN stand for the number of true positives, false positives, true negatives and false negatives, respectively.

The performance of the models was evaluated by employing a ten-fold cross-validation technique. The whole dataset was divided into ten sets such that in each round, nine sets were used for training and one was set aside for testing. Repeated ten times, this ensured that each set was used once for testing the model that was trained on the remaining nine.

In order to evaluate the performance of our models, we have created an independent dataset of 8 AMPs randomly selected from the final 84 AMPs and 8 non-AMPs randomly selected from the final 84 non-AMPs, which have not been included in the training, feature selection and parameters optimization of the model.

### *In silico* optimization of AMPs

**Figure 2:**
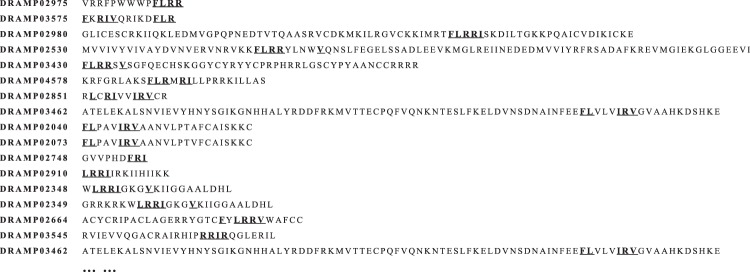
Sequence alignment of the natural AMPs from DRAMP. Underlining represents consensus sequence amino acids.

**Figure 3:**
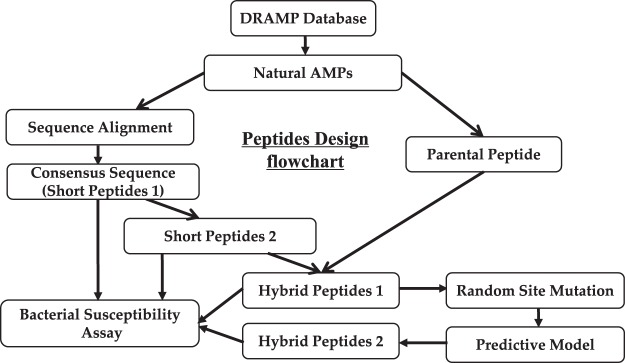
The flowchart gives an overview of the steps followed in designing novel bioactive peptides.

By using database resources, natural AMPs from DRAMP were used for sequence alignment, and a 7-amino-acid consensus sequence (**short peptides 1**, FLRRIRV-NH_2_) was apparent in some peptides (Fig. 2), and was selected as seed peptide. The second position of most AMPs is tryptophan^39^, contributing to the anchoring of AMPs on the cell membrane. The leucine at position 2 of the consensus sequence was transformed into tryptophan, resulting in **short peptides 2** (FWRRIRV-NH_2_). We argued that smaller peptides would be less expensive to produce and that a reduction in the number of amino acids would allow a more comprehensive understanding of the amino acid sequence responsible for antimicrobial activity. Therefore, we screened the sequence from DRAMP, with sequence length less than fifteen, complete sequence information and anti-listeria activity. Finally, we get the parental peptide DRAMP00228^11,40^ (TPVVNPPFLQQT-NH_2_, DRAMP ID began with “DRAMP” and five-digit number followed). We link short peptides and parental peptide, and random single-point was imposed on the hybrid peptides, resulting in random mutant. The mutation introduces only natural amino acids to the peptide. After mutation the antimicrobial activity of the mutant was predicted by using the predictive models, and the activity test was carried out (Fig. 3**)**.

### Bacterial susceptibility assay

All peptides used in this study were synthesized by ChinaPeptides (ChinaPeptides Co., Ltd) using 9-fluorenylmethoxy carbonyl (Fmoc) chemistry and purified to a purity of >95% using high-performance liquid chromatography (HPLC). Peptide mass was confirmed by mass spectrometry.

The experimentally determined strains are as follows: *Listeria monocytogenes* (ATCC 19115), *Staphylococcus aureus* (CMCC(B)26003), *Bacillus subtilis* (CMCC(B)63501), *Escherichia coli* (CMCC(B) 44102), *Pseudomonas aeruginosa* (CMCC(B)10104), *Enterococcus faecalis* (clinical strains) from China Pharmaceutical University Microbiology Laboratory.

Minimal inhibitory concentration(MIC) of peptides were determined using broth microdilution method. Two-fold serial dilutions of eight peptides were prepared from 1024.0 to 1.0 μg/ml in 96-well microtiter plate (100.0 μl of each well). Then peptide dilutions were mixed with LB broth and bacterial culture (100.0 μl) containing 2.0 × 10^5^ CFU/ml. Final peptide concentrations ranged from 0.5 to 512.0 μg/ml. The final bacterial concentration was approximately 1.0 × 10^5^ CFU/ml. Positive controls were incubated with Cefuroxime instead of peptide, at concentrations from 0.5 to 512.0 μg/ml. Negative and blank controls were incubated, respectively, with sterile deionized water or only LB broth. Microtiter plates were incubated at 37 °C for 24 h under normal atmospheric conditions. OD_600_ was measured using a microplate spectrophotometer (Multiskan GO, Thermo Scientific, USA). MIC was recorded as the endpoint where no difference of OD_600_ could be detected with respect to the blank LB broth^41^. MIC assays were performed three times for all strains.

## Results

### Molecular dynamics simulations

**Figure 4:**
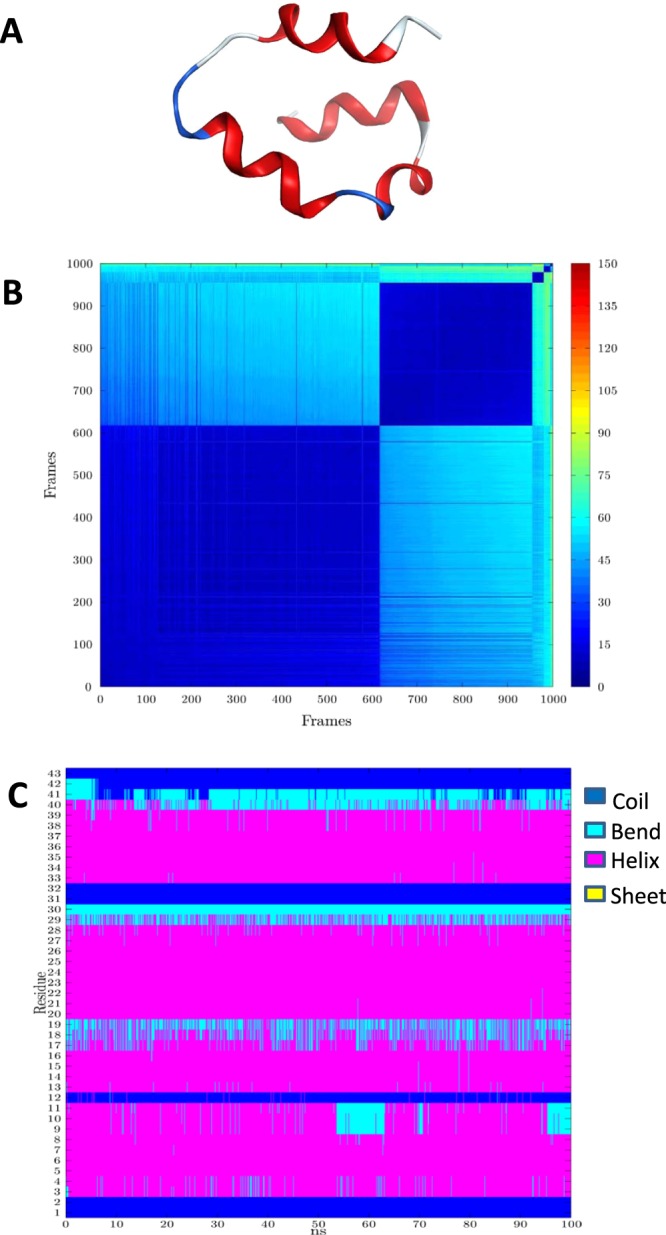
The results of MD simulation. (**A**) Representative Structure produced by MD simulation for DRAMP18261. (**B**) Heat map plot produced by MD simulation for DRAMP18261. (**C**) Secondary Structure assumed by each amino acid throughout the MD simulation for DRAMP18261.

MD simulations for 84 peptides (the positive dataset, Fig. 1) have been performed. In these 84 peptides there were only five peptides have experimental determined structures. However, we still carried out homology modeling and MD simulation for the five peptides, to valid the structure prediction method by comparison of the predict model with the known crystal structure. For example, the crystal structure (PDB ID: 2m60) of DRAMP18261 is compared with the representative structure (Fig. 4) obtained after the structure simulation to obtain the RMSD value of 1.968 Å, indicating that the simulation result is feasible.

A simplified similarity measure, C^α^ torsion angle, was used to analyze and present the MD simulation results. The C^α^ torsion angle is defined as the non-bonded torsion angle arising from four consecutive C^α^ atoms along the chain of the peptide. For each frame of the MD simulation, an array of C^α^ torsion angles for each of the amino acids in the peptide was created^42–44^. The “representative structure” was identified as the simulation frame whose array has the smallest mean Root Mean Square Deviation to all the other frames in the MD simulation, and a PDB file for this frame was generated (Fig. 4A). In addition, the C^α^ torsion angle arrays were used to create a heat map plot, showing the simulation frames groupings with similar structures and suggesting the number of different structures’ types arising during the MD simulation. The heat map was produced by first re-ordering all of the simulation frames according to increasing distance of their corresponding C^α^ torsion angle array. The C^α^ torsion angle distance is calculated between all pairs of frames in the MD simulation trajectory. The heat map was then constructed with each axis corresponding to all the simulation frames ordered as described above. Each element of the heat map represents the color-coded difference between the arrays for the two corresponding frames (Fig. 4B), Note that the heat map is symmetrical above and below the diagonal, the latter corresponding to the comparison between each simulation frame and itself. The propensity of each amino acid position along the peptide to assume a secondary structure type (helix, sheet, bend or coil) over the course of the MD simulation was determined using the program “AmberTools”^27^, and the secondary structure type for each amino acid versus simulation frame have been plotted (Fig. 4C). The results of the MD simulation of 84 AMPs are in Supplementary Figure S3. The resulting “representative structures” were used to establish the activity prediction models as per the procedure illustrated in the methods section. The peptides having crystal structure are using crystal structure to extracting feature.

### Machine learning regression modeling

The statistics of twelve models were summarized in Tables 1 and 2. The accuracies of the **AAC-RF with CDHit (1)** and **AAC-SVM with CDHit (3)** based models were 89.26% and 85.71%, with MCC values of 0.79 and 0.72 respectively, while the **AAC-RF without CDHit (2)** and **AAC-SVM without CDHit (4)** based models performed with accuracies of 80.00% and 86.67%, the corresponding MCC values being 0.60 and 0.74 respectively. To get best prediction results, only the **AAC-RF with CDHit (1)** based models with accuracy 89.26% and MCC 0.79 were selected.

**Table 1:** Performance of the models based on amino acid composition of the peptides on training datasets.

Classifier	Sensitivity	Specificity	Accuracy	MCC
AAC-RF with CDHit(**1**)	72.73	100.00	89.26	0.79
AAC-RF without CDHit(**2**)	71.43	87.50	80.00	0.60
AAC-SVM with CDHit(**3**)	63.64	100.00	85.71	0.72
AAC-SVM without CDHit(**4**)	78.57	93.75	86.67	0.74

AAC: amino acid composition; RF: Random Forest algorithm; SVM: support vector machine algorithm; with CDHit: CDHit-screened datasets; without CDHit: CDHit-unscreened datasets.

**Table 2:** Performance of the models based on MOE-Descriptors of the peptides’ 3D structure on training datasets.

Classifier	Sensitivity	Specificity	Accuracy	MCC
MDA-RF with CDHit(**5**)	100.00	100.00	100.00	1.00
MDA-RF without CDHit(**6**)	100.00	100.00	100.00	1.00
MDA-SVM with CDHit(**7**)	90.00	94.12	92.59	0.84
MDA-SVM without CDHit(**8**)	92.86	86.67	89.66	0.80
PCA-RF with CDHit(**9**)	80.00	88.24	85.19	0.68
PCA-RF without CDHit(**10**)	78.57	86.67	82.76	0.66
PCA-SVM with CDHit(**11**)	90.00	94.12	92.59	0.84
PCA-SVM without CDHit(**12**)	100.00	66.67	82.76	0.70

MDA: mean decrease in accuracy; PCA: principal component analysis; RF: Random Forest algorithm; SVM: support vector machine algorithm; with CDHit: CDHit-screened datasets; without CDHit: CDHit-unscreened datasets.

The MOE-Descriptors of the peptides’ 3D structure to be used as input features were selected for building the RF and SVM-based models (Fig. 1). Performances of MOE Descriptors-based models were summarized in Table 2. The models were evaluated using a ten-fold cross validation technique as per the procedure illustrated in the Methods section. As might be expected, overall, MOE-Descriptors of the peptides’ 3D structure performed much well as compared to amino acid composition descriptor in sensitivity, specificity, accuracy, and MCC (Tables 1, 2). In addition, the models of dataset screened with CD-Hit performed much well as compared to the models based on unscreened dataset. In the models based on MOE-Descriptors, although scoring function results of the **MDA-RF with CDHit (5)** and **MDA-RF without CDHit (6)** based model were all 1.00 (Table 2), their independent dataset results did not perform well (Table 3), which is occuring overfitting phenomenon. The **MDA-SVM with CDHit (7)** (accuracy of 92.59% with sensitivity, specificity and MCC of 90.00, 94.12 and 0.84, respectively) and **PCA-SVM with CDHit (11)** (accuracy of 92.59% with sensitivity, specificity and MCC of 90.00, 94.12 and 0.84, respectively) based model exhibit the similar profile in sensitivity, specificity, accuracy and MCC, although some difference between them on independent dataset results can be observed. Compare to the **MDA-SVM with CDHit (7)**, the **PCA-SVM with CDHit (11)** based model has the comparable fitting ability on training set but worse predictive power on independent dataset. In all the models **MDA-SVM with CDHit (7)** seems to have the best performance in internal stability and external predictability with accuracy 92.59%, MCC 0.84 (on training set), and accuracy 100.00%, MCC 1.00 (on independent dataset), suggesting that the combination of SVM and MOE descriptors processed with MDA on CDHit-screened datasets is a good choice that exhibits high internal stability and strong external predictive power.

**Table 3:** Performance of the models on independent datasets.

Classifier	Sensitivity	Specificity	Accuracy	MCC
AAC-RF with CDHit(**1**)	71.43	87.50	80.00	0.60
AAC-RF without CDHit(**2**)	87.50	75.00	81.25	0.63
AAC-SVM with CDHit(**3**)	87.51	75.00	80.00	0.61
AAC-SVM without CDHit(**4**)	87.50	75.00	81.25	0.63
MDA-RF with CDHit(**5**)	100.00	87.50	93.33	0.88
MDA-RF without CDHit(**6**)	100.00	87.50	93.75	0.88
MDA-SVM with CDHit(**7**)	100.00	100.00	100.00	1.00
MDA-SVM without CDHit(**8**)	87.50	100.00	93.75	0.88
PCA-RF with CDHit(**9**)	100.00	87.50	93.33	0.88
PCA-RF without CDHit(**10**)	87.50	87.50	87.50	0.75
PCA-SVM with CDHit(**11**)	87.51	100.00	93.33	0.87
PCA-SVM without CDHit(**12**)	87.50	100.00	93.75	0.88

In order to validate our *in silico* methods, performances of our models were evaluated on independent dataset. Positive and negative independent datasets of 16 peptides were used to judge the predictive capacity of the twelve models (Fig. 1). All these models performed reasonably good as shown in Table 3, demonstrating that these models are useful or effective in real life. The **MDA-SVM with CDHit (7)** performed with the highest accuracy (with accuracy, sensitivity, specificity and MCC of 100.00%, 100.00%, 100.00% and 1.00, respectively) among all these models. Performances on both the training and independent datasets were considered to select the best models for the design of novel AMPs.

### *In silico* optimization of AMPs

As per the procedure illustrated in the methods section (Fig. 3), after obtaining the short peptide 1(FLRRIRV-NH_2_) and short peptide 2 (FWRRIRV-NH_2_), the short peptides are bound to the parental peptide DRAMP00228 (TPVVNPPFLQQT-NH_2_), respectively, resulting the hybrid peptide 1 (FLRRIRV-TPVVNPPFLQQT-NH_2_ and FWRRIRV-TPVVNPPFLQQT-NH_2_). Random mutational point of the hybrid peptide 1 is performed, resulting in nearly 1000 new peptides. Due to these new peptides do not have 3D structure, a preliminary prediction based on amino acid composition (**AAC-RF with CDHit (1)**) was used, and approximately 350 of these new peptides are predicted to be active. Then, we randomly chose 30 peptides from the preliminary selection result for 3D structure simulation. Then **MDA-SVM with CDHit (7)** based model was used to predict the peptides’ activity after getting 3D structure. Finally, we selected five peptides (including the hybrid peptide 1) from the prediction results for experimental validation. Although the predictive models are established based on anti-listeria activity of AMPs, several of the strains were tested in the case of experimental validation.

**Figure 5:**
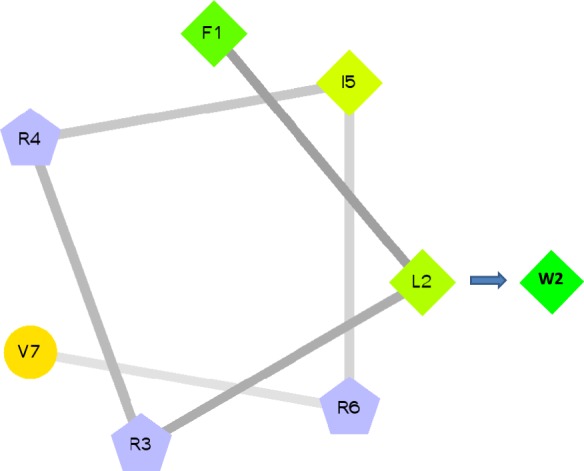
Helical wheel projection diagrams of the short peptides. Arrows indicate the substituted-amino acids. Hydrophilic residues are represented in circles, hydrophobic residues in diamonds, positive charged residues in pentagons. The most hydrophobic residue is green, and the amount of green is decreasing proportionally to the hydrophobicity.

The results were summarized in Table 4. Consequently, the short peptides FLRRIRV-NH_2_ showed an ability to inhibit *Listeria monocytogenes* (ATCC 19115) and *Staphylococcus aureus* (CMCC(B)26003) with MIC 128 μg/ml. Moreover, FWRRIRV-NH_2_ displayed higher antibacterial activity across *Listeria monocytogenes* (ATCC 19115), *Staphylococcus aureus* (CMCC(B)26003) and *Bacillus subtilis* (CMCC(B)63501) with MIC 32 μg/ml, 64 μg/ml, 128 μg/ml, respectively. The leucine at position 2 of FLRRIRV-NH_2_ transformed into tryptophan makes it significantly more active. The short peptides adopt an amphipathic conformation on Helical wheel projection diagrams (Fig. 5). We argue that amino acid change at position 2 of FLRRIRV-NH_2_ increased amphiphilicity. Tryptophan is a hydrophobic amino acid containing a benzene ring, which can effectively promote the anchoring of AMPs on the cell membrane, resulting in activity of the peptide increased. The parent peptide TPVVNPPFLQQT-NH_2_ has only antilisteria activity with MIC 512 μg/ml.

**Table 4:** Antibacterial activity of short peptides, parental peptide, and designed peptides (MIC μg/ml).

	*Listeria monocytogenes*	*Staphylococcus aureus*	*Bacillus subtilis*	*Escherichia coli*	*Pseudomonas aeruginosa*	*Enterococcus faecalis*
**Short Peptides**						
FLRRIRV-NH_2_	128	128	—	—	—	—
FWRRIRV-NH_2_	32	64	128	—	—	—
**Parental Peptide**						
TPVVNPPFLQQT-NH_2_	512	—	—	—	—	—
**Designed Peptides**						
FLRRIRVTPVVNPPFLQQT-NH_2_	—	—	—	—	—	—
FLRRIRVTPWVNPPFLQQT-NH_2_	128	256	—	—	—	—
FWRRIRVTPVVNPPFLQQT-NH_2_	256	256	—	—	—	—
FWRRIRVTPVVNPWFLQQT-NH_2_	32	32	64	256	256	—
FWRRIRVTPWVNPPFLQQT-NH_2_	64	64	256	—	—	—
**Positive Control**						
Cefuroxime	4	4	≤0.5	8	—	16

“—” indicates that the peptide is inactive at 512 μg/ml.

In the five designed peptides, except for FLRRIRVTPVVNPPFLQQT-NH_2_ with the predicted result, no anti-listeria activity, and the other four predicted results are all active, which is suggesting that the predicted results are consistent with the experimental verification results. FLRRIRVTPWVNPPFLQQT-NH_2_ showed a ability to inhibit *Listeria monocytogenes* (ATCC 19115) and *Staphylococcus aureus* (CMCC(B)26003) with MIC 128 μg/ml, 256 μg/ml, respectively. FWRRIRVTPVVNPPFLQQT-NH_2_ showed a ability to inhibit *Listeria monocytogenes* (ATCC 19115) and *Staphylococcus aureus* (CMCC(B)26003) with MIC 256 μg/ml. FWRRIRVTPWVNPPFLQQT-NH_2_ showed a ability to inhibit *Listeria monocytogenes* (ATCC 19115), *Staphylococcus aureus* (CMCC(B)26003) and Bacillus subtilis (CMCC(B)63501) with MIC 64 μg/ml, 64 μg/ml, 256 μg/ml, respectively. The designed peptide FWRRIRVTPVVNPWFLQQT-NH_2_ showed a marked ability to inhibit *Listeria monocytogenes* (ATCC 19115), *Staphylococcus aureus* (CMCC(B)26003), *Bacillus subtilis* (CMCC(B)63501), *Escherichia coli* (CMCC(B) 44102) and *Pseudomonas aeruginosa* (CMCC(B)10104) compared with the parental peptides, with MIC 32 μg/ml, 32 μg/ml, 64 μg/ml, 256 μg/ml, 256 μg/ml, respectively. These assays confirmed that these designed peptides displayed approximately 2-16-fold higher antibacterial activity across *Listeria monocytogenes* (ATCC 19115) in comparison to their parent peptide TPVVNPPFLQQT-NH_2_ and showed an extended antibacterial spectrum.

## Discussion and Conclusion

**Figure 6:**
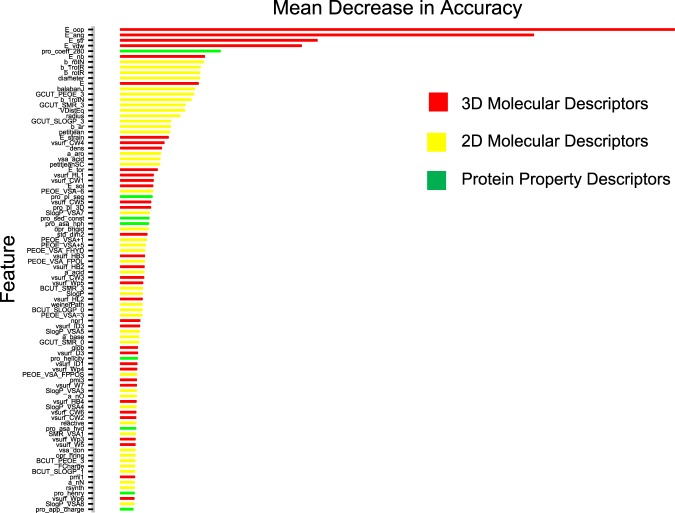
The top 90 features after feature selection based MDA on MOE-Descriptors.

An integrated in silico–*in vitro* discovery of bioactive peptides was described to perform computer-aided rational design of antimicrobial peptides. In the procedure, regression models were built based on peptides’ 3D structure and validated rigorously. The performance of **MDA-SVM with CDHit (7)** based model was measured with an accuracy of 92.59% and a MCC of 0.84 on the training and testing dataset. Additionally, **MDA-SVM with CDHit (7)** was evaluated using an independent dataset resulting in an accuracy of 100.00%, which were then employed to direct *in silico* optimization of AMPs, attempting to obtain a new AMPs population with improved antimicrobial potency. During the process of feature selection based MDA, we selected the top 90 features in MOE-Descriptors to constitute model which achieved the best result. The top 90 features were shown in Fig. 6. From Fig. 6, we drew a conclusion that 3D Molecular features were more important for the modeling, mainly including Potential Energy Descriptors, Surface Area, Volume and Shape Descriptors. In the top 90 features, the first 4 all belong to Potential Energy Descriptors, and the abscissa value of the four features is much larger than the others, indicating that their importance is much higher than other features. 3D structure descriptors are closer to reality, and better reflect the essence of the peptides drug. At present, most of the activity prediction models are established based on the primary structure of AMPs, such as MLAMP^14^, the method of Gupta *et al*.^15^, iACP^16^, CPPpred^17^, iAMPpred^18^. In MLAMP^14^, a two-level multi-class predictor was developed for identification of AMPs, based on amino acids frequency and biochemical properties. In the method of Gupta *et al*.^15^, random forest(RF) and support vector machine(SVM) supervised learning techniques were employed for prediction of AMPs, based on compositional features and sequence motifs features of peptides. The iACP tool was developed for predicting the propensity of a peptide sequence as anticancer peptides by using SVM machine learning techniques^16^. In CPPpred^17^, the model was developed for prediction of cell penetrating peptides. In iAMPpred^18^, Meher *et al*. have developed a machine learning based computational approach for improved recognition of AMPs. The above mentioned methods have their own advantages in generating knowledge for the prediction of AMPs. But all the above method based on 1D or 2D descriptors of peptide, such as amino acid component, physiochemical property etc. The 3D structural properties of the peptide were not included in above mentioned method. In this study, we probed a novel prediction method based on predicted 3D structure of the AMPs molecule. Although currently the method is limited by the time-consuming step of structure prediction step, and is difficult to directly apply in large amount screening, it showed a potential powerful complement to traditional 1D or 2D methods. And with the rapid progress of Structural Proteomics and computing capabilities, we can expect more and more 3D structure based method be developed and applied in peptide activity prediction.

**Figure 7:**
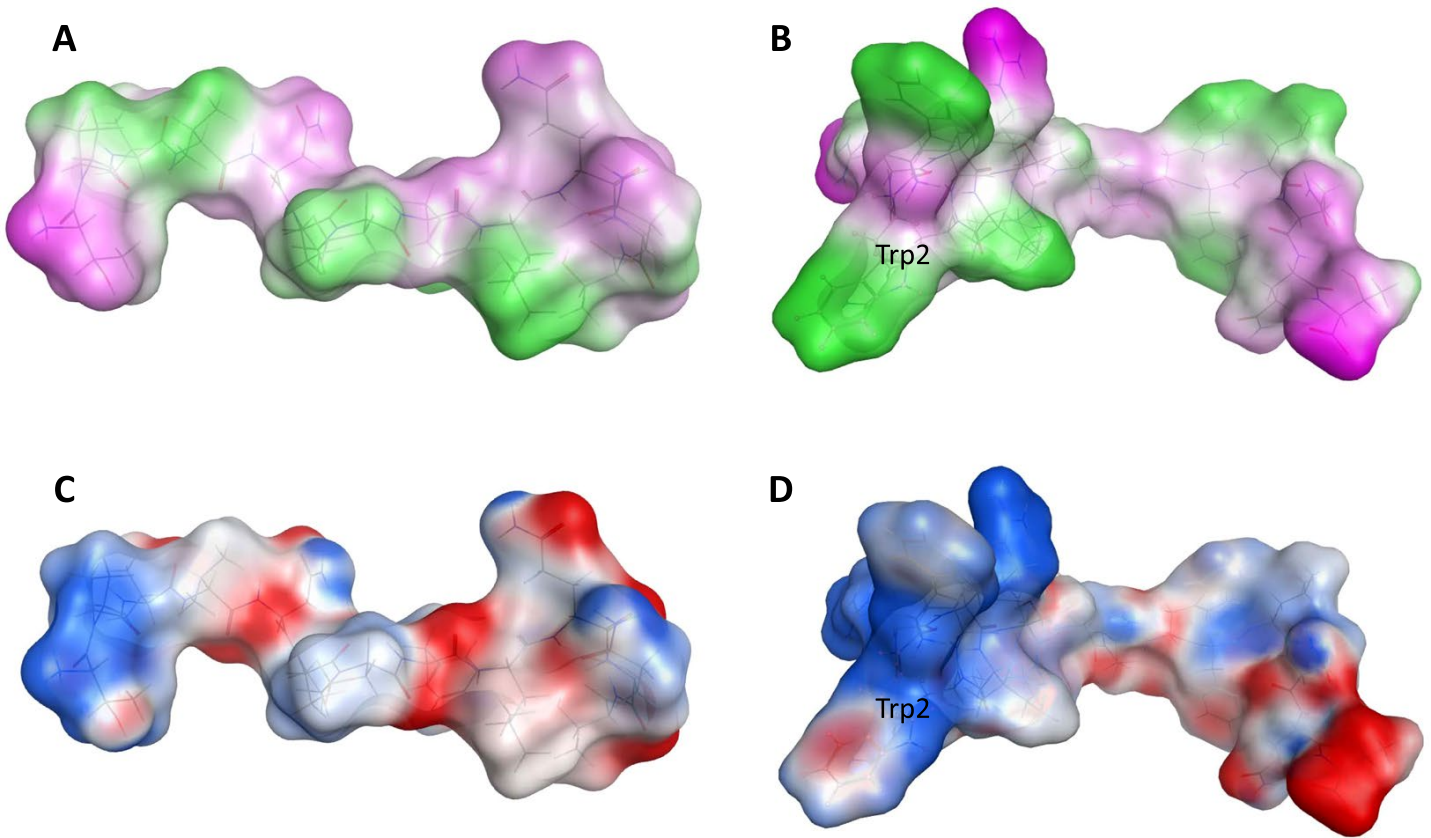
(**A**,**B**) Show the amphiphilic distribution of the parent peptide TPVVNPPFLQQT-NH_2_ (**A**) and the designed peptide FWRRIRVTPVVNPWFLQQT-NH_2_ (**B**), respectively: Pink indicates hydrophilicity, white indicates neutral, and green indicates hydrophobic. (**C**,**D**) Show charge distribution of the parent peptide TPVVNPPFLQQT-NH_2_ (**C**) and the designed peptide FWRRIRVTPVVNPWFLQQT-NH_2_ (**D**), respectively: Blue indicates positive charge, white indicates no charge, red indicates negative charge.

Five AMPs were successfully designed and synthesized, and their antibacterial activity was tested against six bacteria. Consequently, the results predicted by regression model are consistent with the experimental verification results. The designed peptide FWRRIRVTPVVNPWFLQQT-NH_2_ exhibited the highest activity in all the tested candidates, showing a marked ability and an extended antibacterial spectrum to inhibit *Listeria monocytogenes* (ATCC 19115), *Staphylococcus aureus* (CMCC(B)26003), *Bacillus subtilis* (CMCC(B)63501), *Escherichia coli* (CMCC(B) 44102) and *Pseudomonas aeruginosa* (CMCC(B)10104) compared with the parental peptides, with MIC 32 μg/ml, 32 μg/ml, 64 μg/ml, 256 μg/ml, 256 μg/ml, respectively. In our opinion, the broadening of the antibacterial spectrum of these designed peptides may be due to the influence of amino acid at position 2. The activity is increased and the antibacterial spectrum is expanded after the hydrophobic amino acid L replaced by the strongly hydrophobic amino acid W. Lv *et al*.^45^ found that the existence of the high hydrophobic amino acid tryptophan of GI24(GRFRRLRKKTRKRLKKIGKVLKWI-NH_2_) plays a vital role in its antibacterial activity through the single site-mutation study. To investigate the contribution of W at position 23 of GI24 on the antimicrobial activity, a series of W-substituted mutants were developed by substituting W with A, K, and L. Antimicrobial assay showed that the antimicrobial activity of GI24-W23A and GI24- W23K against gram-negative and gram-positive bacteria was significantly reduced. When the W of GI24 was replaced with L, the antimicrobial activity of GI24-W23L was recovered to a level similar to GI24. Our result may consist with the report of Lv *et al*. that the W at position 2 of our designed peptides may play a crucial role in antimicrobial activity and spectrum. The improvement of activity of the designed AMPs and the expansion of antimicrobial spectrum are ostensibly due to the connection of short peptides and the substitution of amino acids. The substance should be related to the mechanism of action of AMPs. After decades of intensive research, many theoretical hypotheses have been proposed to explain the process of AMPs inhibiting or killing microorganisms. However, there is no one to cover all kinds of AMPs mechanism hypothesis, and are not sure which hypothesis is closer to the real situation^46^. Generally, the AMPs’ ability to inhibit or kill microorganisms depends on their ability to interact with cell membranes or cell walls^47^. AMPs usually have a net positive charge and a high ratio of hydrophobic amino acids, allowing them to selectively bind to negatively charged cell membranes^10^. Binding of AMPs to the cell membrane leads to non-enzymatic disruption. Wang *et al*.^48^ found that AMPs with anti-gram-positive bacterial or anti-gram-negative bacterial generally possessed higher net charge and amphipathic values than their counterparts by using large-scale AMPs to examine the relationships between antimicrobial activities and two major physiochemical properties of AMPs—amphipathicity and net charge. How the designed AMPs specifically interact with bacteria needs further study, but it is usually considered that the changing of net charge and hydrophobicity of AMPs have a great influence on their activity level and antibacterial spectrum. Figure 7 shows the amphiphilic distribution and charge distribution of the parent peptide TPVVNPPFLQQT-NH_2_ and the designed peptide FWRRIRVTPVVNPWFLQQT-NH_2_. In structure prediction, we successfully obtained the 3D structure of 84 peptides by MD simulations, and later we will integrate these results (including the trajectory data of MD simulation, PDB file of representative structure, heat map, secondary Structure assumed by each amino acid) into our DRAMP database^11^. By analyzing the structure of these peptides, 73 out of the 84 peptides contain a stable helical structure. In the five designed peptides, FLRRIRVTPVVNPPFLQQT-NH_2_ shows a coil structure, while the other four contain stable helical structure, implying that the structure of helix is crucial for the peptides’ activity. In the process of sequence alignment, we identified a consensus sequence (FLRRIRV-NH_2_) present in several antimicrobial peptides. Meanwhile, the leucine at position 2 of the consensus sequence was transformed into tryptophan to effectively promote the anchoring of AMPs on the cell membrane, resulting in the peptide FWRRIRV-NH_2_. FWRRIRV-NH_2_ displayed higher antibacterial activity across *Listeria monocytogenes* (ATCC 19115) and *Staphylococcus aureus* (CMCC(B)26003) with MIC 32 μg/ml, 64 μg/ml, respectively, which will serve as a basis for iterative design of improved peptides. Based on the strengths of these designed peptides, this type of rational design will be useful for future assessments to develop and apply these peptides as novel antibiotics.

## Electronic supplementary material

Supplementary Figure S3

Dataset 1

Dataset 2
