# Supplementary material for: Novel 3D Structure Based Model for Activity Prediction and Design of Antimicrobial Peptides
Source: Sci Rep. 2018 Jul 25;8:11189. doi: 10.1038/s41598-018-29566-5 (PMC6060096; doi:10.1038/s41598-018-29566-5)
Supplement: Supplementary file 1 — Supplementary Figure S3 [file 41598_2018_29566_MOESM1_ESM.pdf]

Novel 3D Structure Based Model for Activity Prediction and Design of Antimicrobial Peptides

Shicai Liu, Jingxiao Bao, Xingzhen Lao* and Heng Zheng*
School of Life Science and Technology, China Pharmaceutical University, Nanjing 210009, China

* To whom correspondence should be addressed.
Corresponding author's contact information: Address: China Pharmaceutical University, 24 Tongjiaxiang, Nanjing, 210009, China. Tel: 86-25-83271001, Fax: 86-25-83271249, E-mail: lao@cpu.edu.cn (Xingzhen Lao), zhengh18@hotmail.com (Heng Zheng).

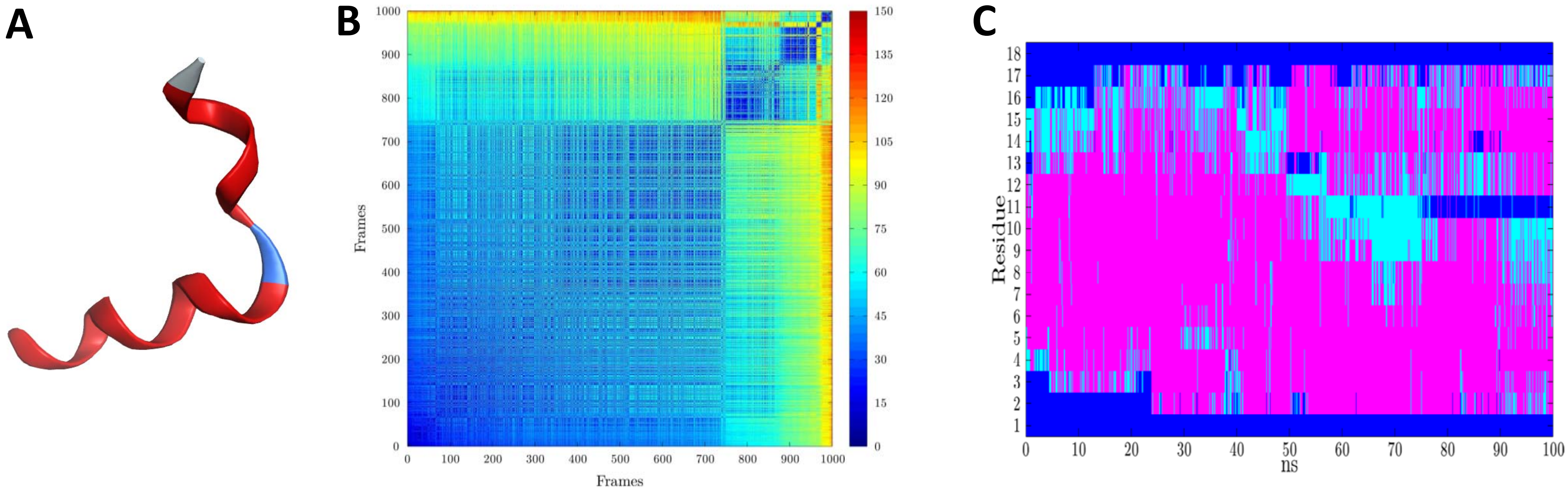

DRAMP00183

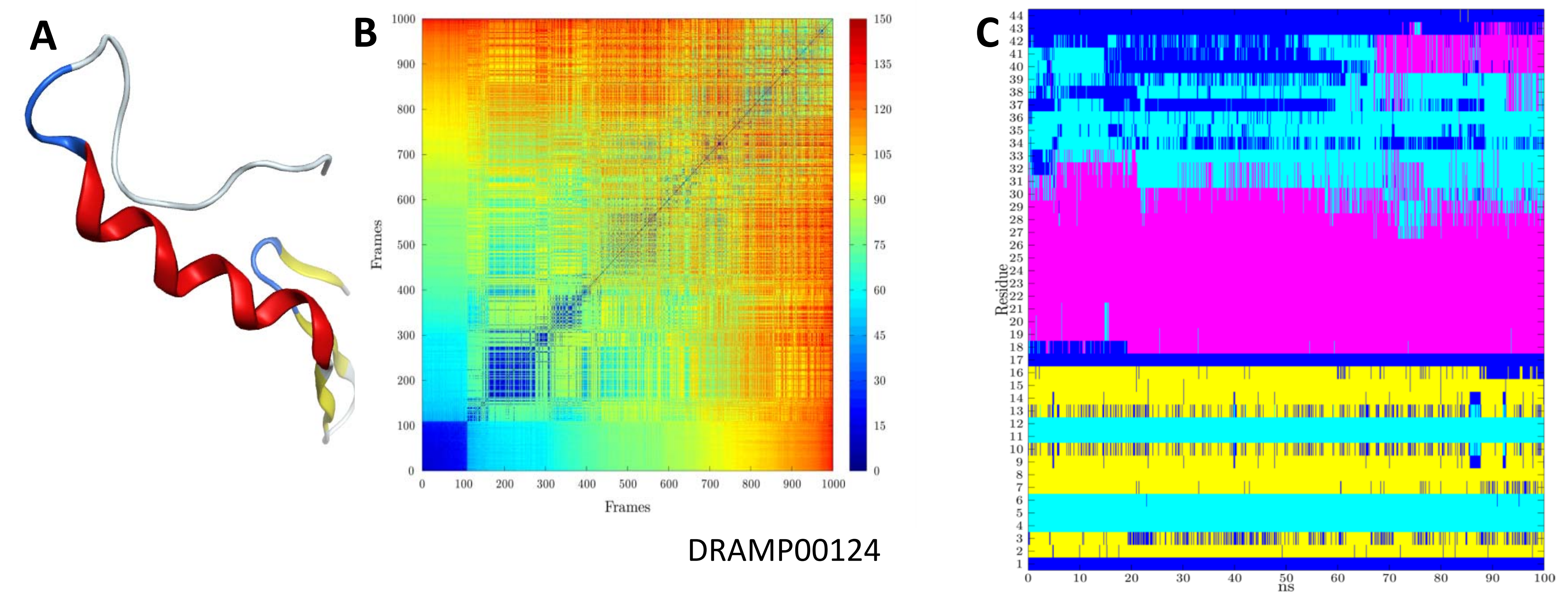
A
B
C
Frames
Frames
Residue
ns
DRAMP00124

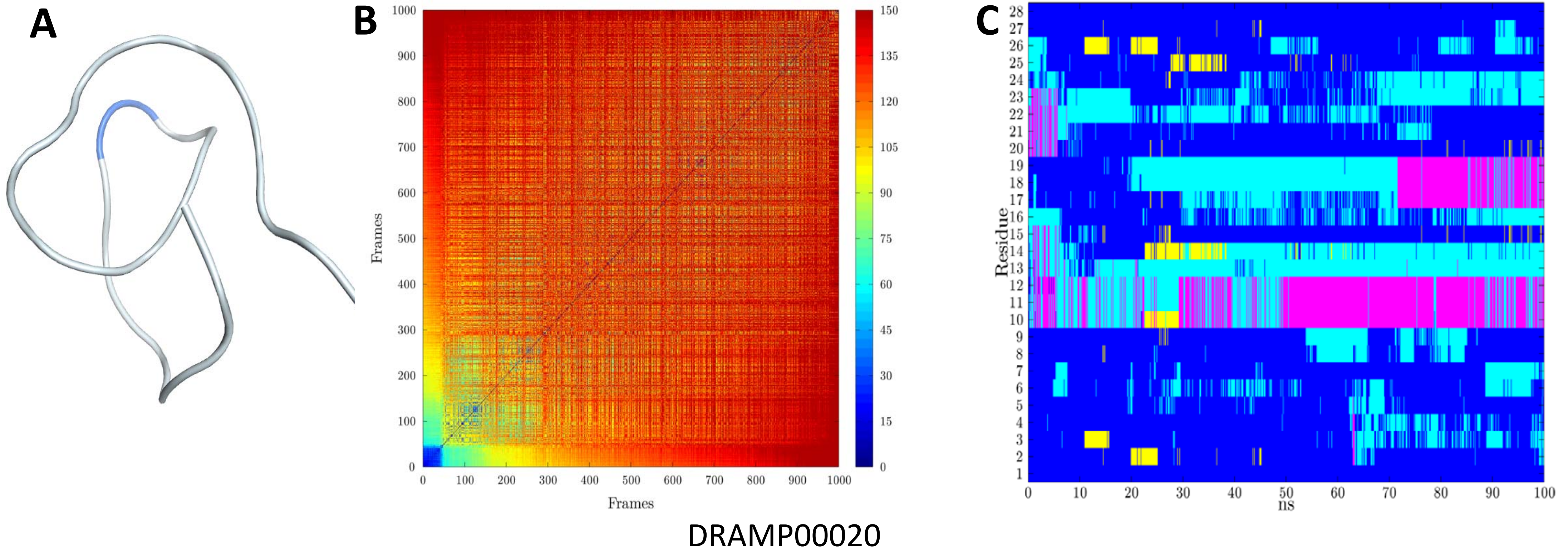
A
B
C
Frames
Frames
ns
Residue
DRAMP00020

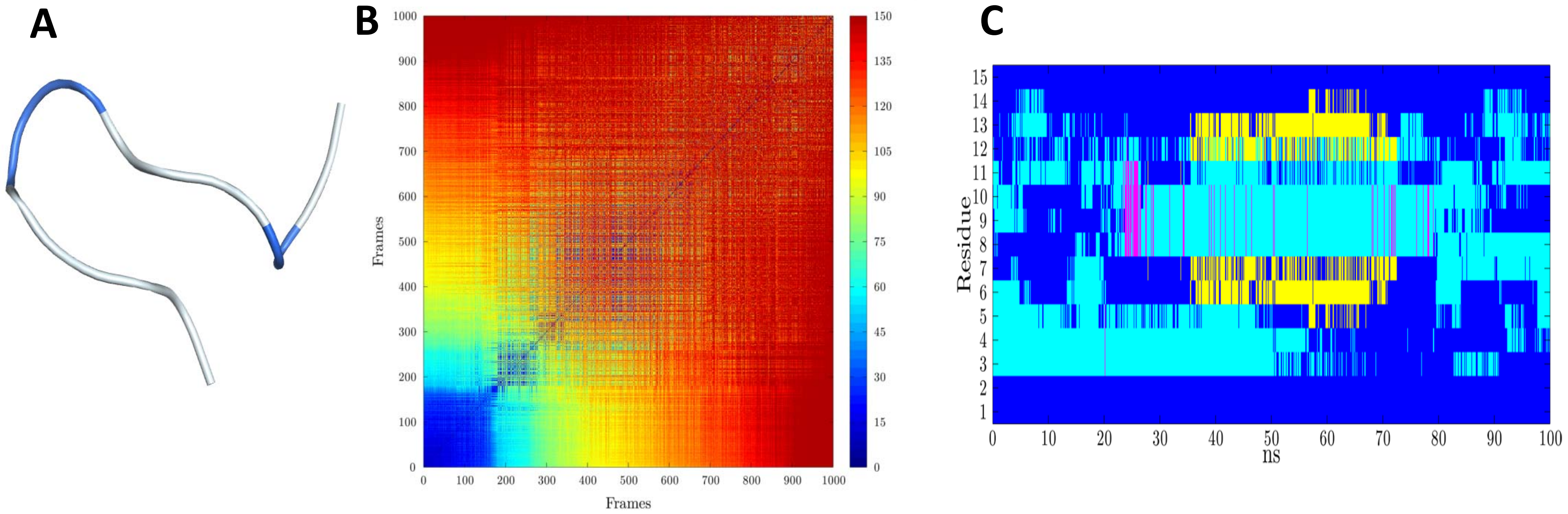
A
B
C
Frames
Frames
0
100
200
300
400
500
600
700
800
900
1000
150
135
120
105
90
75
60
45
30
15
0
Residue
ns
0
10
20
30
40
50
60
70
80
90
100

DRAMP18217

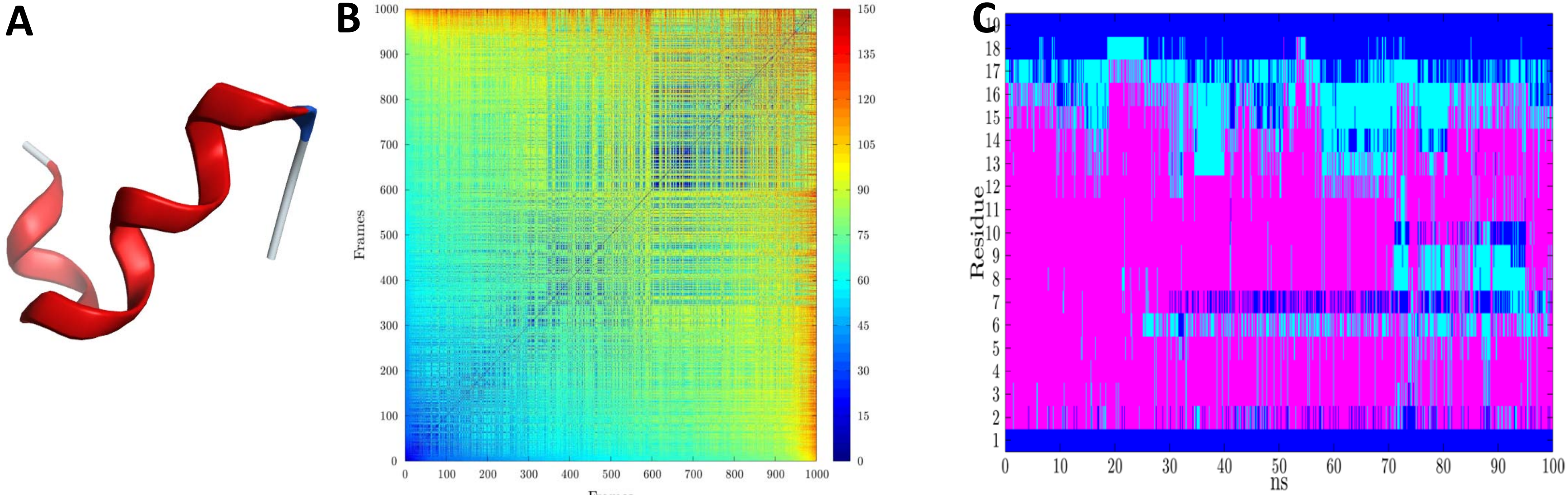
A
B
C
Frames
Frames
0
100
200
300
400
500
600
700
800
900
1000
150
135
120
105
90
75
60
45
30
15
0
Residue
ns
0
10
20
30
40
50
60
70
80
90
100

DRAMP18246

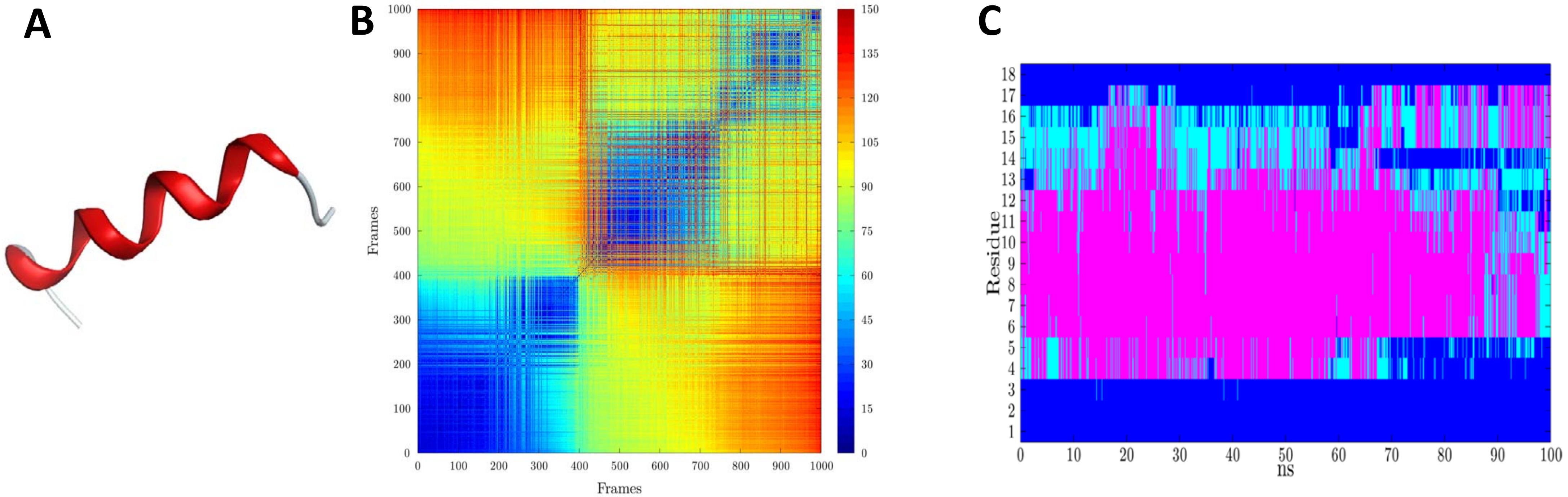

DRAMP00182

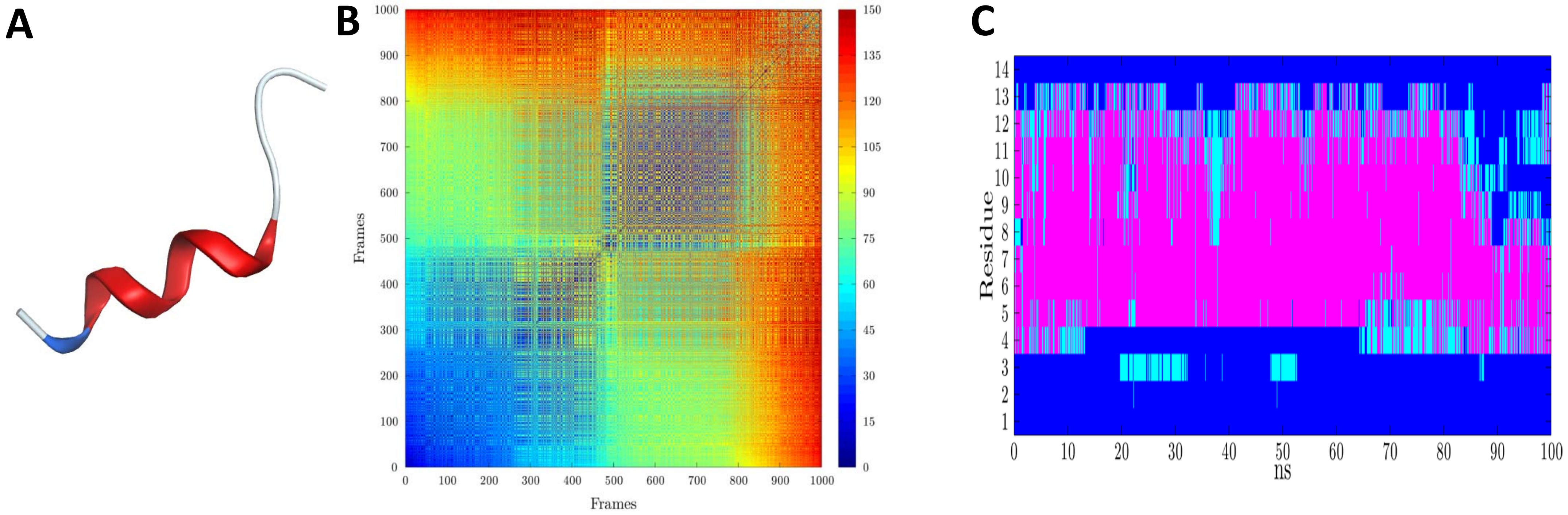
A
B
C
Frames
Frames
0
100
200
300
400
500
600
700
800
900
1000
150
135
120
105
90
75
60
45
30
15
0
Residue
ns
1
2
3
4
5
6
7
8
9
10
11
12
13
14
0
10
20
30
40
50
60
70
80
90
100

DRAMP00183

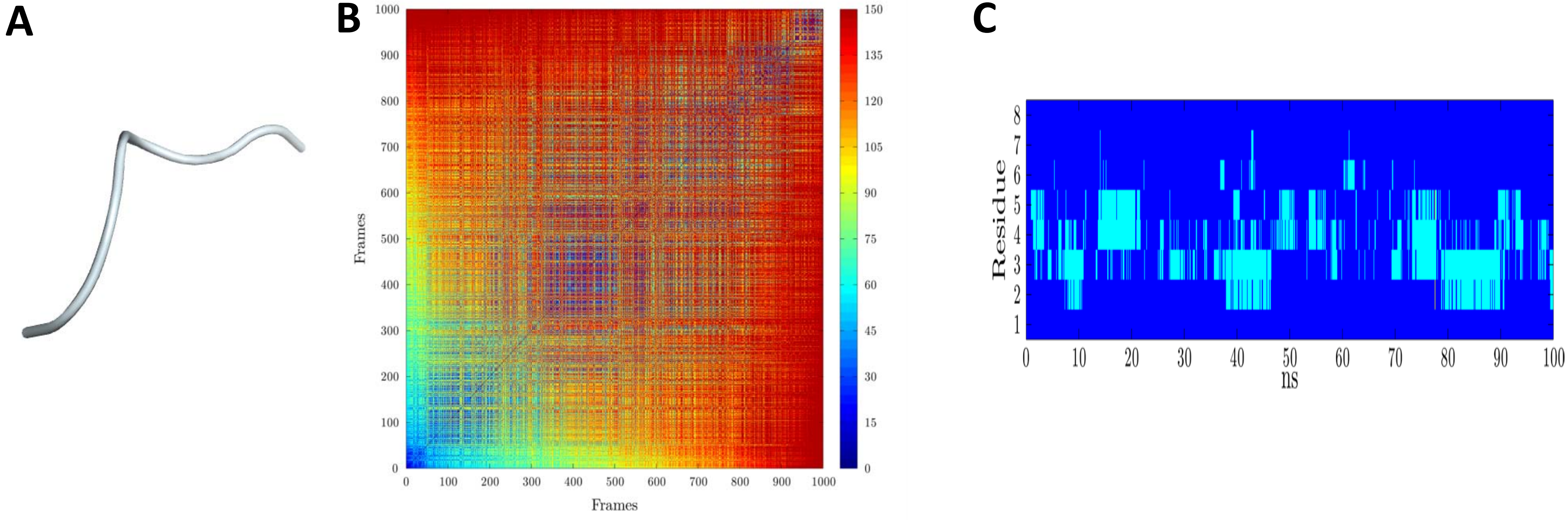

DRAMP18248

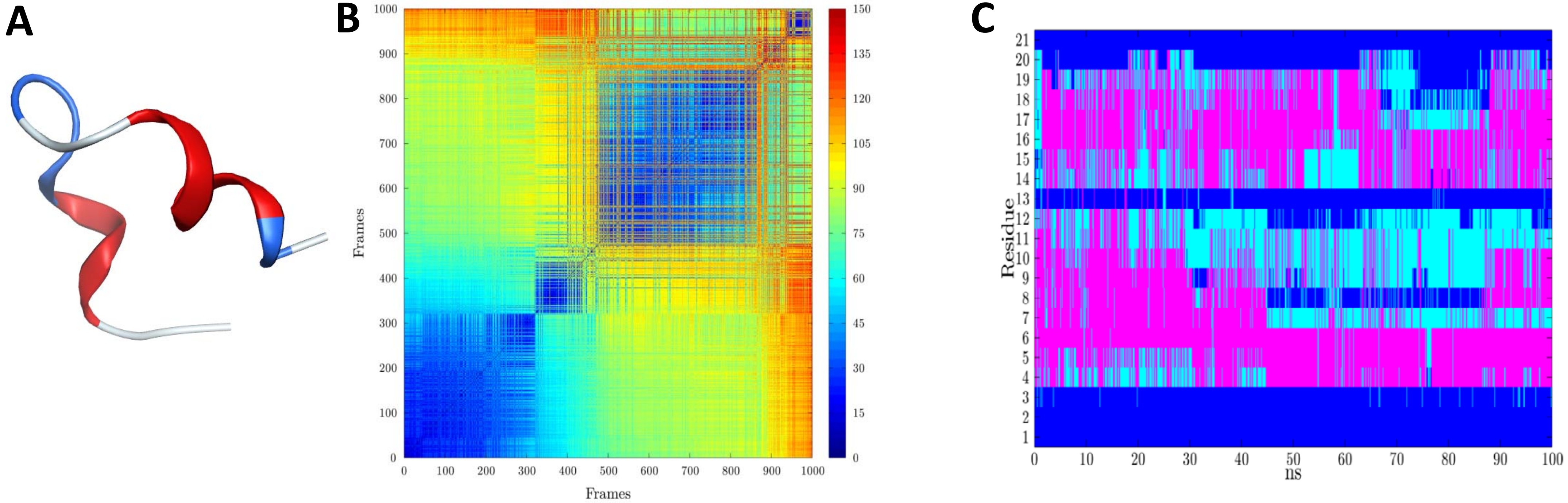

DRAMP18249

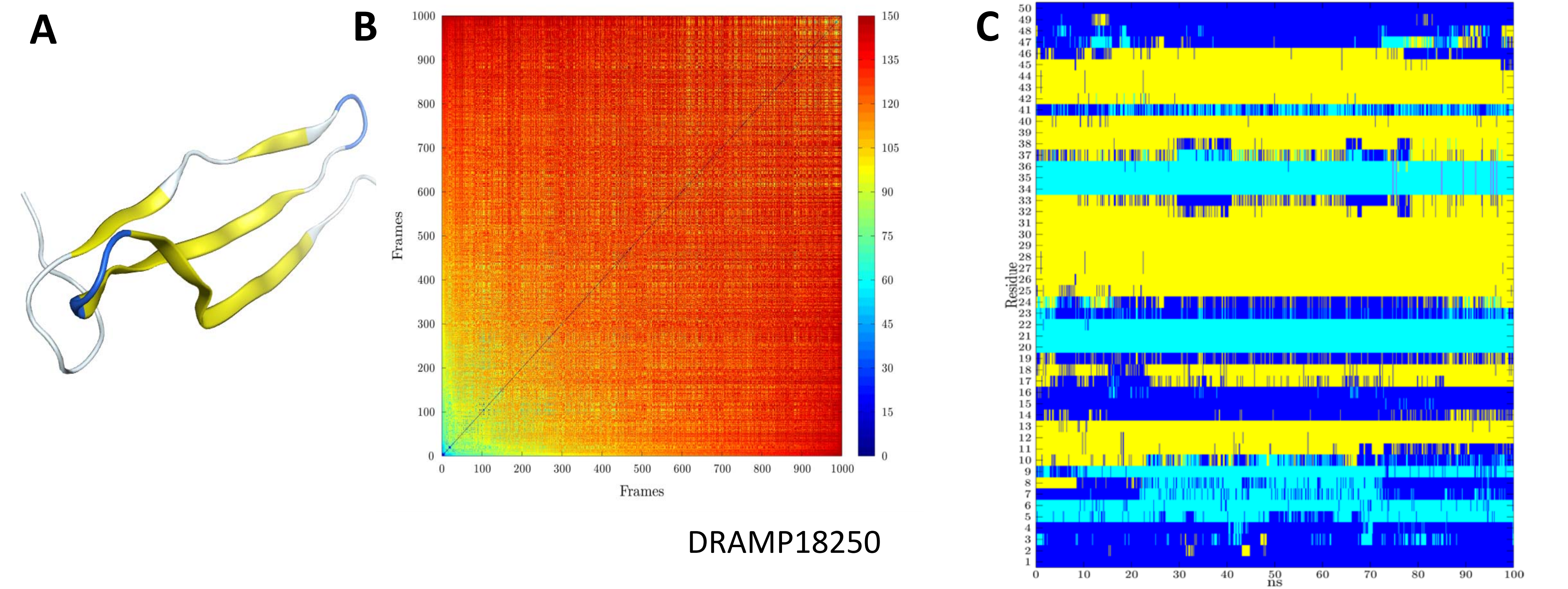
A
B
C
Frames
Frames
Residue
ns
DRAMP18250

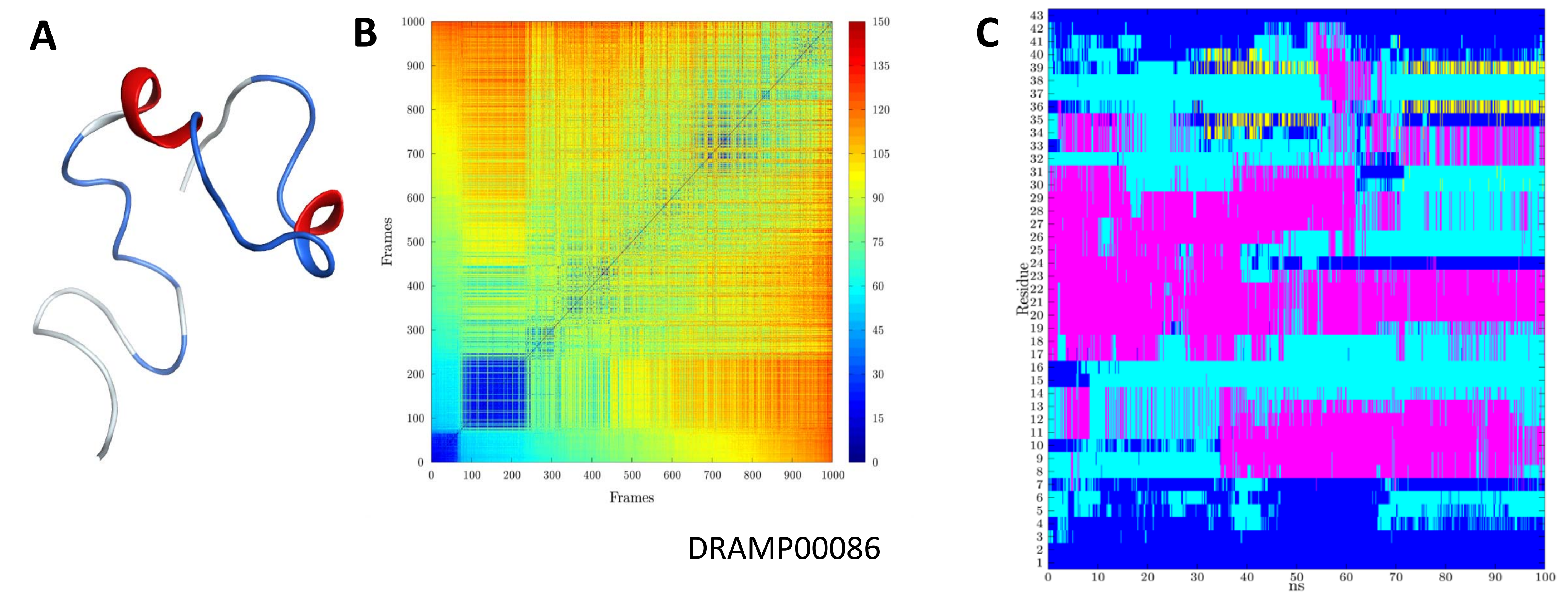
A
B
C
Frames
Frames
0
100
200
300
400
500
600
700
800
900
1000
150
135
120
105
90
75
60
45
30
15
0
Residue
ns
0
10
20
30
40
50
60
70
80
90
100
DRAMP00086

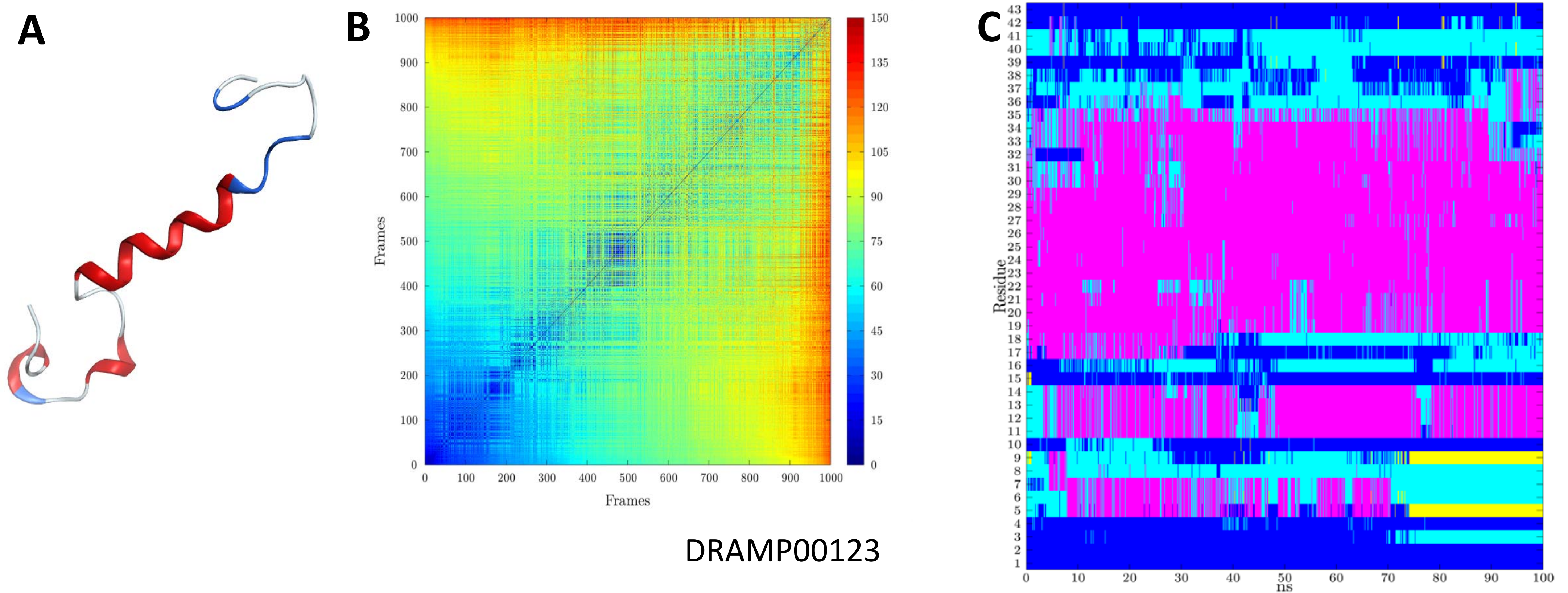

A
B
C
Frames
Frames
Residue
ns
DRAMP00123

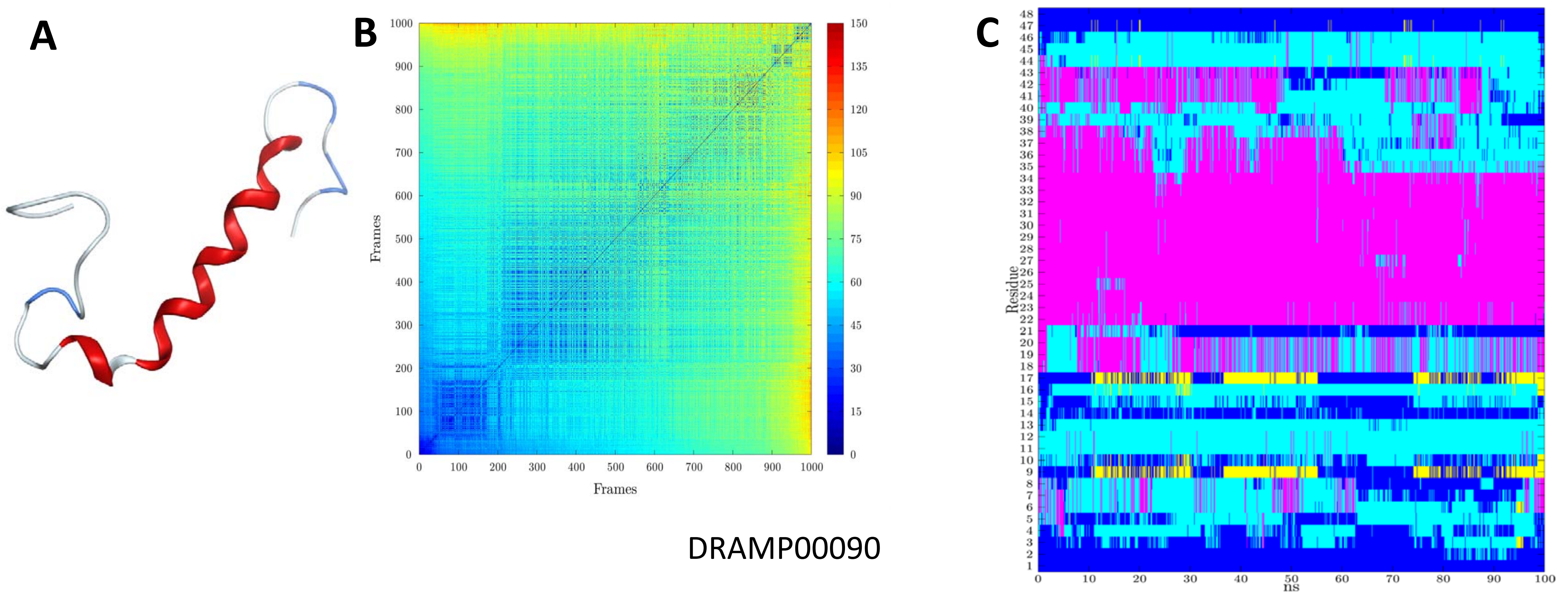

DRAMP00090

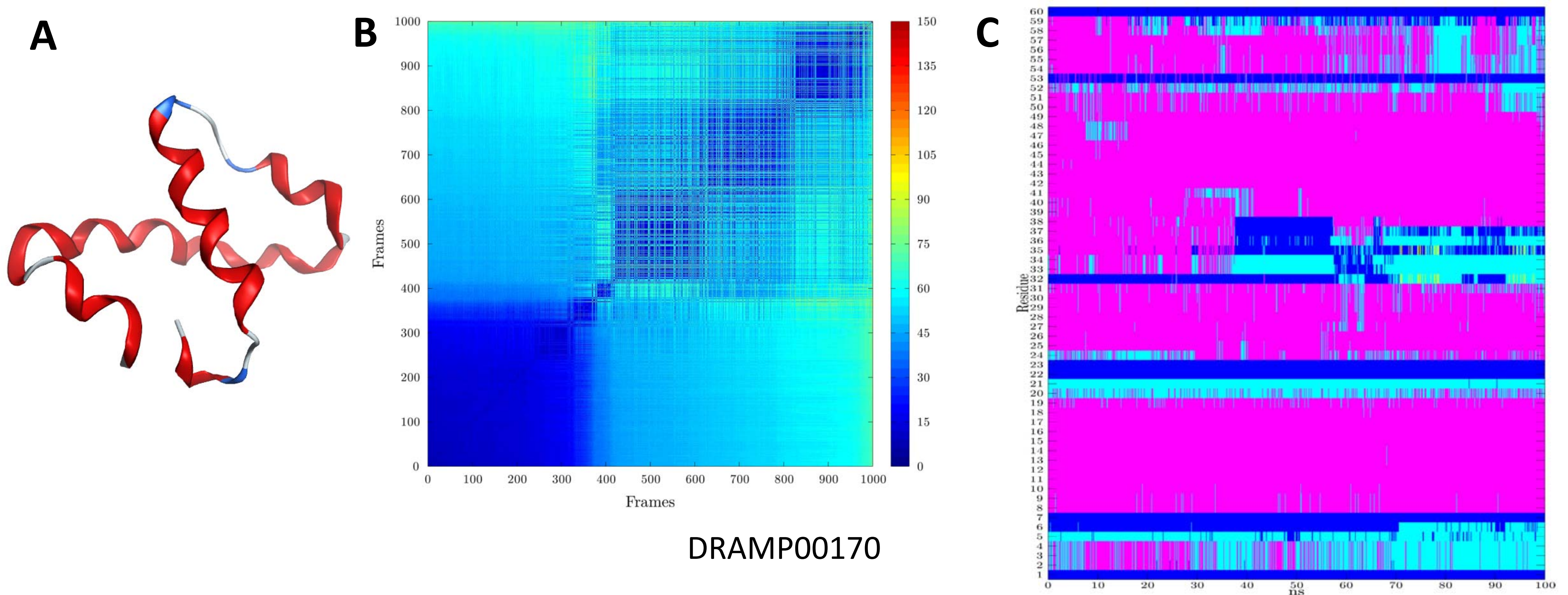
A
B
C
Frames
Residue
ns
DRAMP00170

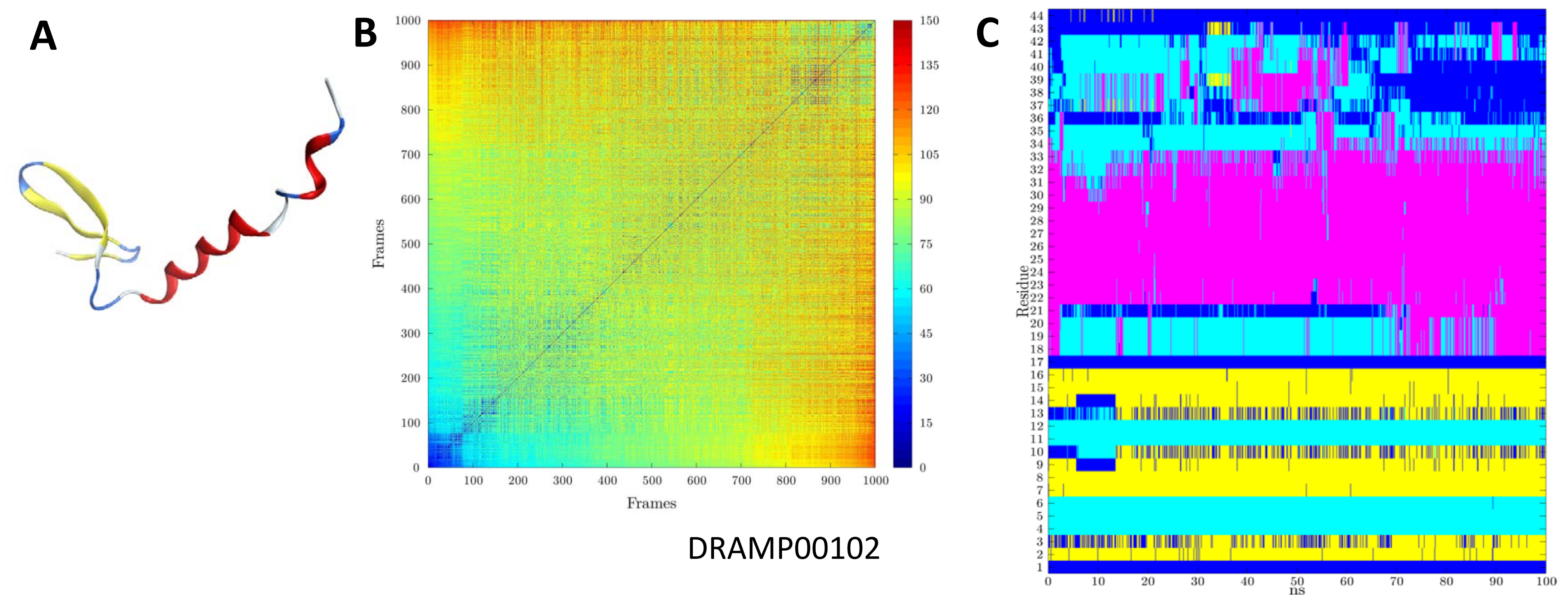

A
B
C
Frames
Frames
ns
Residue
DRAMP00102

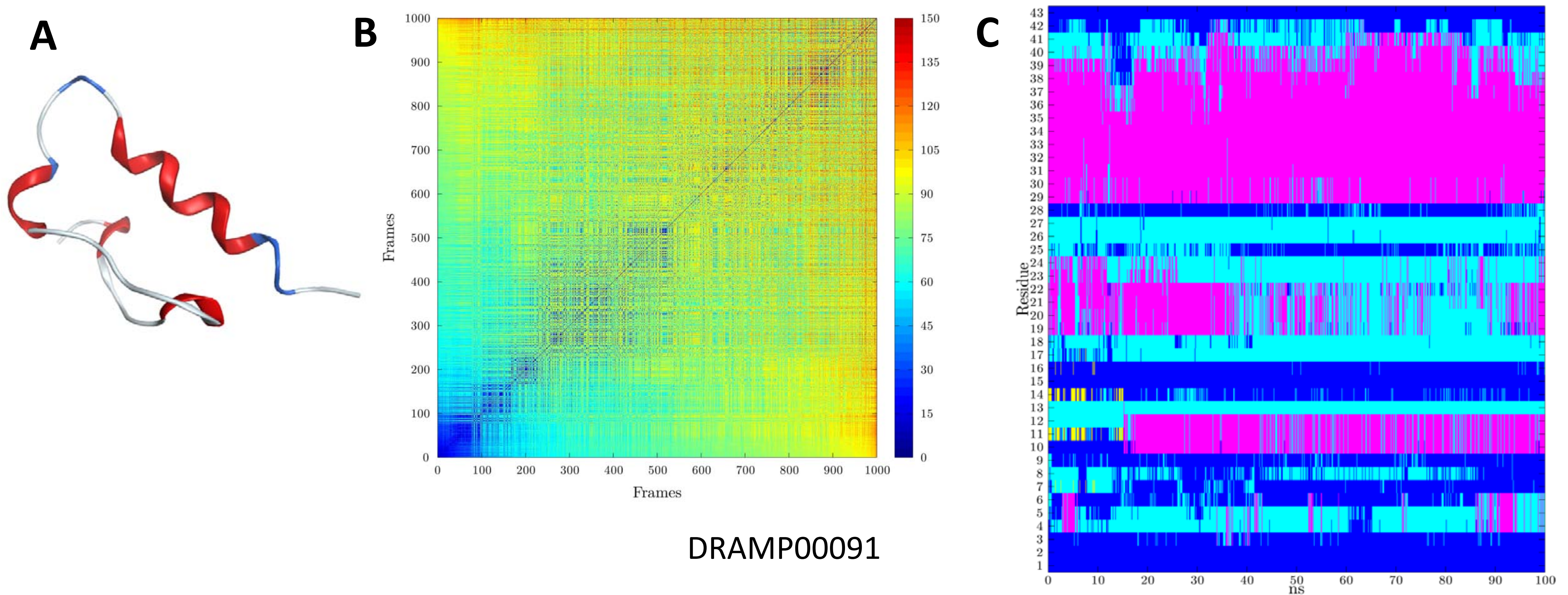
A
B
C
Frames
Frames
DRAMP00091
Residue
ns

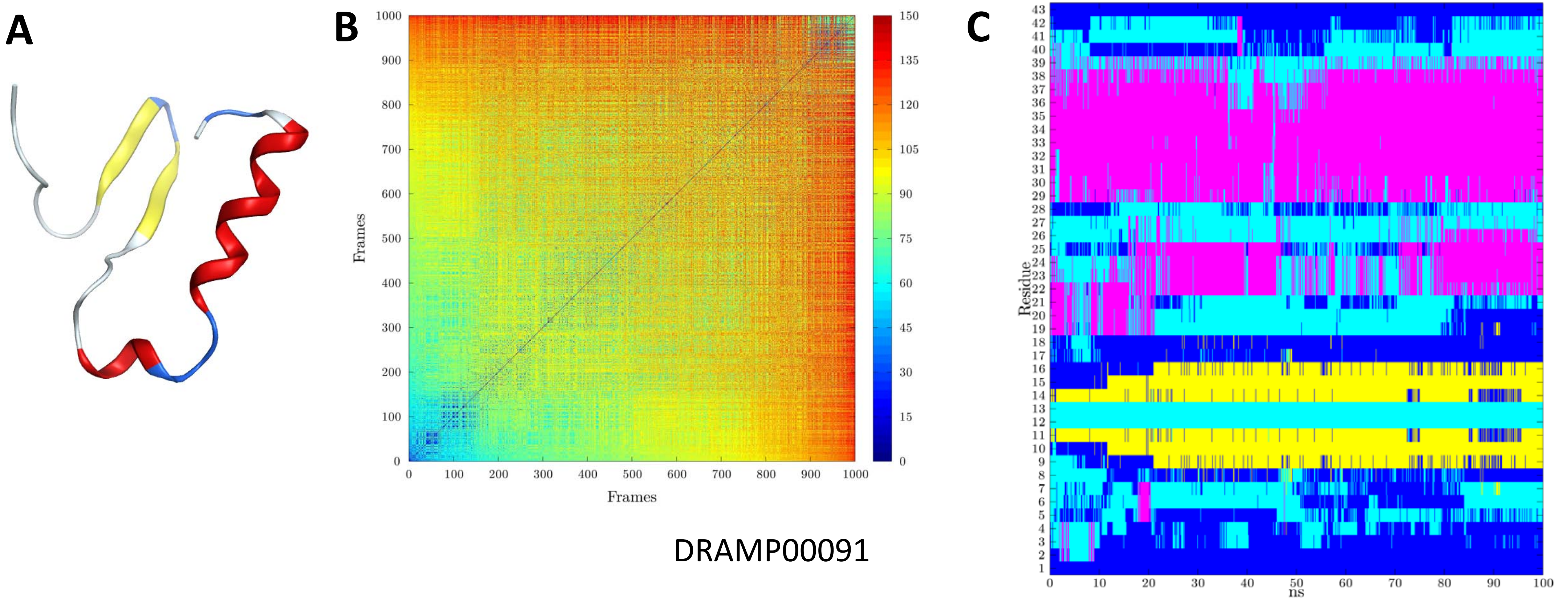

DRAMP00091

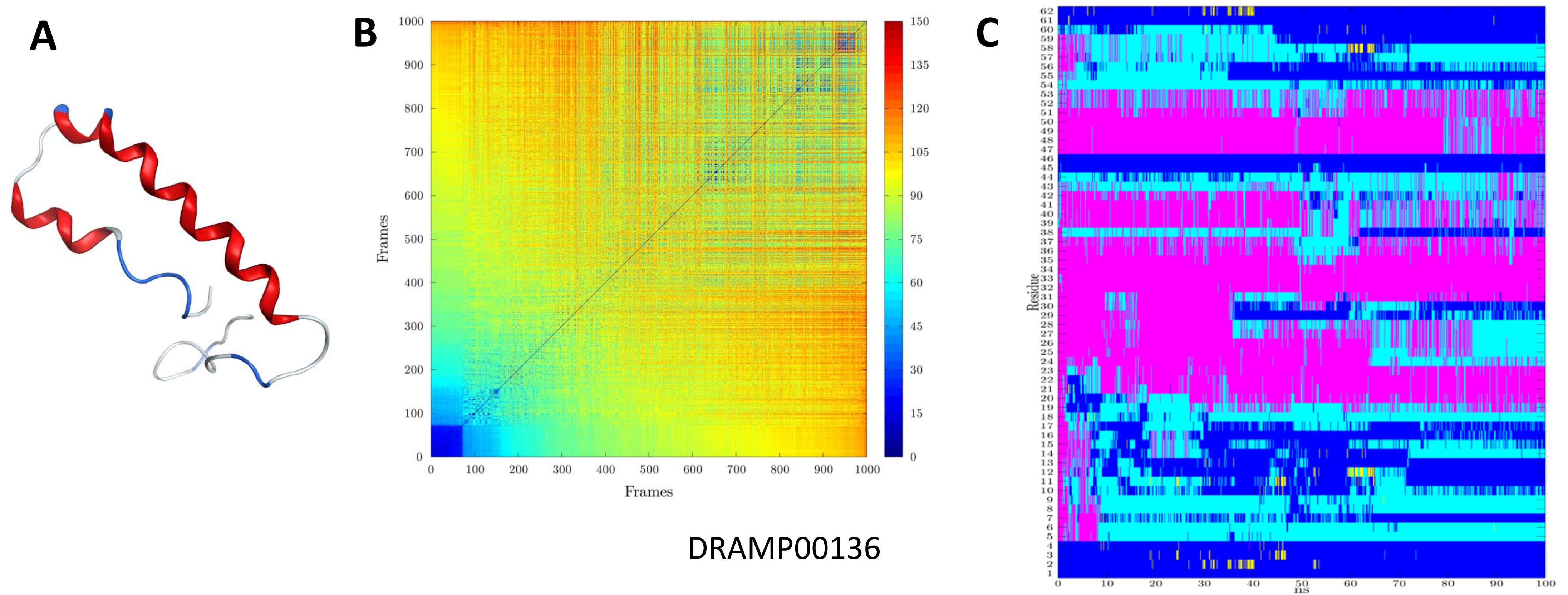
A
B
C
Frames
Frames
Residue
ns
DRAMP00136

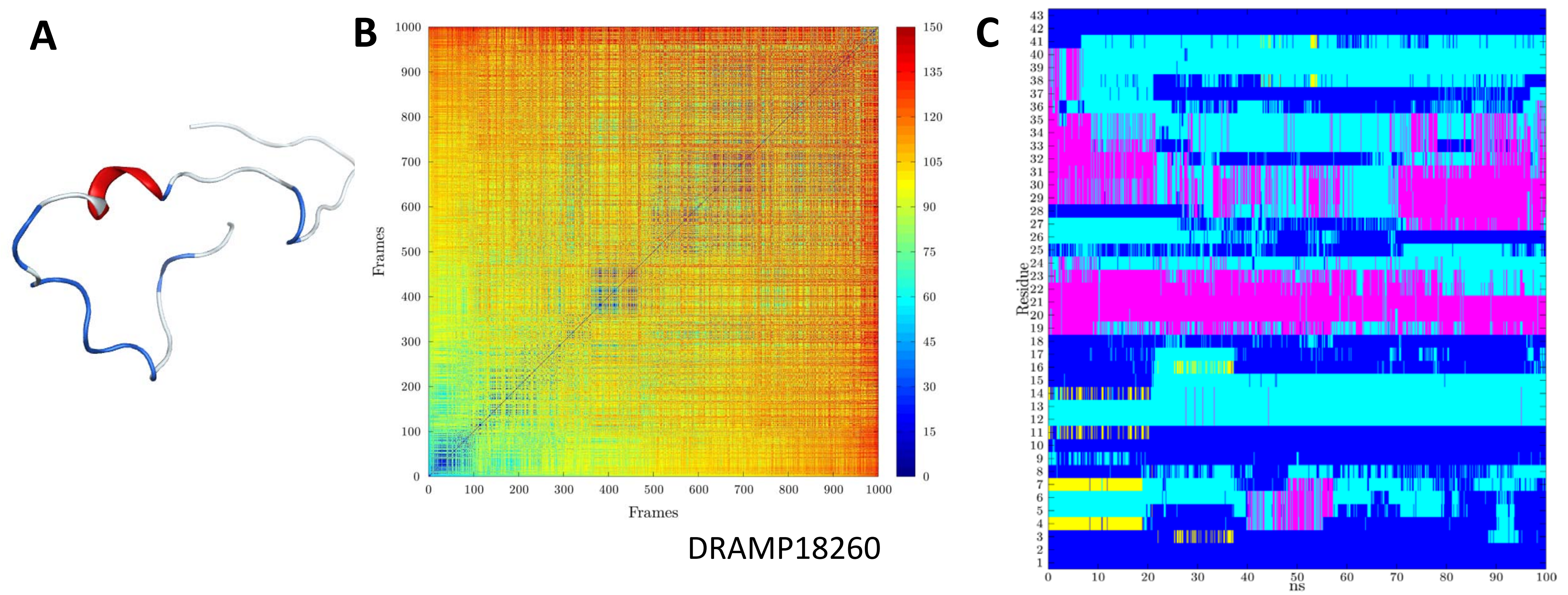
A
B
C
Frames
Frames
DRAMP18260
Residue
ns

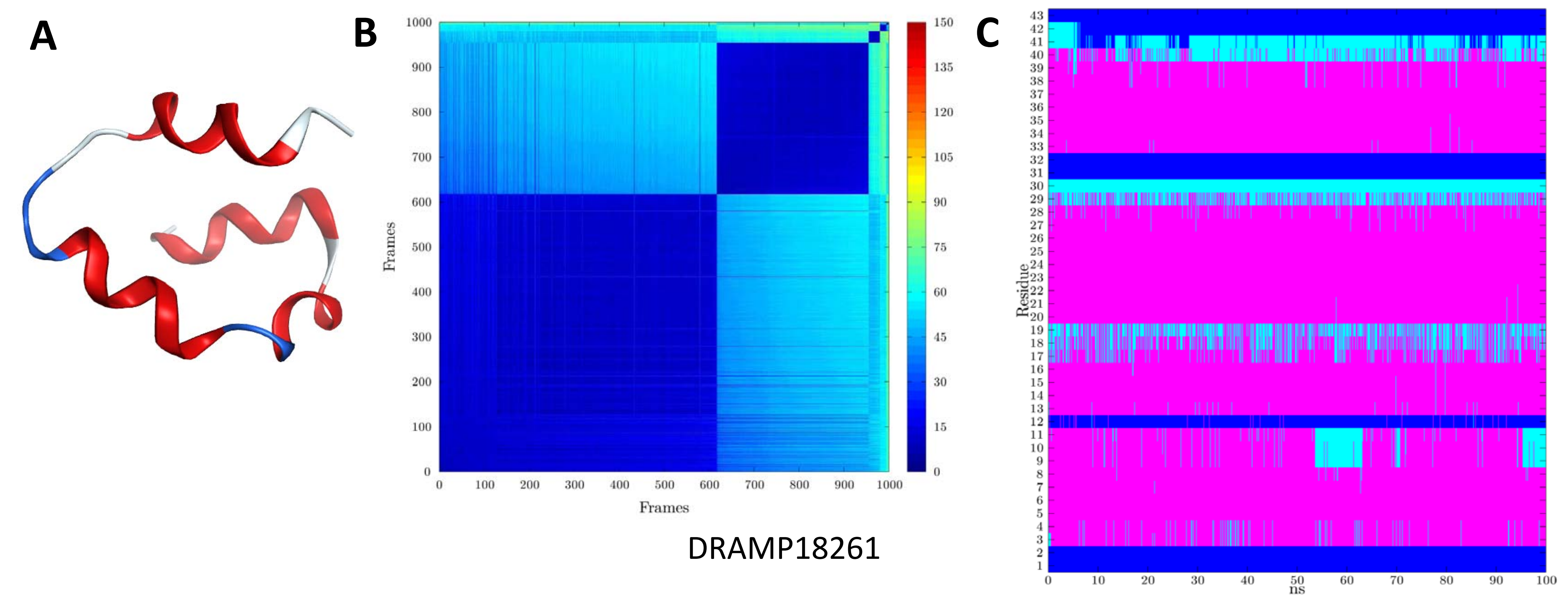

A
B
C
Frames
Residue
ns
DRAMP18261

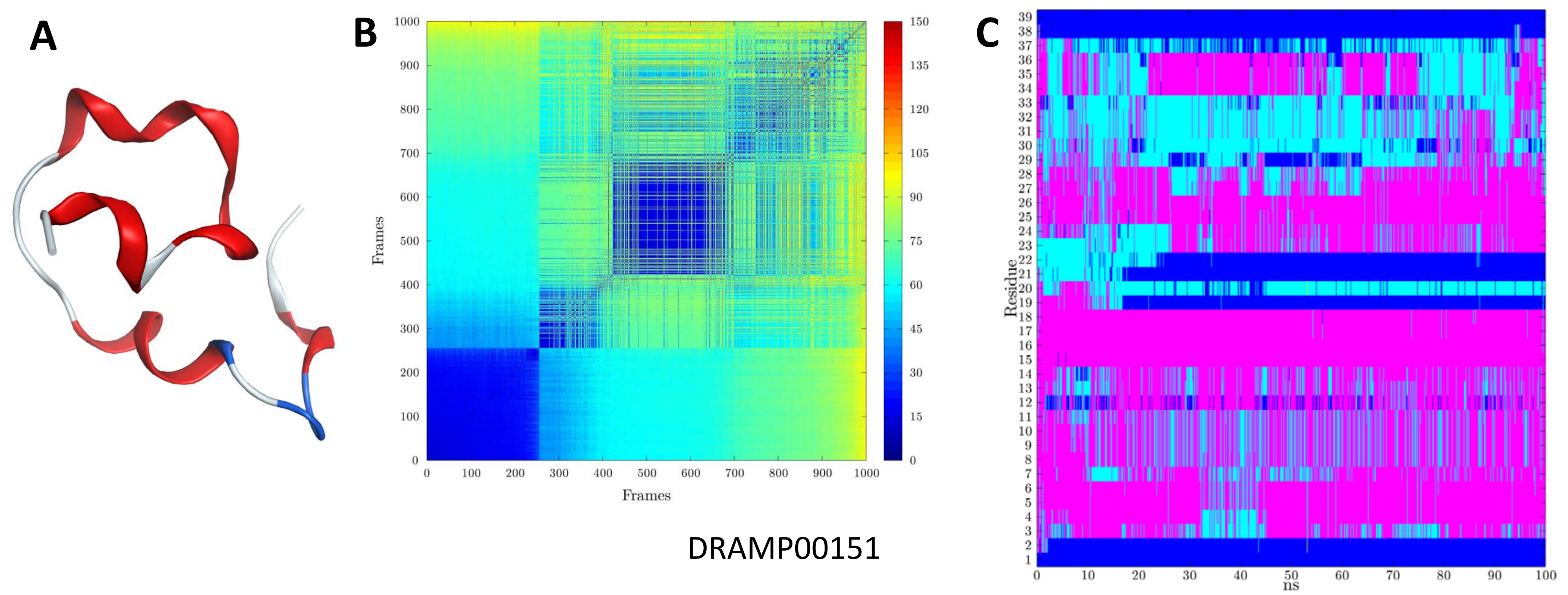

DRAMP00151

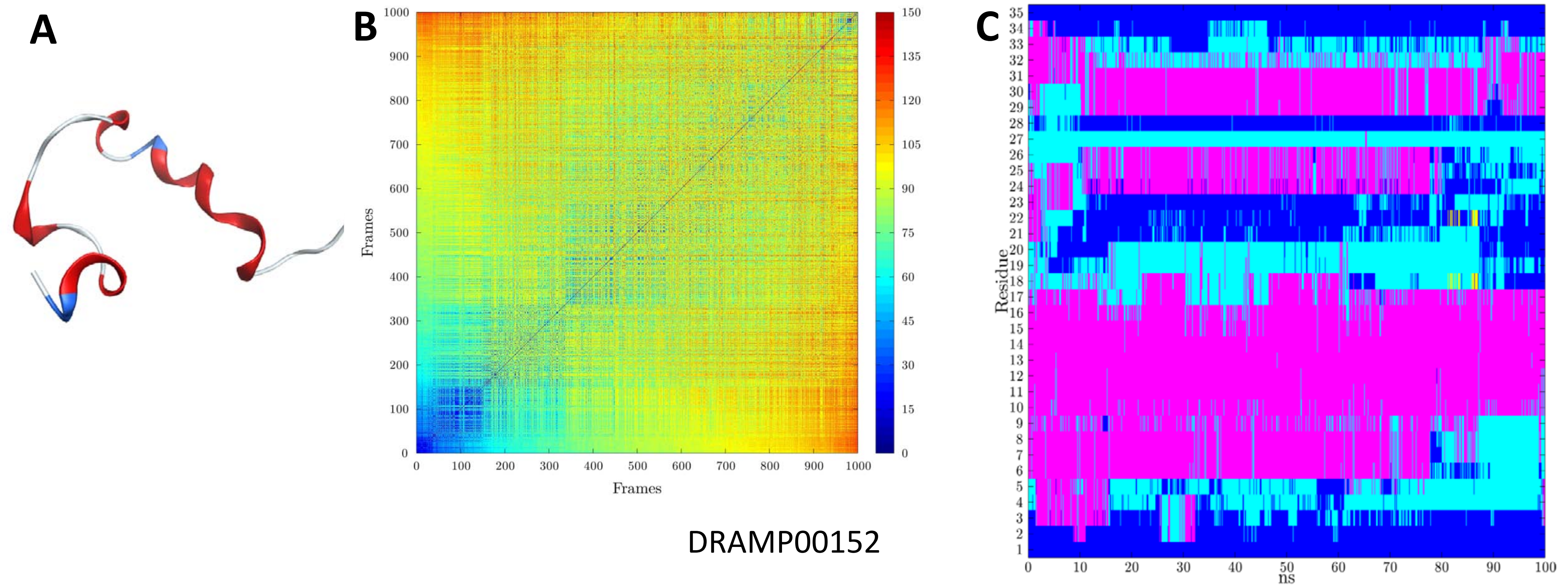
A
B
C
Frames
Frames
Residue
ns
DRAMP00152

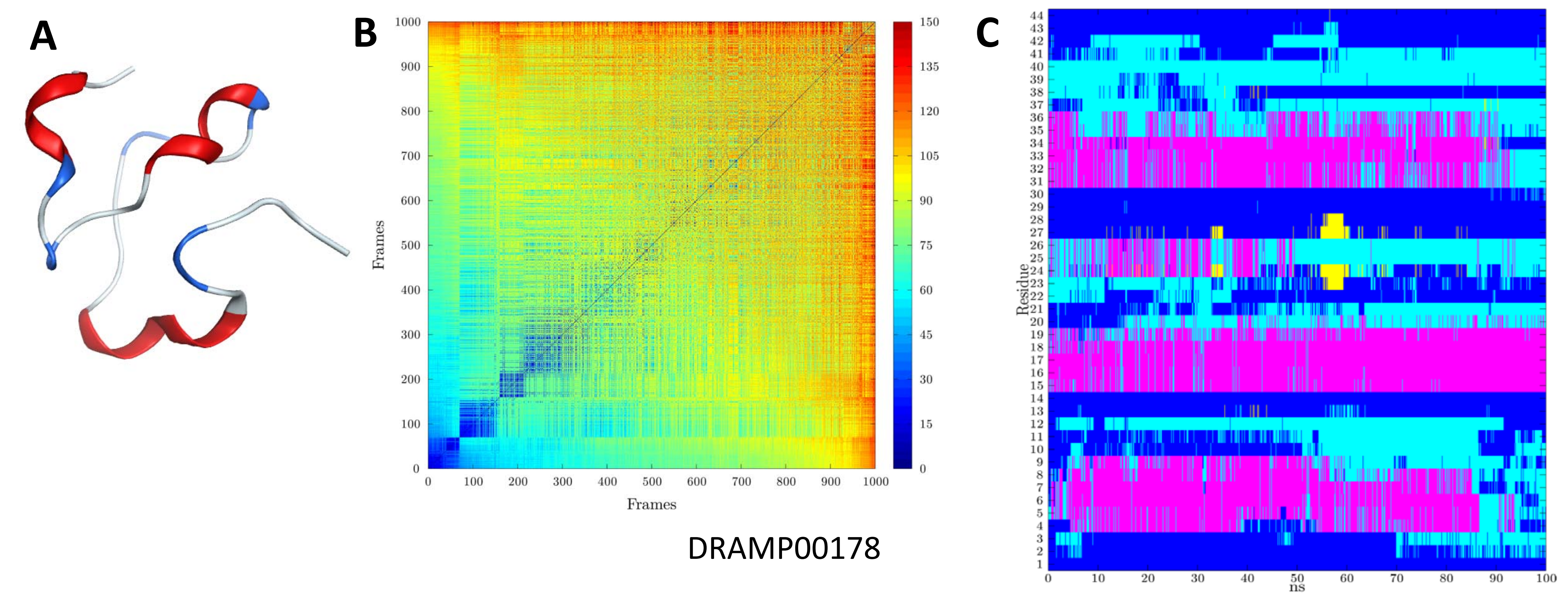

A
B
C
Frames
Frames
Residue
ns
DRAMP00178

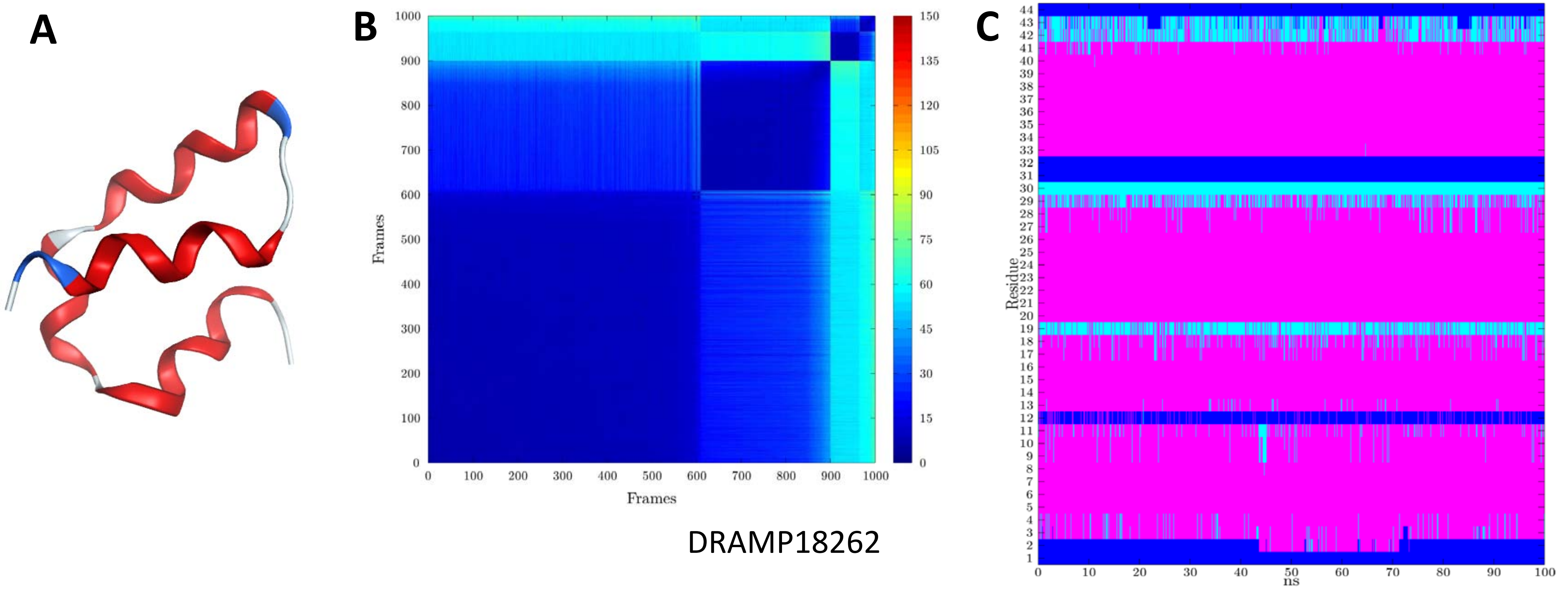
A
B
C
Frames
Frames
Residue
ns
DRAMP18262

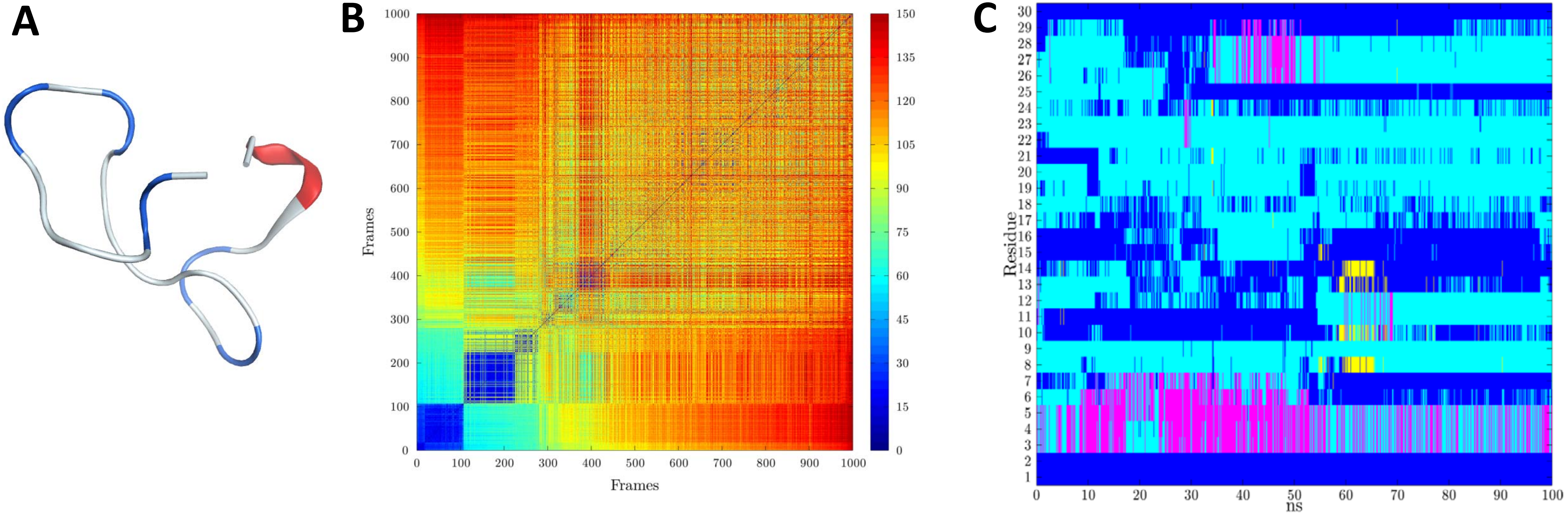
A
B
C
Frames
Frames
Residue
ns
0
100
200
300
400
500
600
700
800
900
1000
150
135
120
105
90
75
60
45
30
15
0
10
20
30
40
50
60
70
80
90
100

DRAMP18263

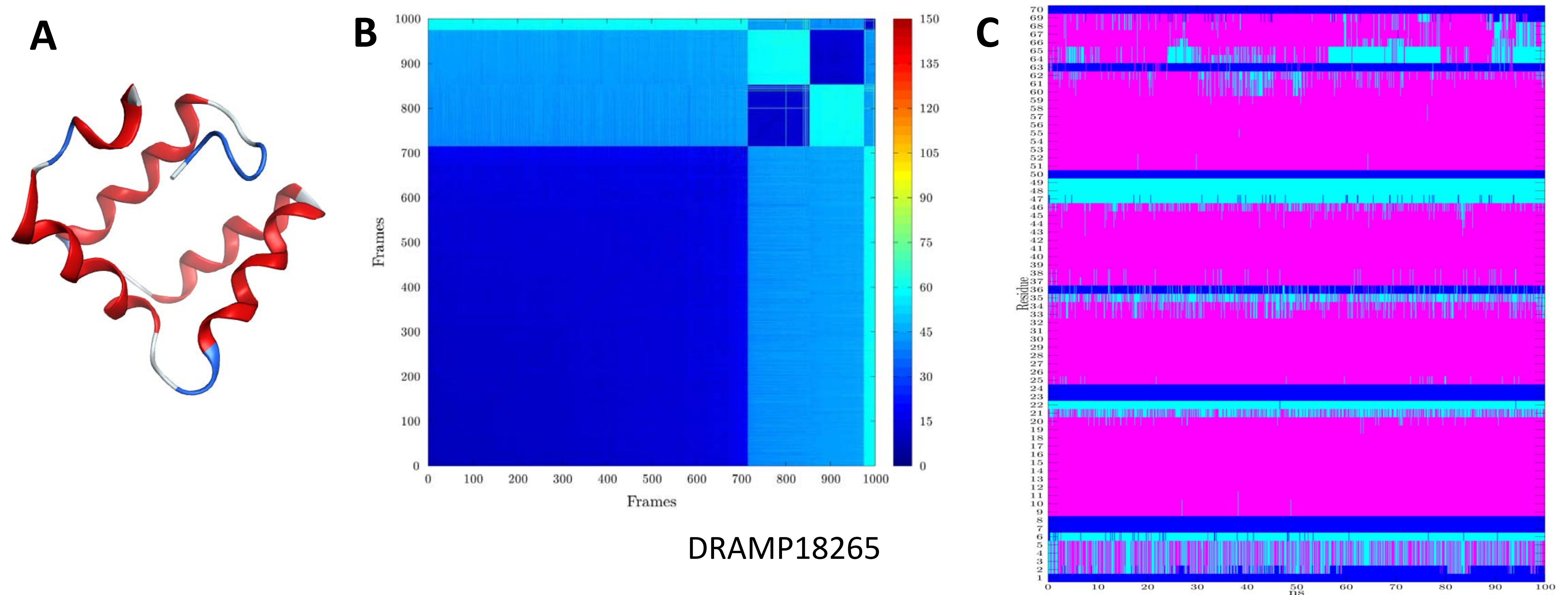
A
B
C
Frames
Frames
Residue
ns
DRAMP18265

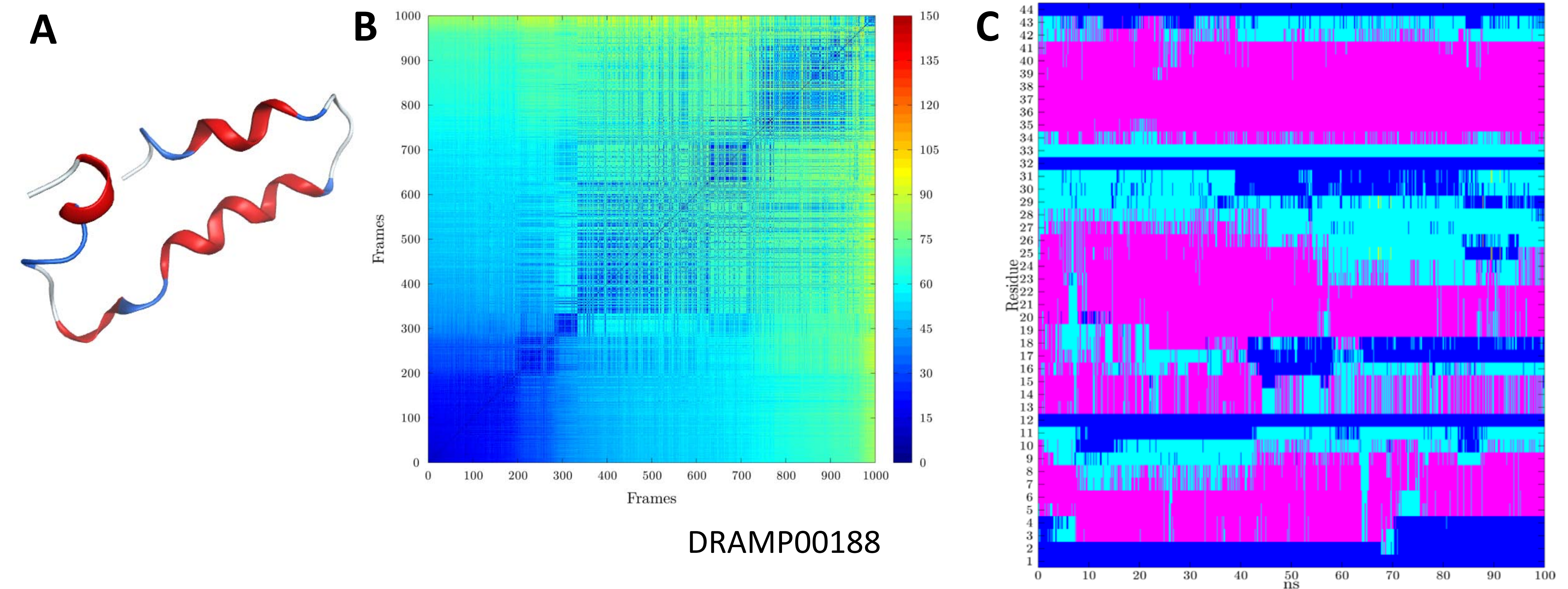

A
B
C
Frames
Frames
Residue
ns
DRAMP00188

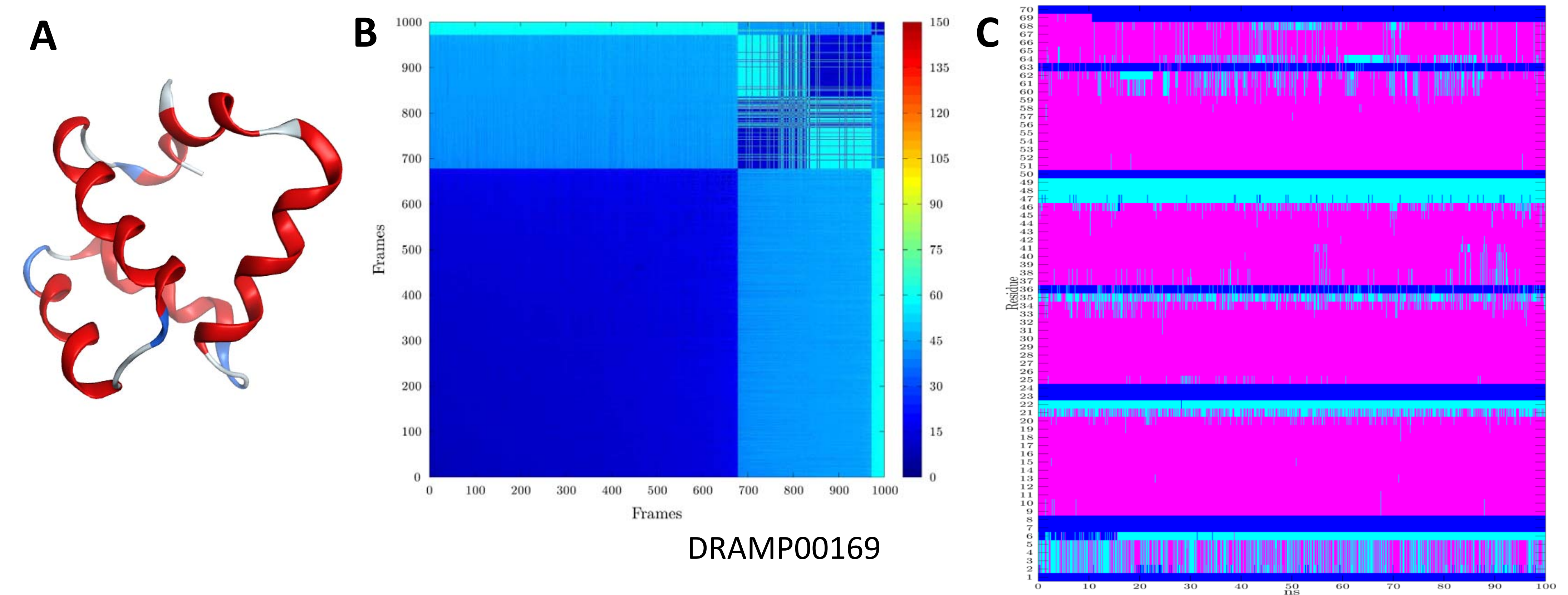

A
B
C
Frames
Frames
0 100 200 300 400 500 600 700 800 900 1000
0 15 30 45 60 75 90 105 120 135 150
DRAMP00169
Residue
ns
0 10 20 30 40 50 60 70 80 90 100

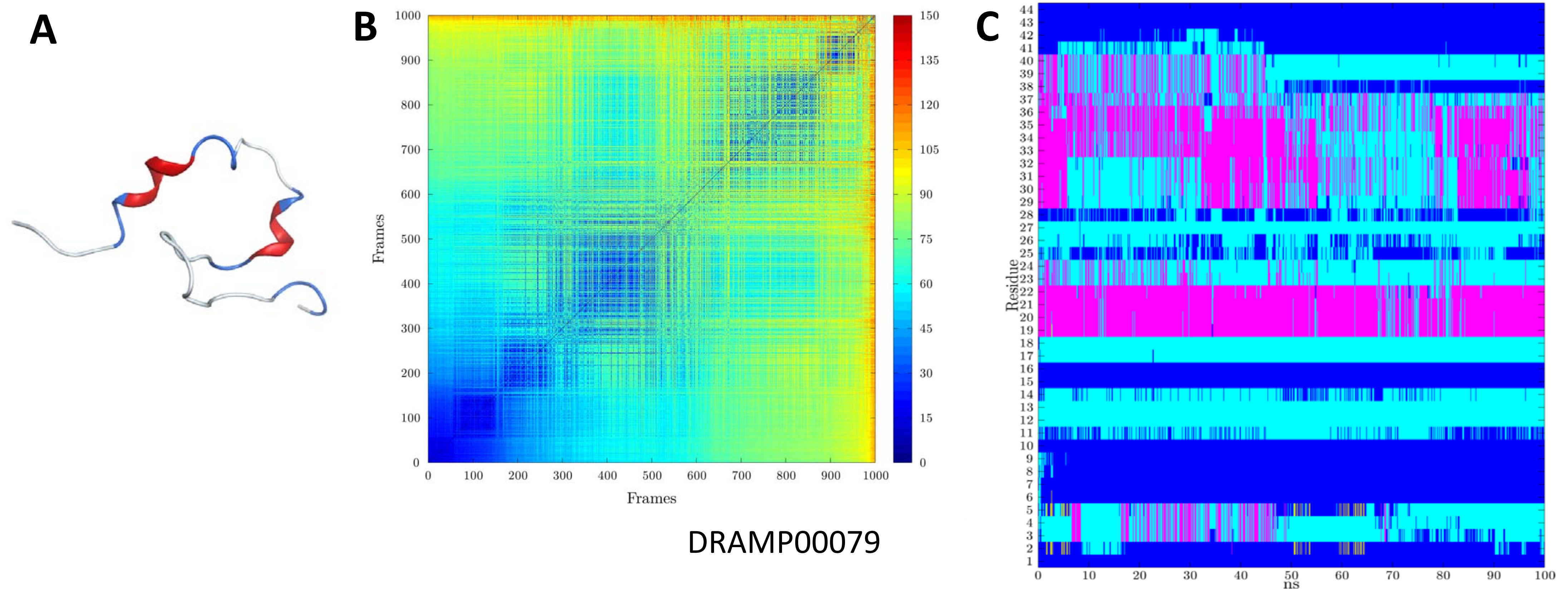
A
B
C
Frames
Residue
ns
DRAMP00079

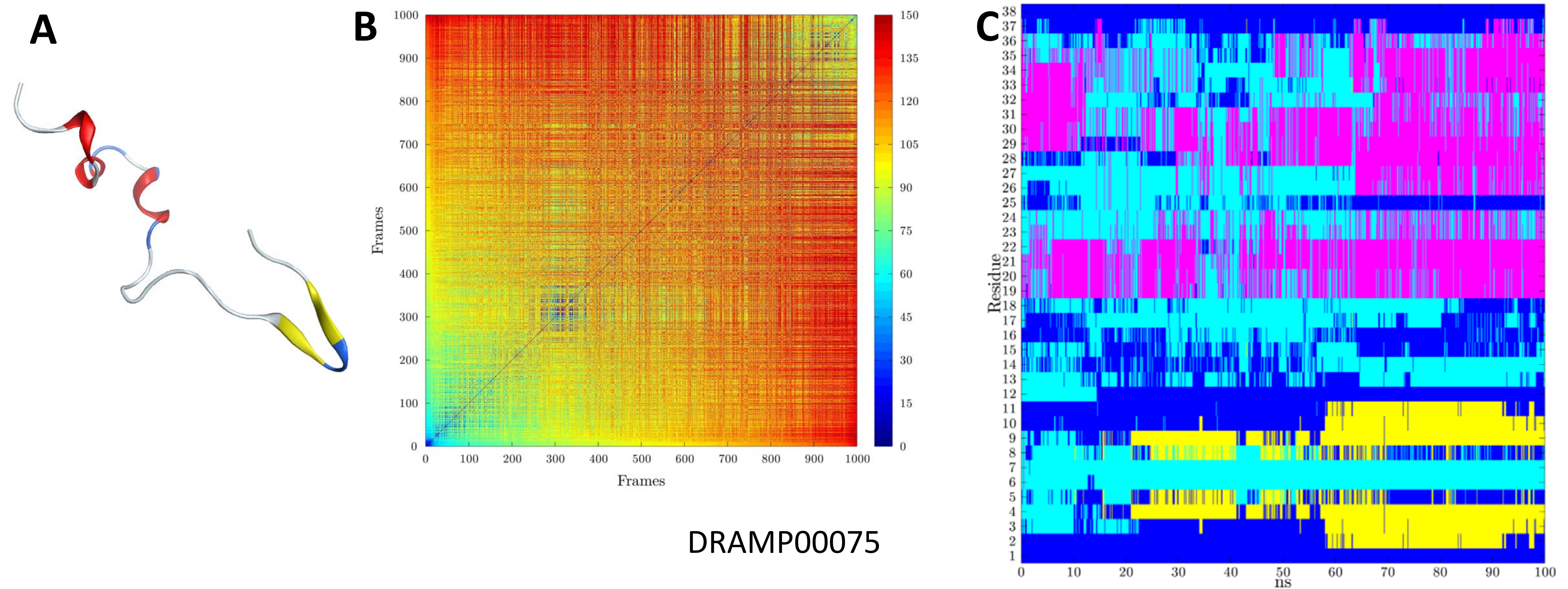
A
B
C
Frames
Frames
Residue
ns
DRAMP00075

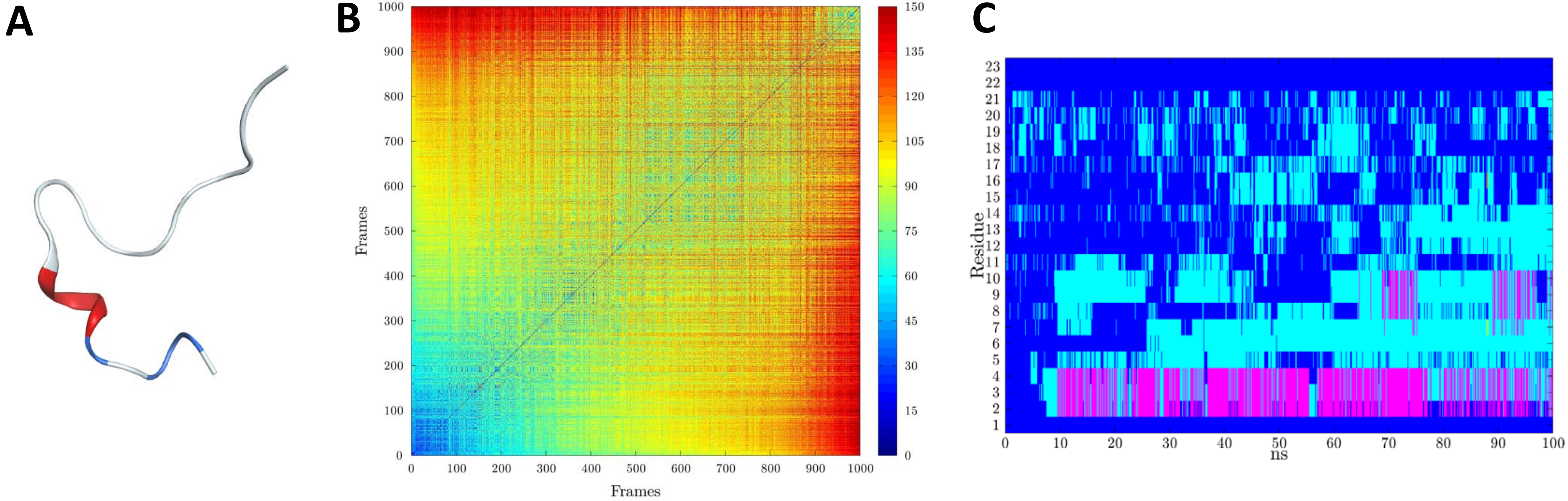

DRAMP18267

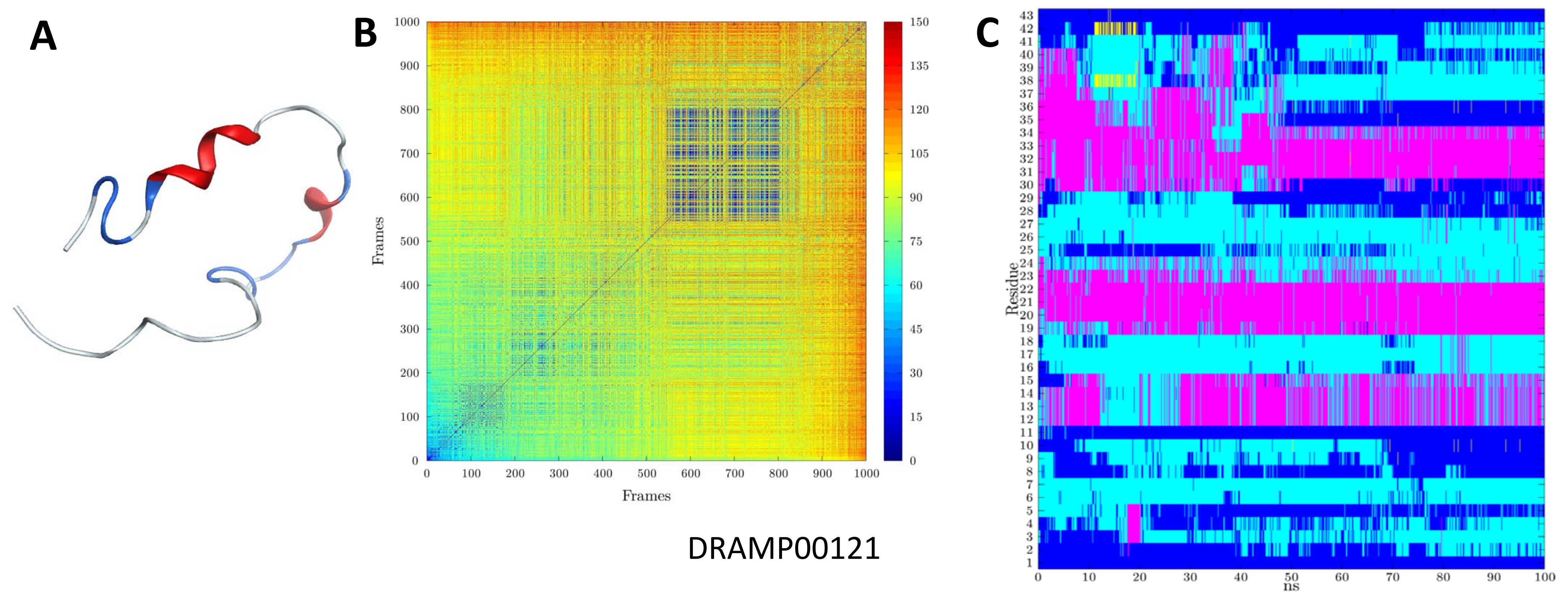

A
B
C
Frames
Frames
Residue
ns
DRAMP00121

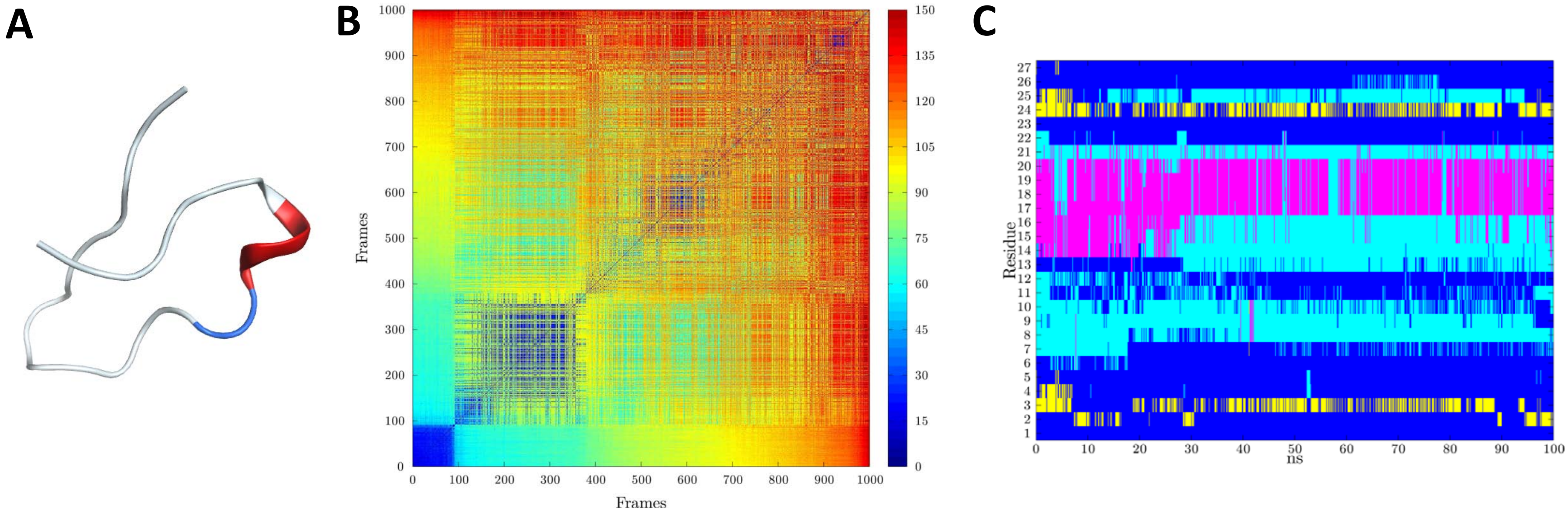

DRAMP00239

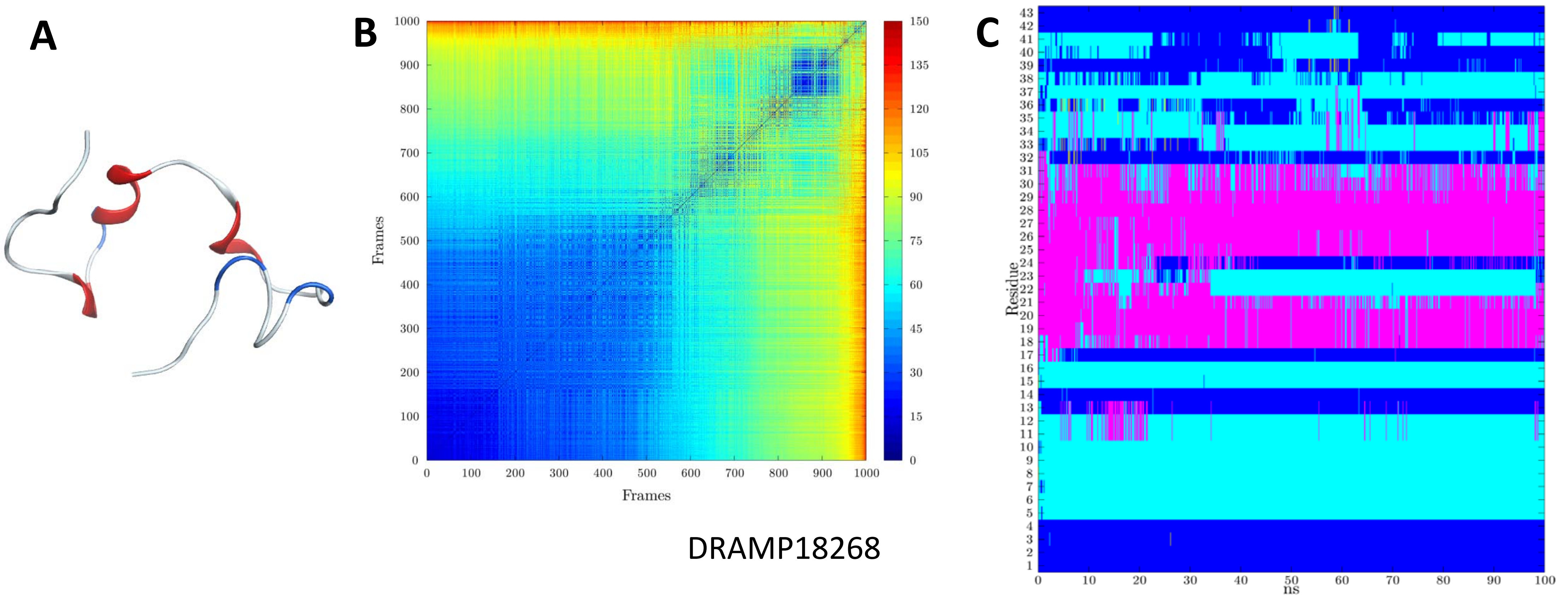
A
B
C
Frames
Frames
DRAMP18268
Residue
ns

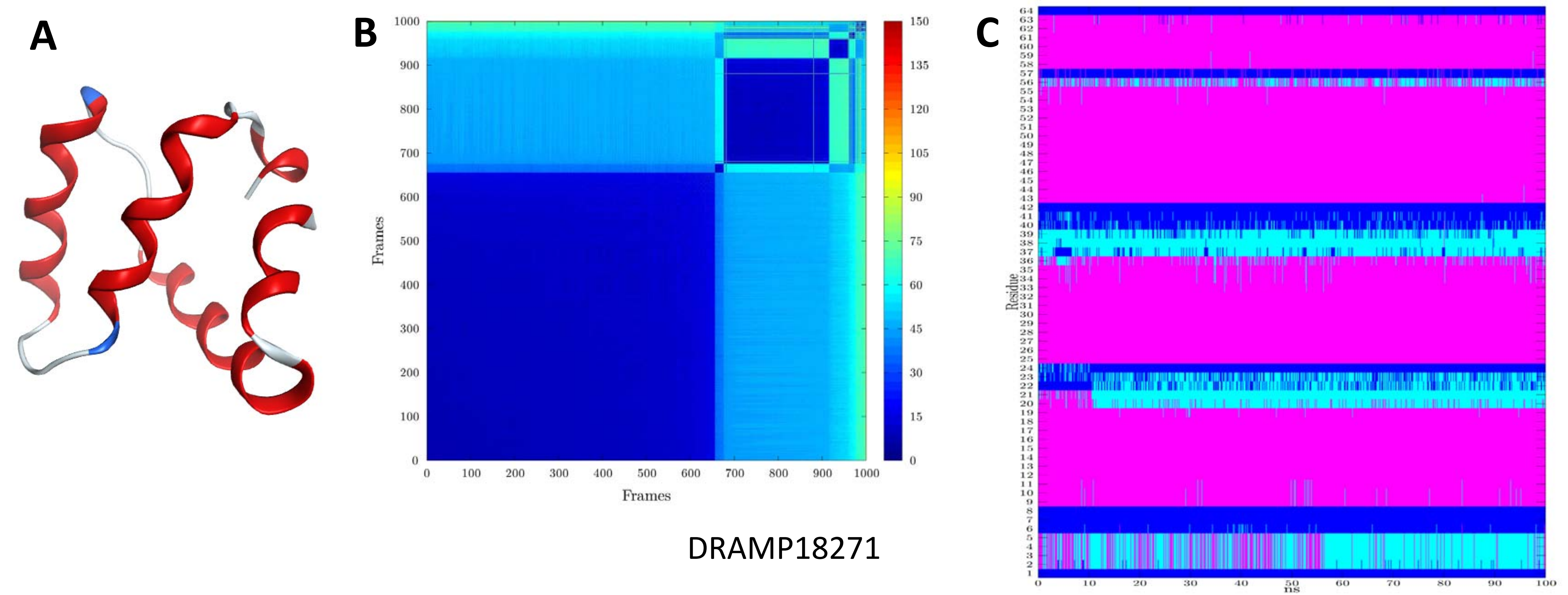
A
B
C
Frames
Frames
Residue
ns
DRAMP18271

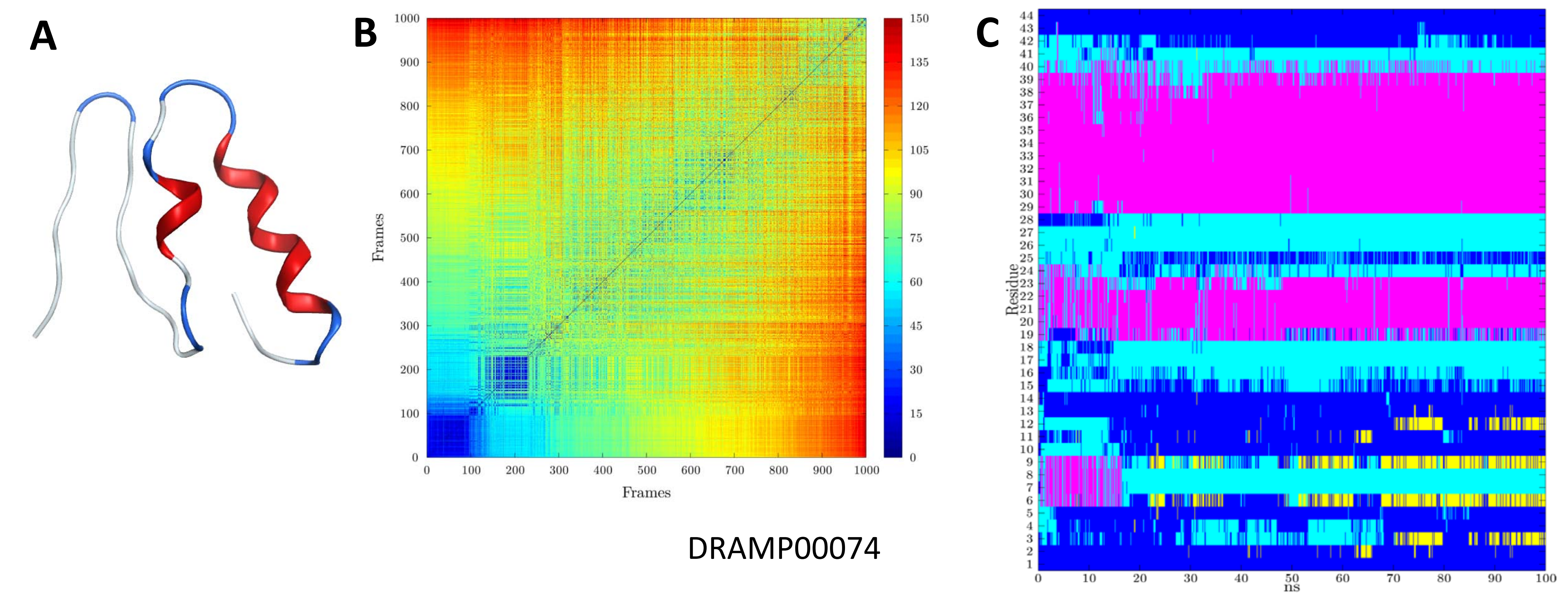

A
B
C
Frames
Frames
Residue
ns
DRAMP00074

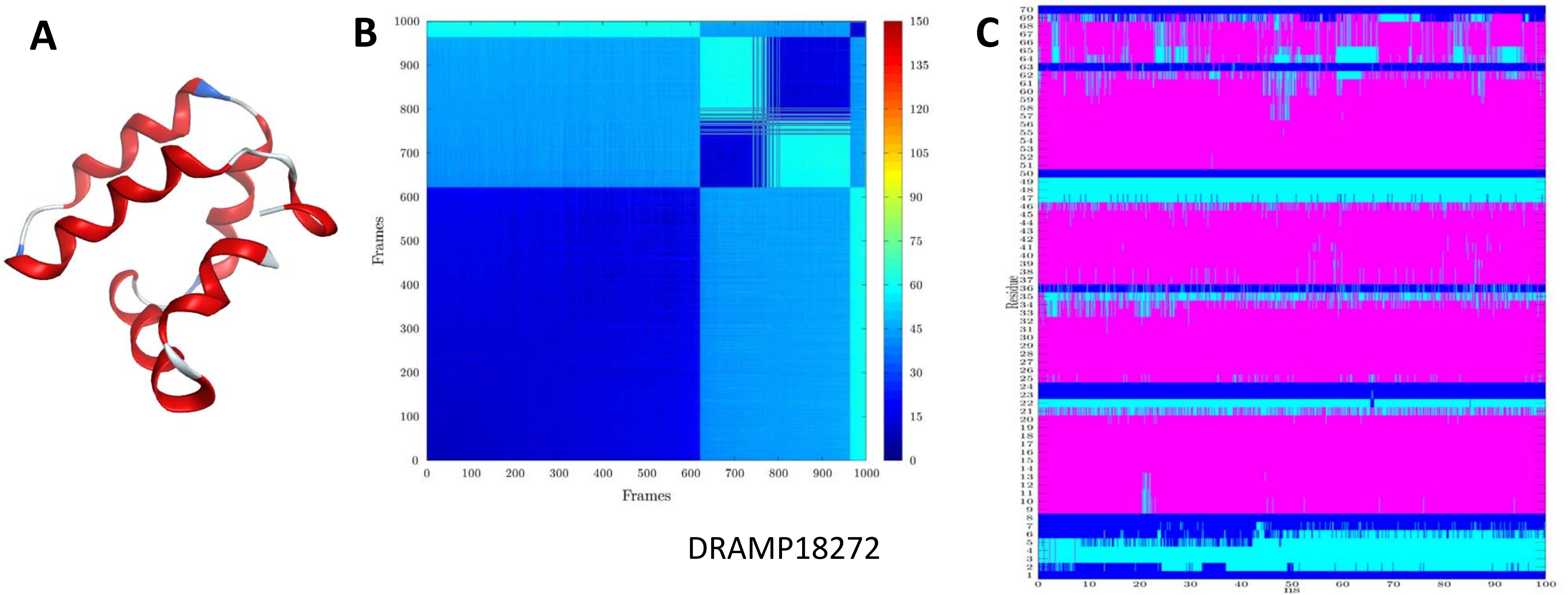
A
B
C
Frames
Frames
Residue
ns
DRAMP18272

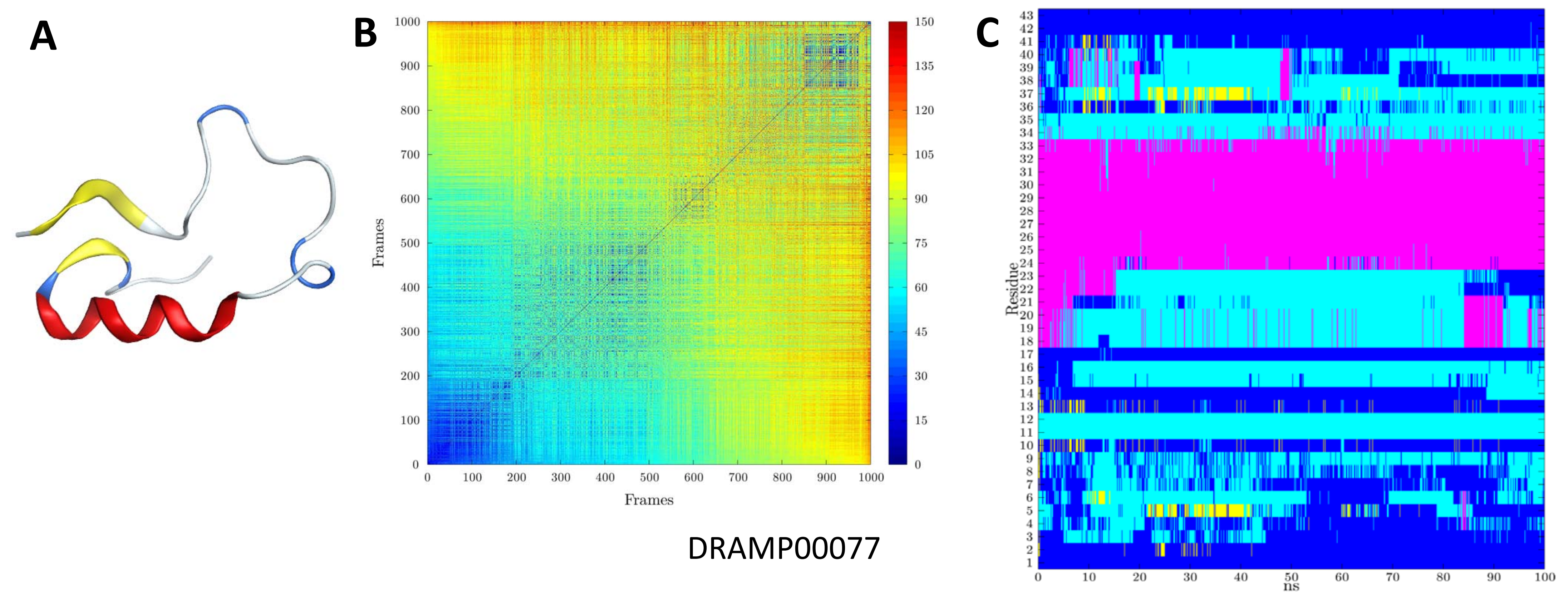
A
B
C
Frames
Frames
Residue
ns
DRAMP00077

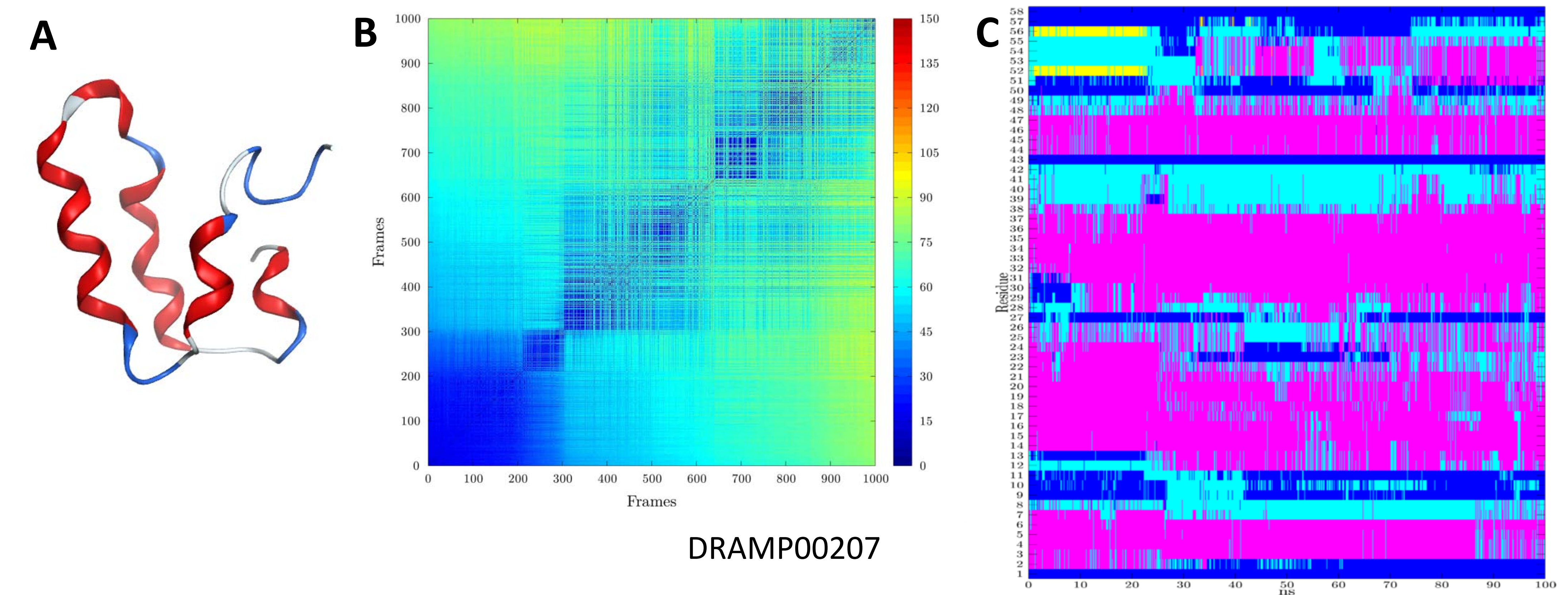

A
B
C
Frames
Frames
Residue
ns
DRAMP00207

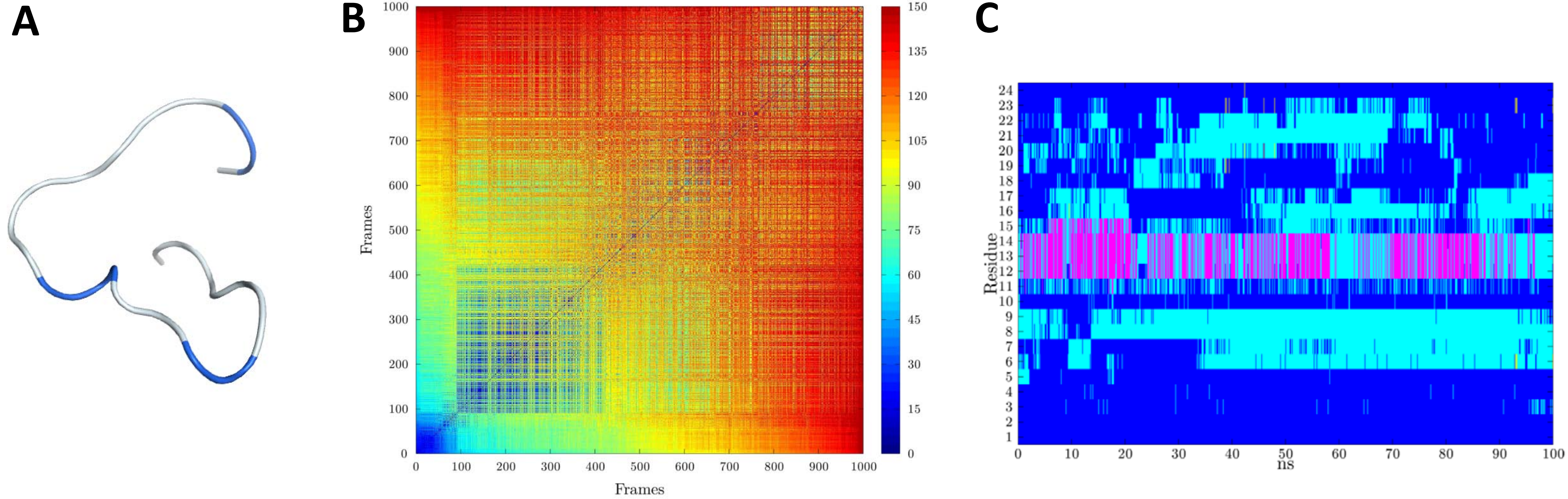

DRAMP00186

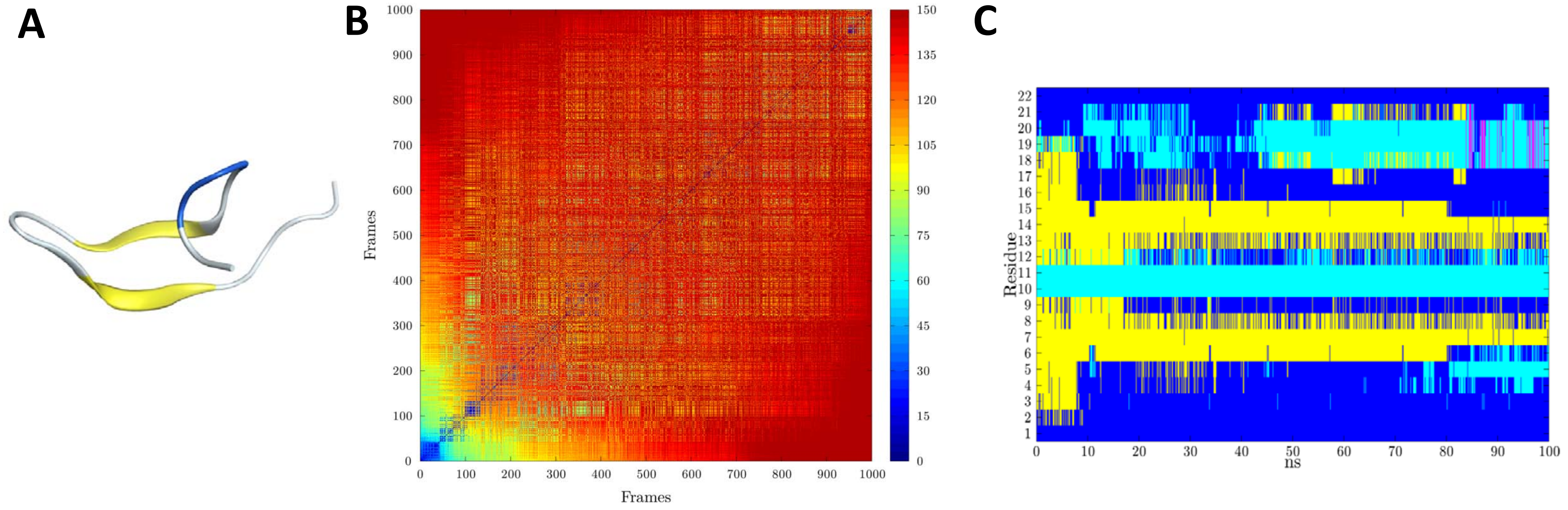
A
B
C
Frames
Frames
0
100
200
300
400
500
600
700
800
900
1000
150
135
120
105
90
75
60
45
30
15
0
Residue
ns
1
2
3
4
5
6
7
8
9
10
11
12
13
14
15
16
17
18
19
20
21
22
0
10
20
30
40
50
60
70
80
90
100

DRAMP00187

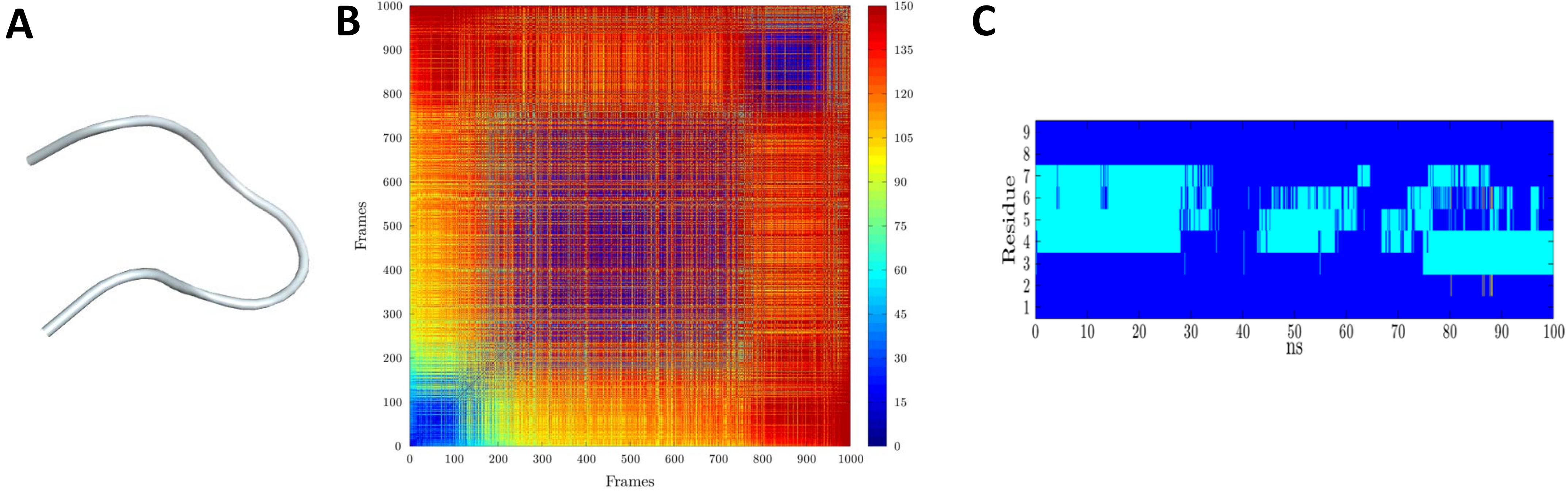

DRAMP00226

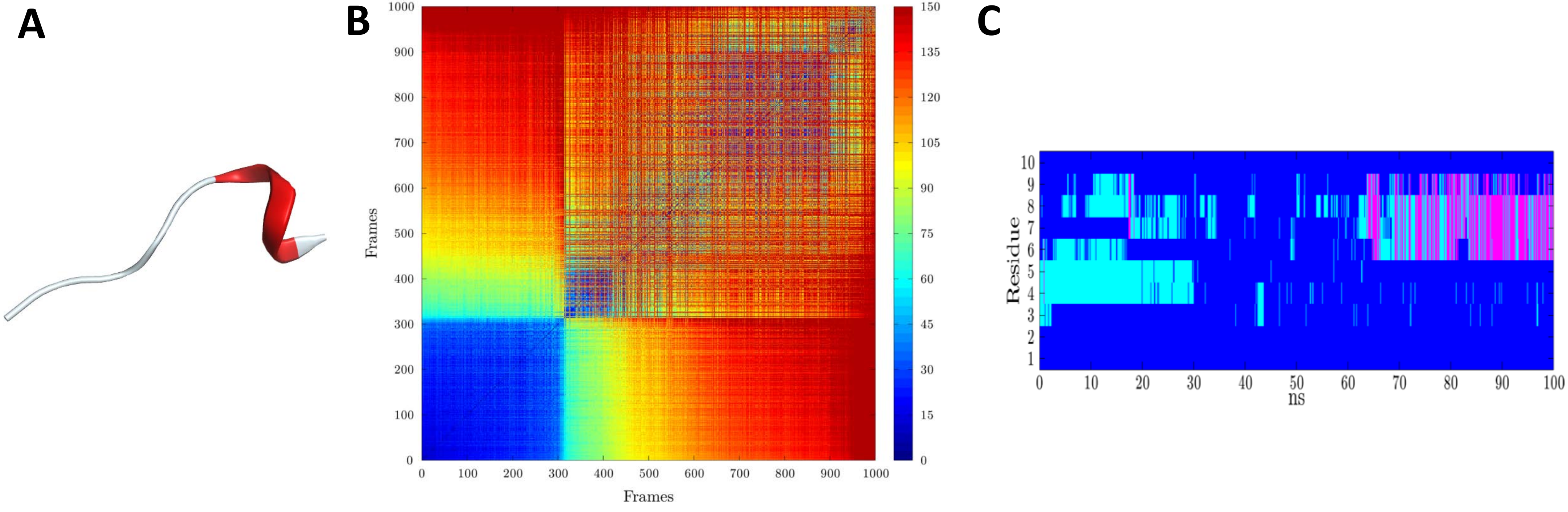
A
B
C
Frames
Frames
0
100
200
300
400
500
600
700
800
900
1000
150
135
120
105
90
75
60
45
30
15
0
Residue
ns
1
2
3
4
5
6
7
8
9
10
0
10
20
30
40
50
60
70
80
90
100

DRAMP00227

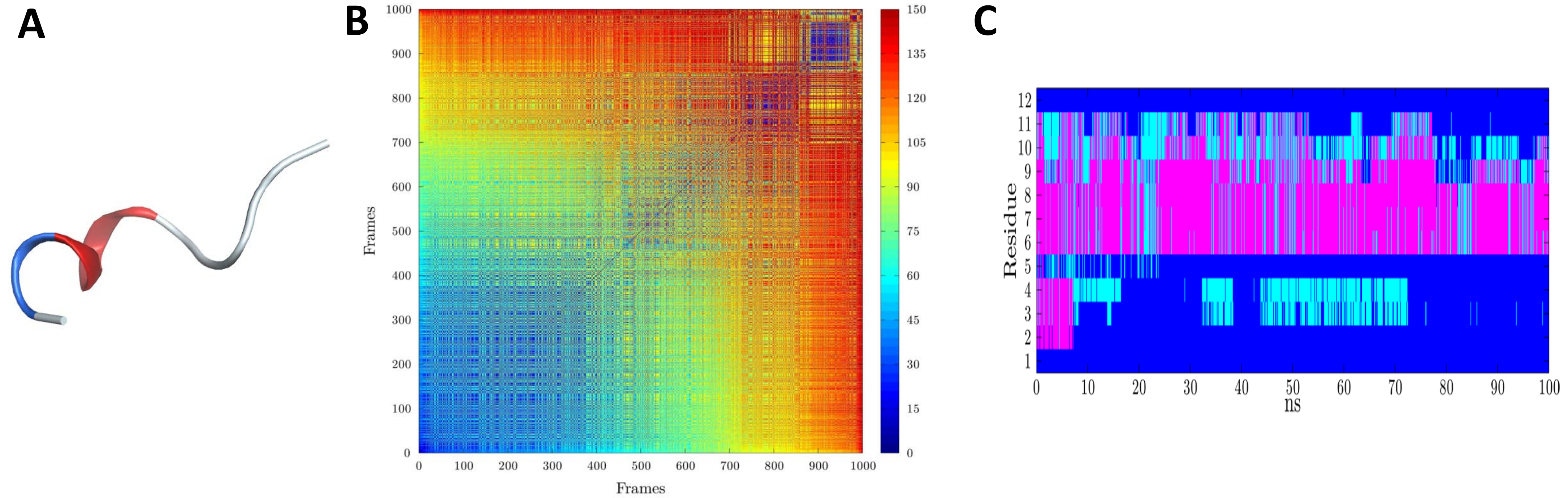

DRAMP00228

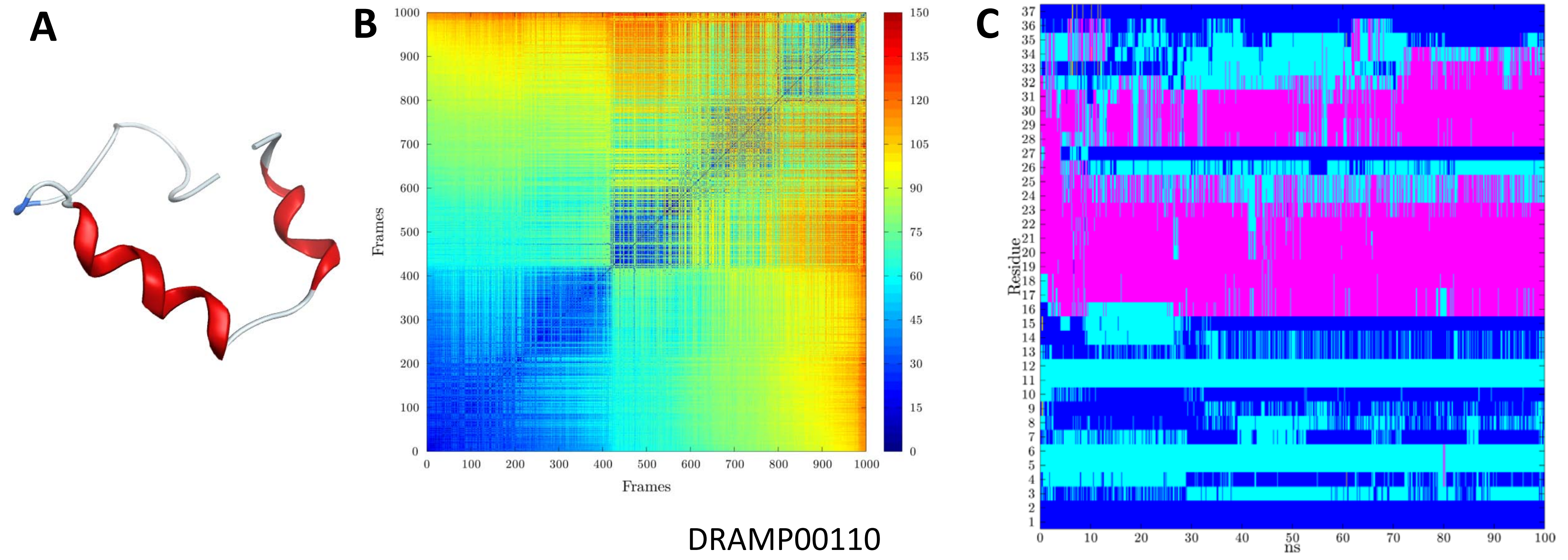

DRAMP00110

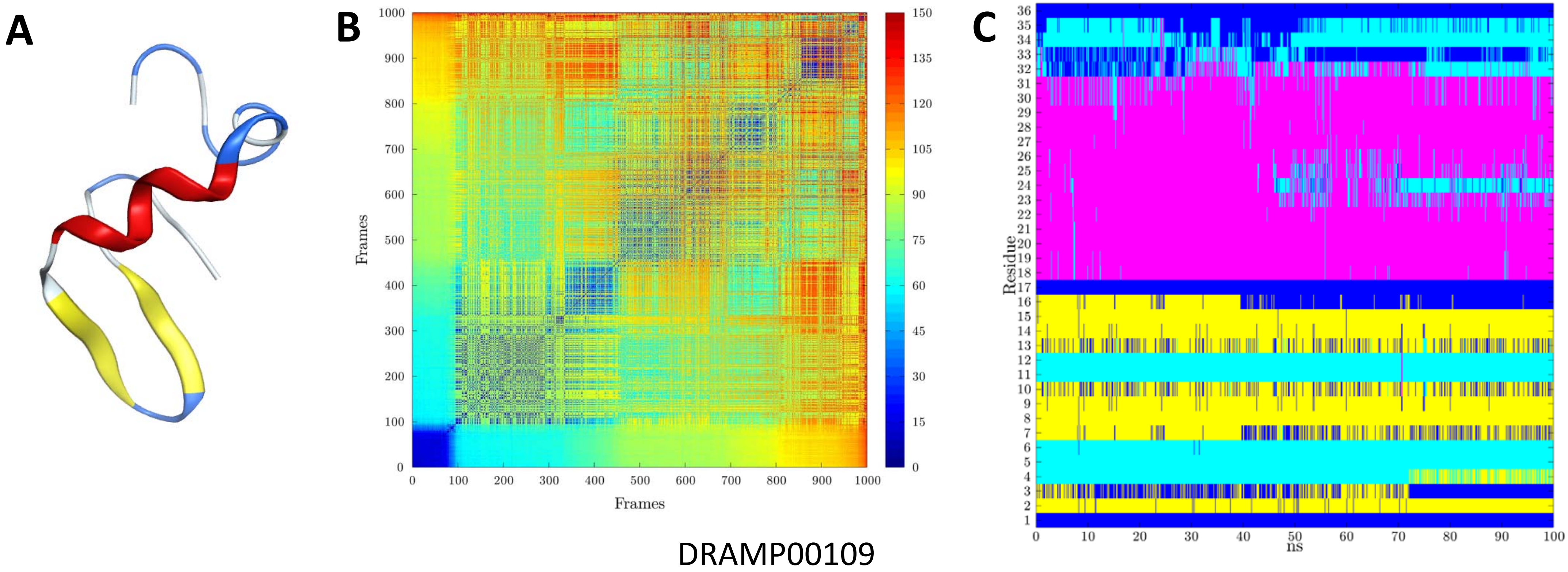
A
B
C
Frames
Frames
Residue
ns
DRAMP00109

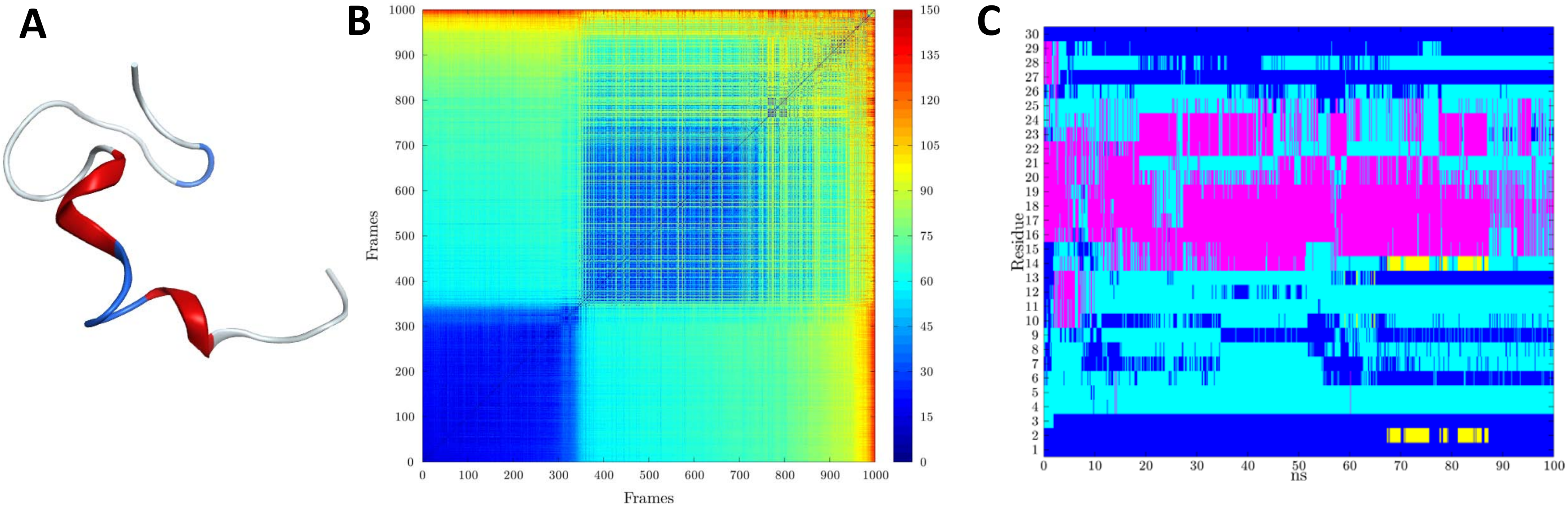
A
B
C
Frames
Frames
0
100
200
300
400
500
600
700
800
900
1000
0
15
30
45
60
75
90
105
120
135
150
Residue
ns
0
10
20
30
40
50
60
70
80
90
100

DRAMP18281

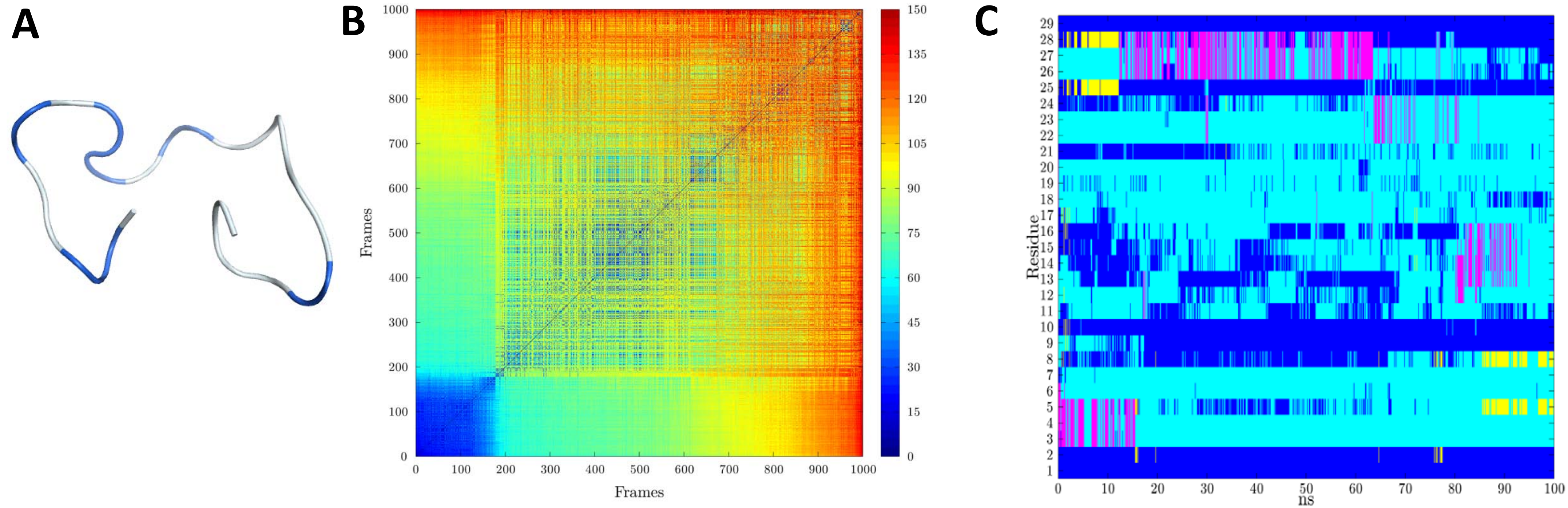

DRAMP00010

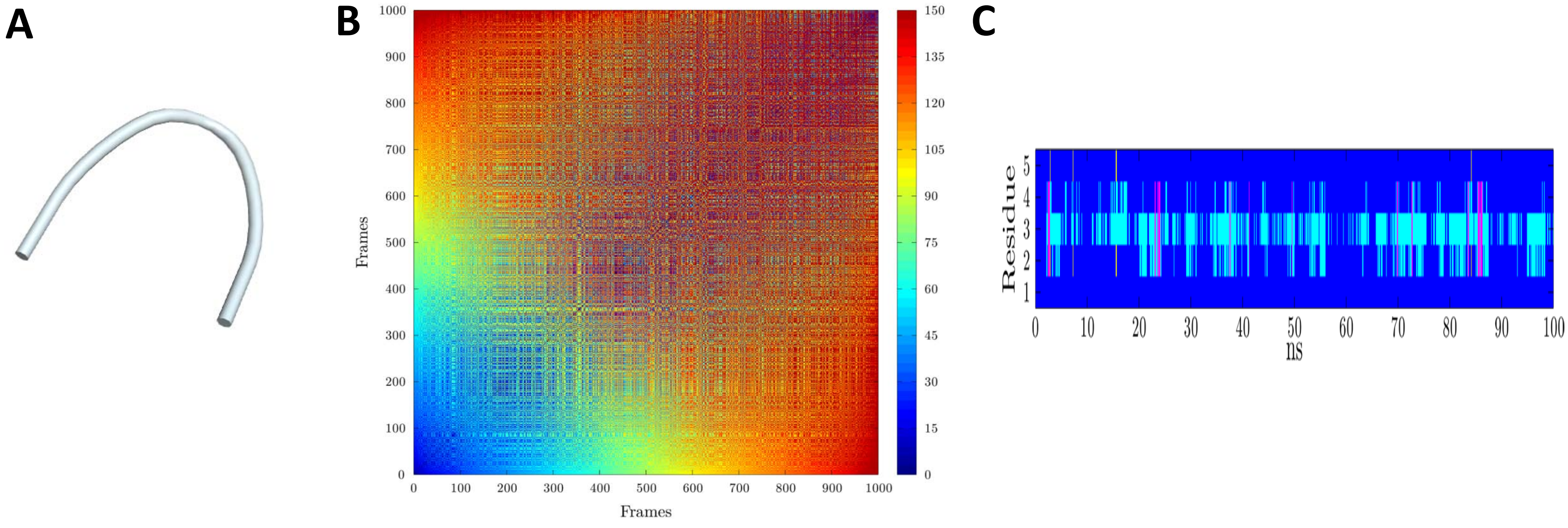
A
B
C
Frames
Frames
Residue
ns
0
100
200
300
400
500
600
700
800
900
1000
0
15
30
45
60
75
90
105
120
135
150
0
10
20
30
40
50
60
70
80
90
100
1
2
3
4
5

DRAMP18282

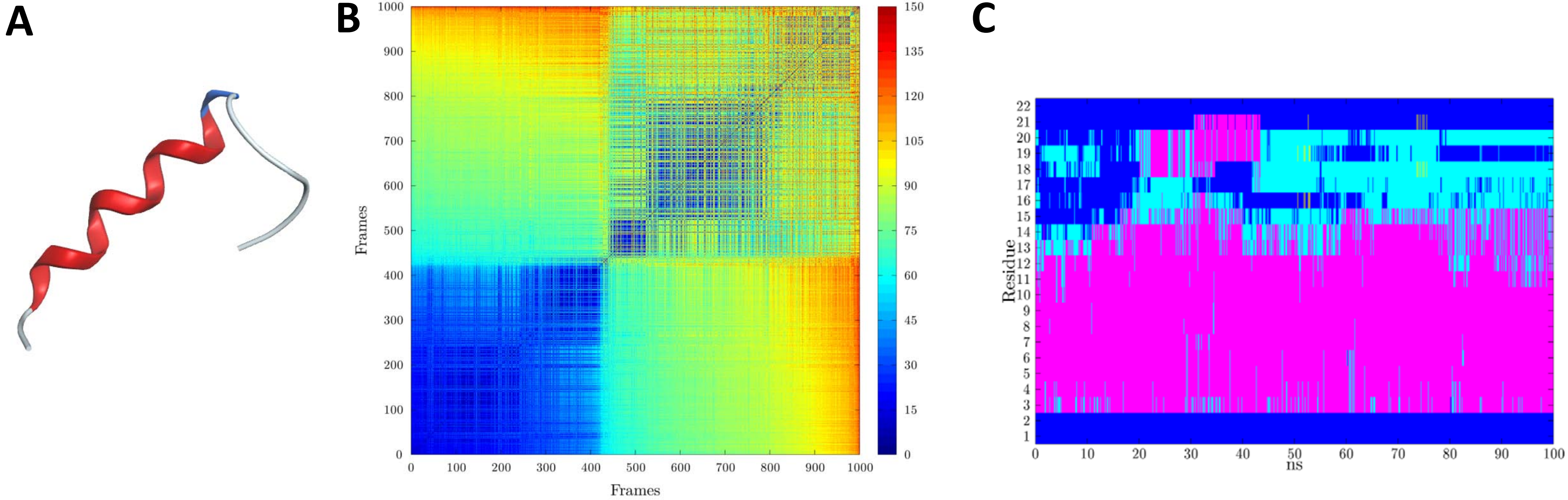

DRAMP18283

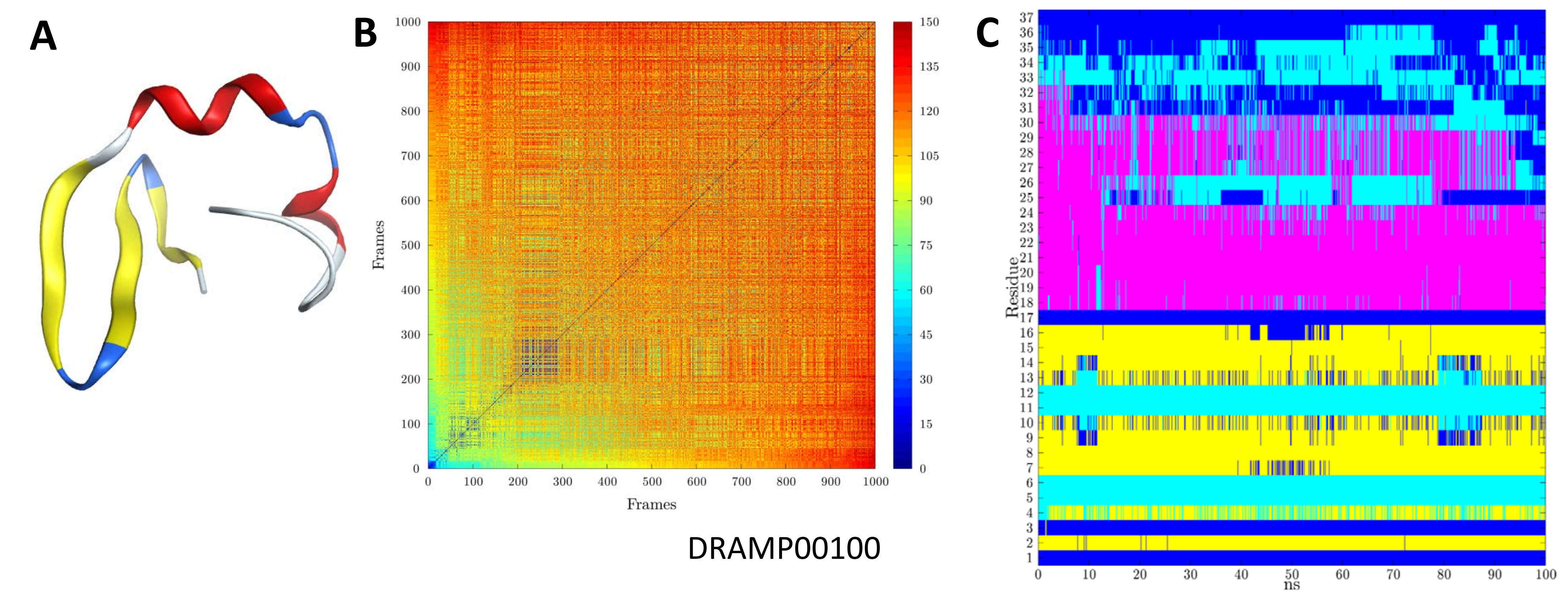
A
B
C
Frames
0 100 200 300 400 500 600 700 800 900 1000
Frames
0 100 200 300 400 500 600 700 800 900 1000
150 135 120 105 90 75 60 45 30 15 0
Residue
1 2 3 4 5 6 7 8 9 10 11 12 13 14 15 16 17 18 19 20 21 22 23 24 25 26 27 28 29 30 31 32 33 34 35 36 37
ns
0 10 20 30 40 50 60 70 80 90 100
DRAMP00100

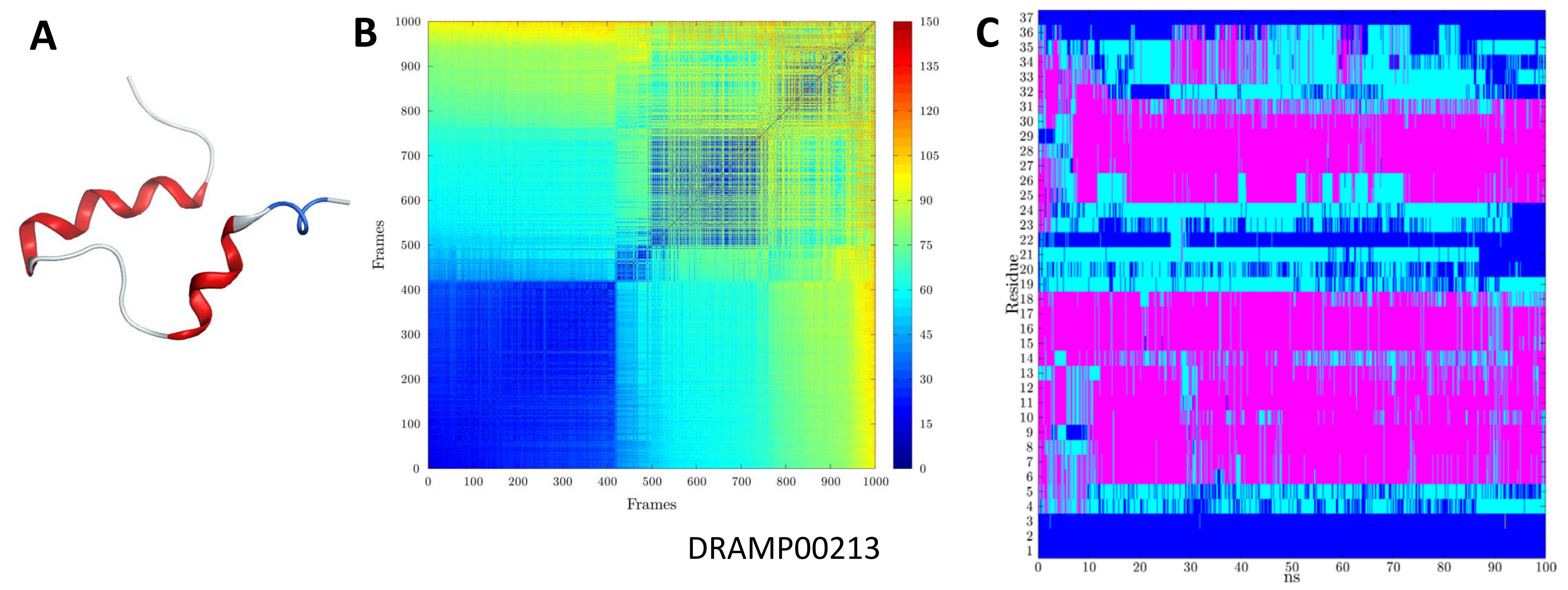

A
B
C
Frames
Frames
Residue
ns
DRAMP00213

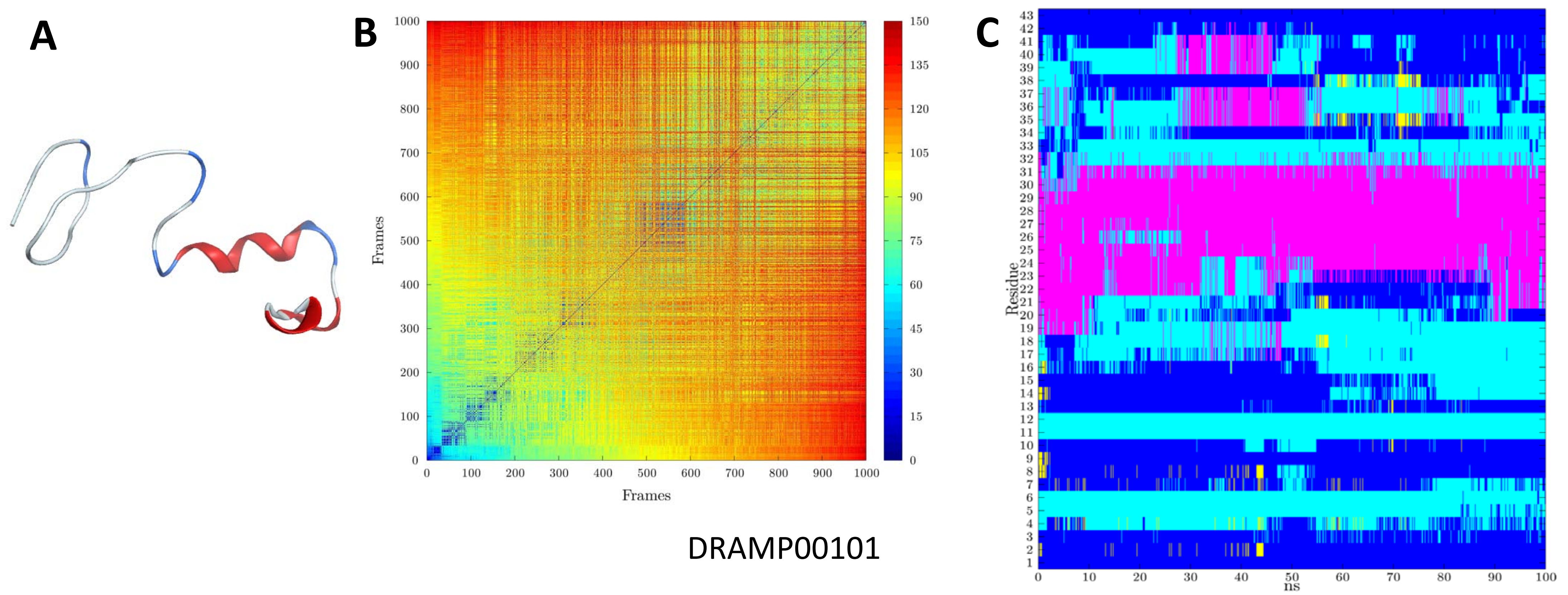
A
B
C
Frames
Frames
Residue
ns
DRAMP00101

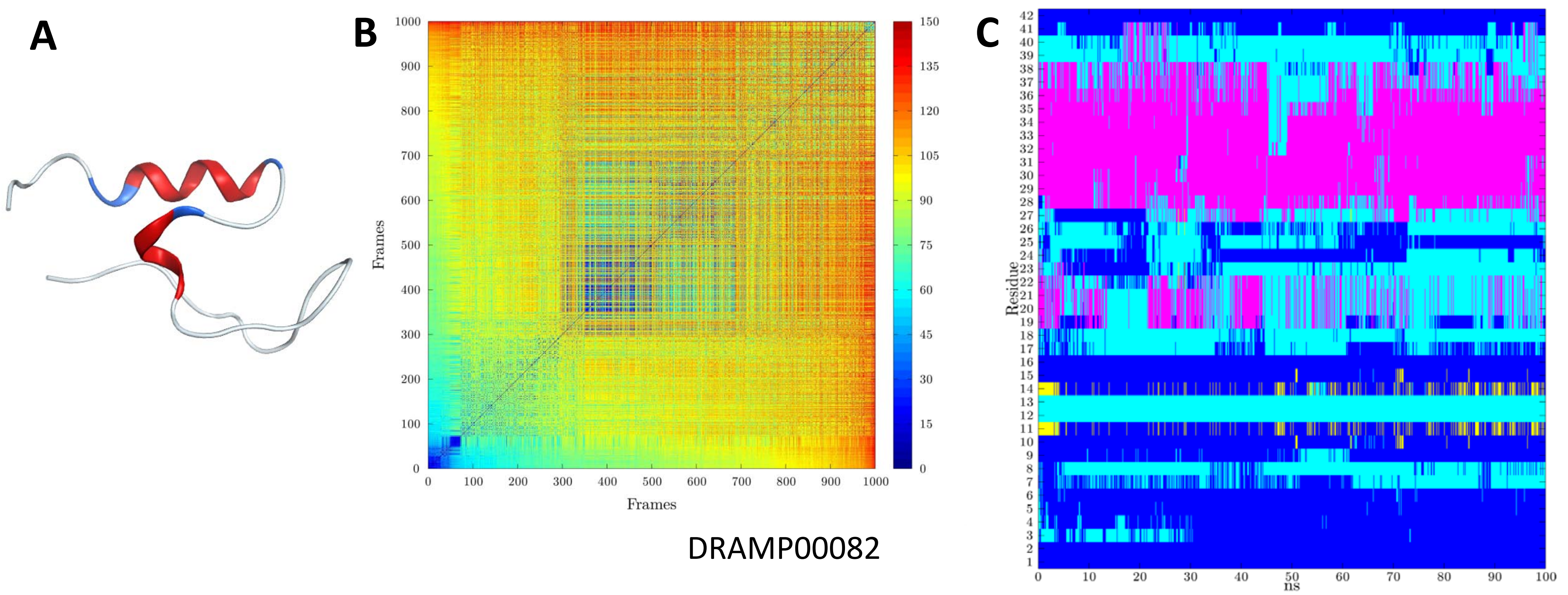
A
B
C
Frames
Frames
Residue
ns
DRAMP00082

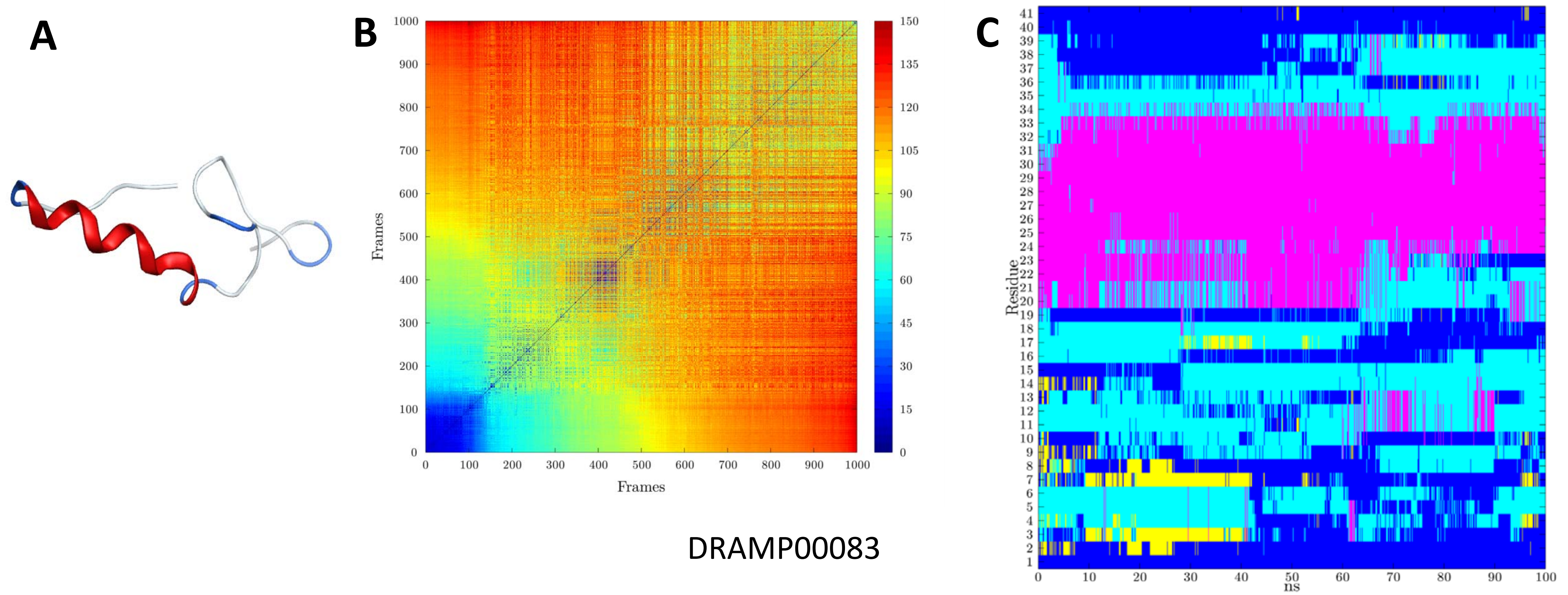
A
B
C
Frames
Frames
Residue
ns
DRAMP00083

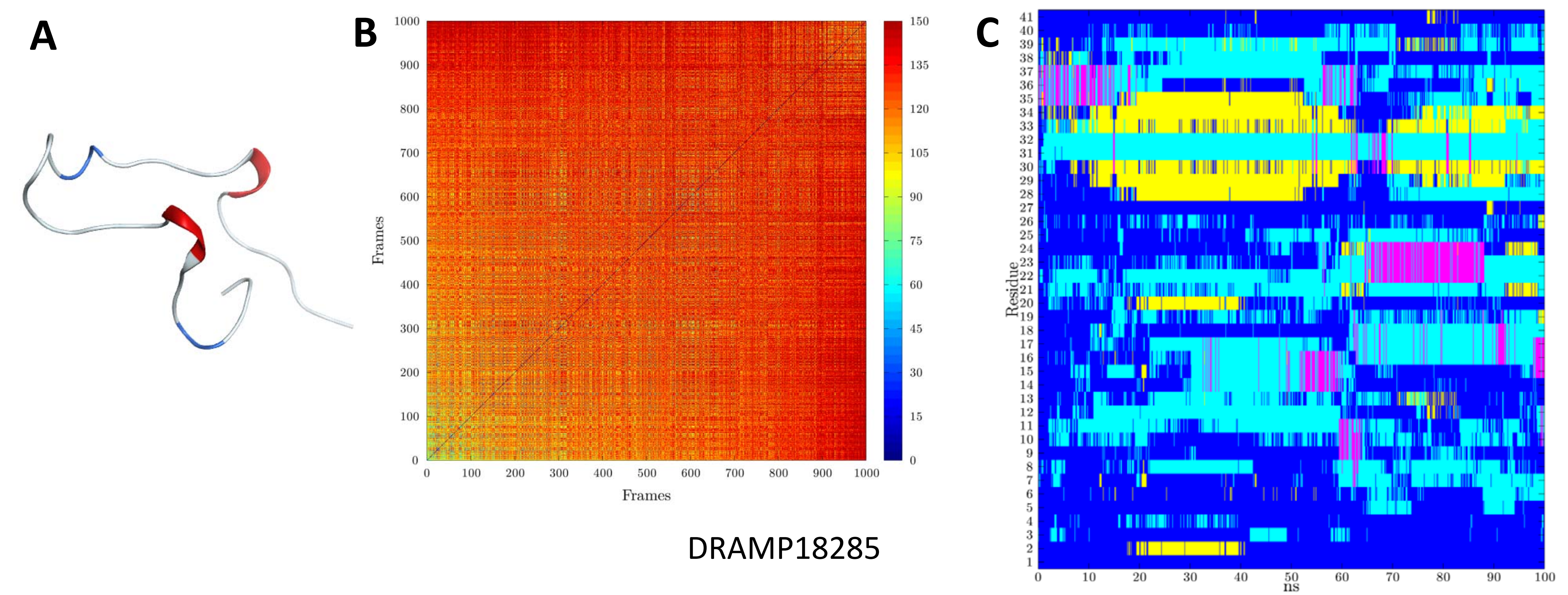
A
B
Frames
Frames
C
Residue
ns
DRAMP18285

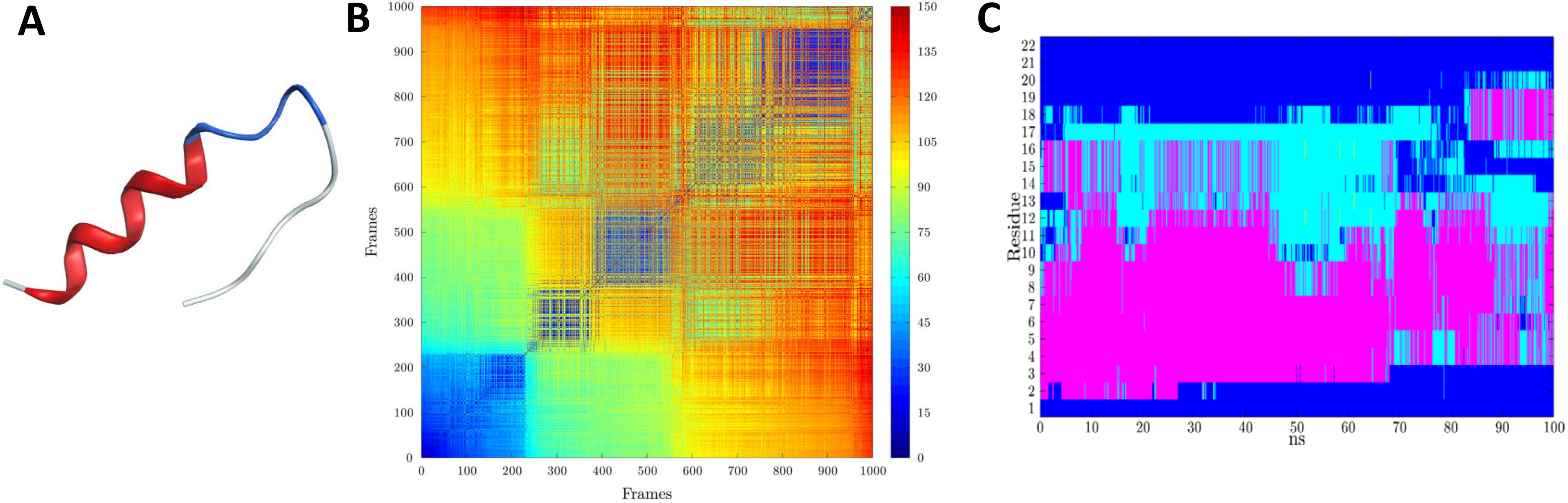

DRAMP18289

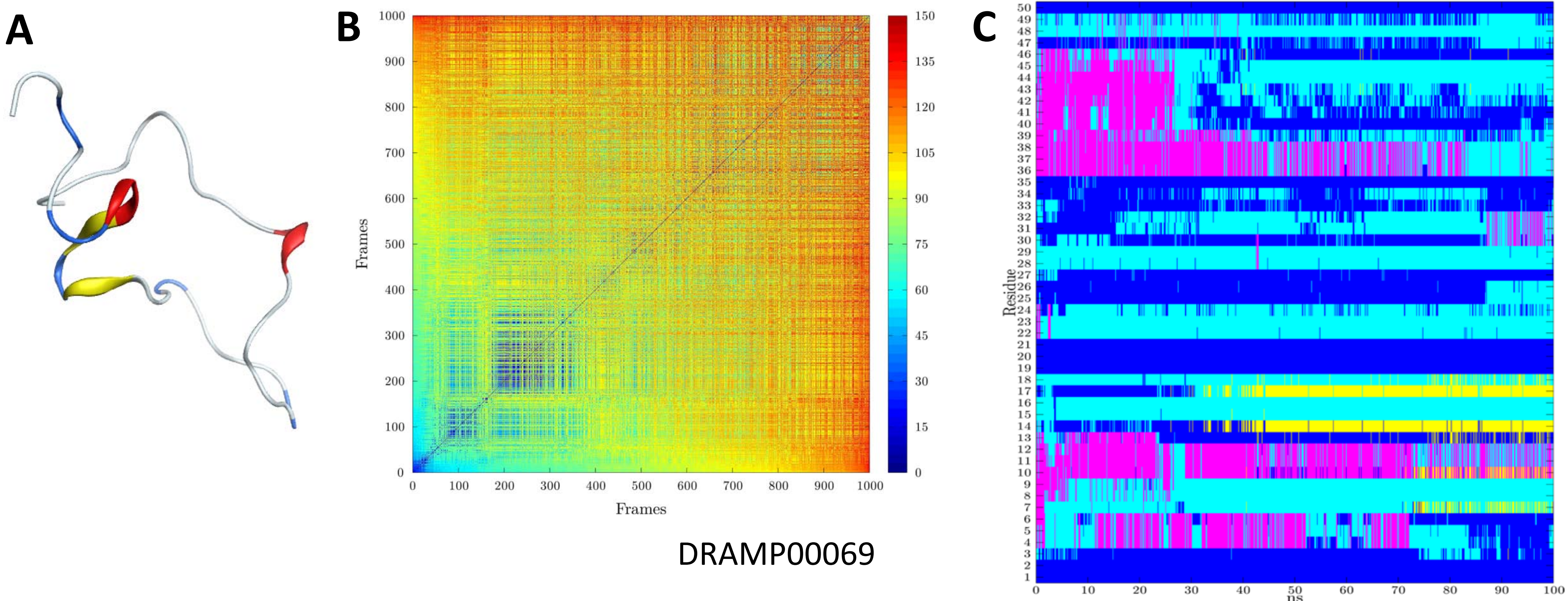
A
B
C
Frames
Frames
Residue
ns
DRAMP00069

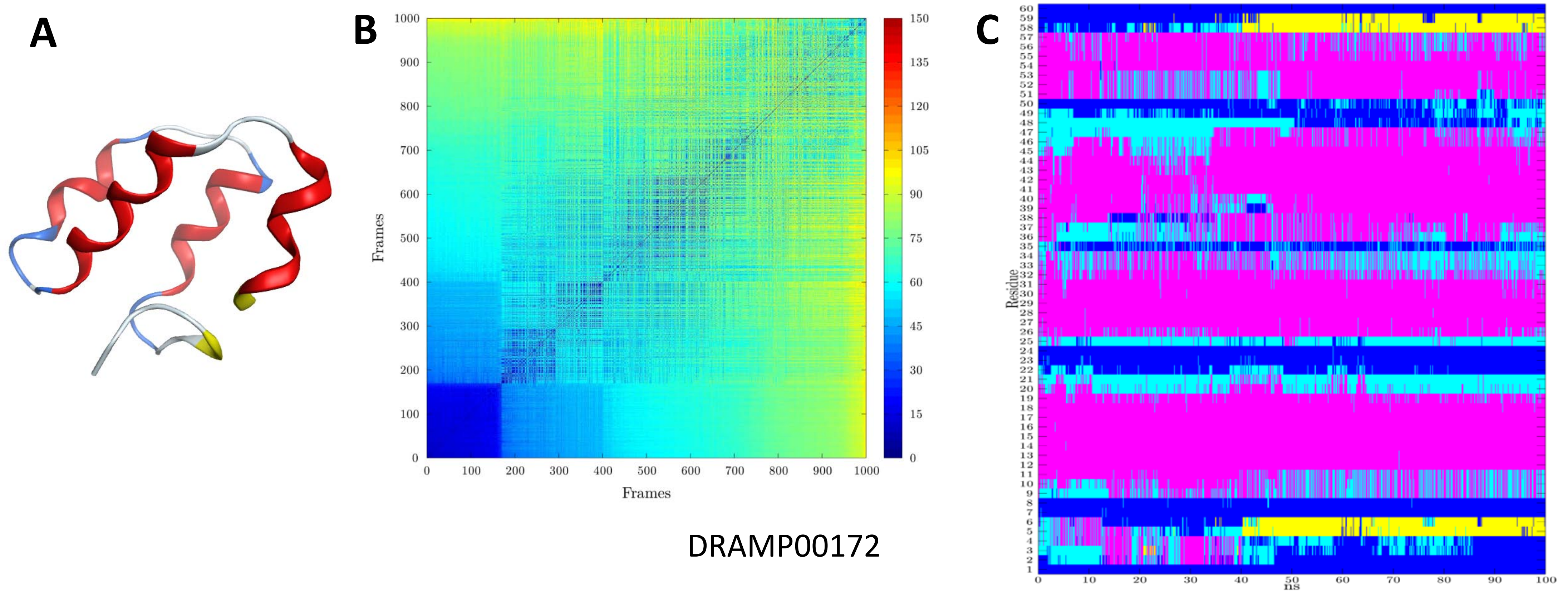
A
B
C
Frames
Frames
Residue
ns
DRAMP00172

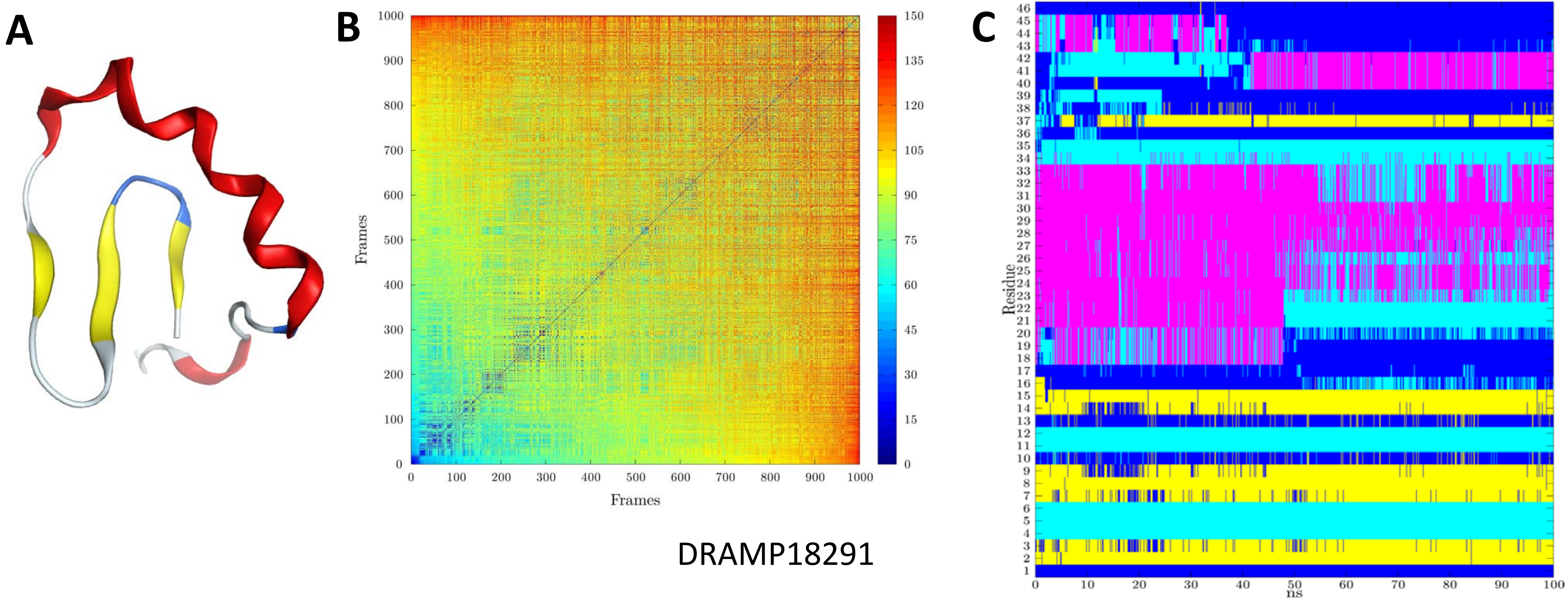
A
B
C
Frames
Frames
Residue
ns
DRAMP18291

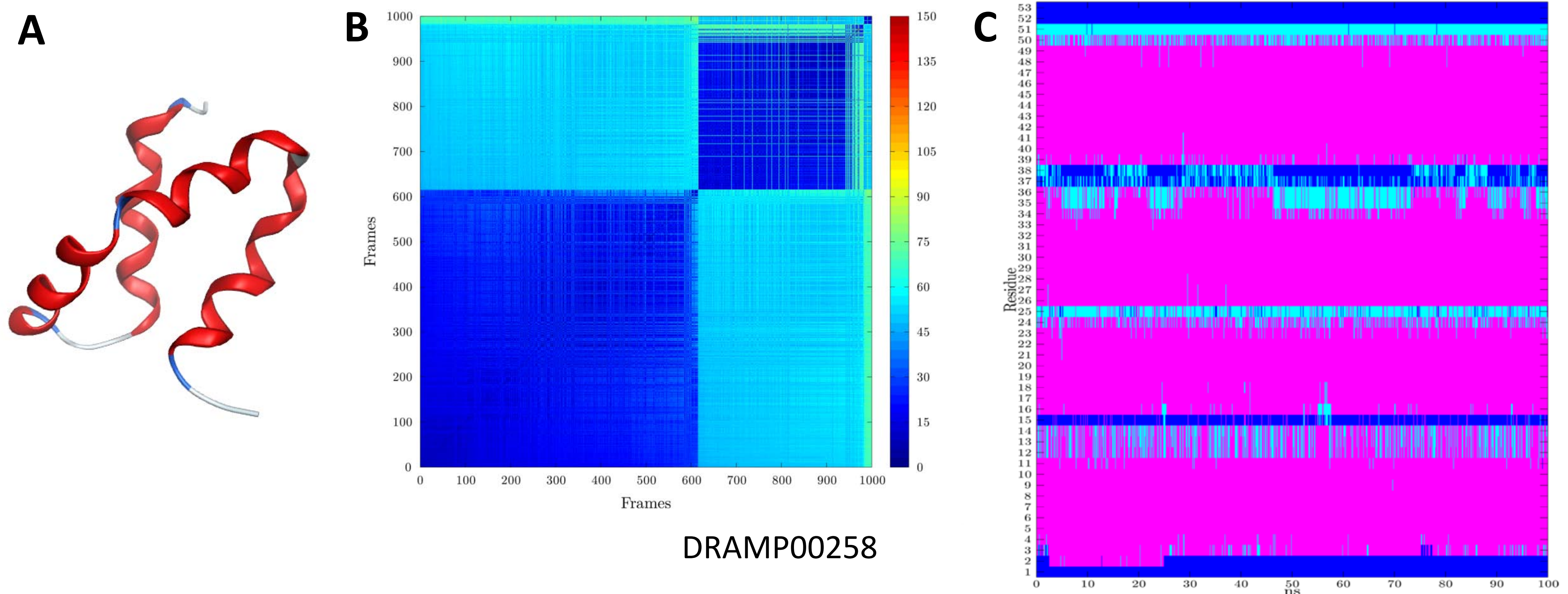

A
B
C
Frames
Frames
Residue
ns
DRAMP00258

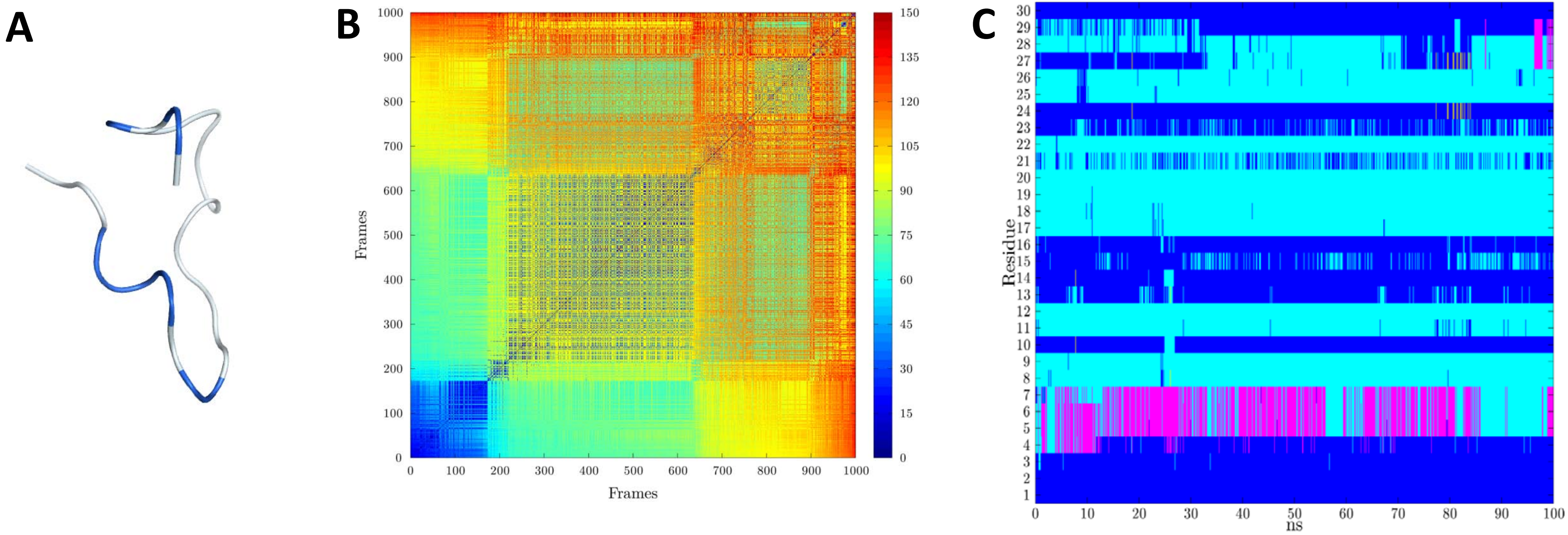
A
B
C
Frames
Frames
0
100
200
300
400
500
600
700
800
900
1000
150
135
120
105
90
75
60
45
30
15
0
Residue
ns
1
2
3
4
5
6
7
8
9
10
11
12
13
14
15
16
17
18
19
20
21
22
23
24
25
26
27
28
29
30
0
10
20
30
40
50
60
70
80
90
100

DRAMP00008

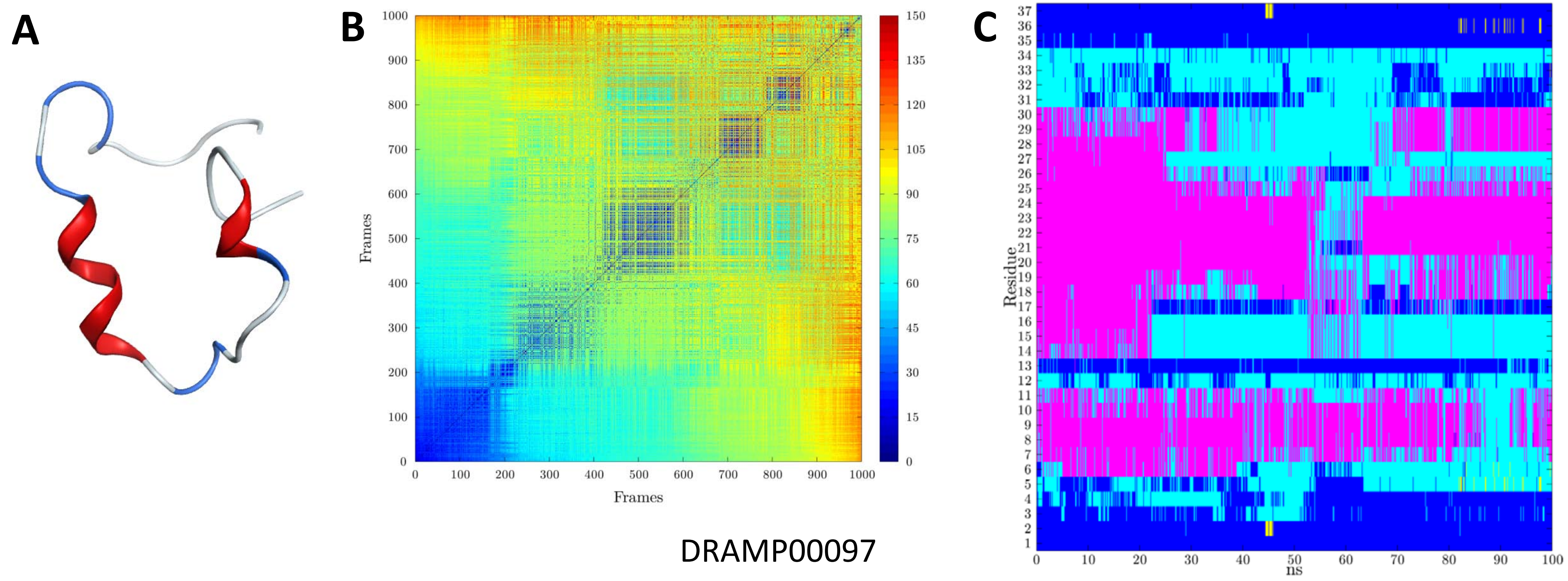

DRAMP00097

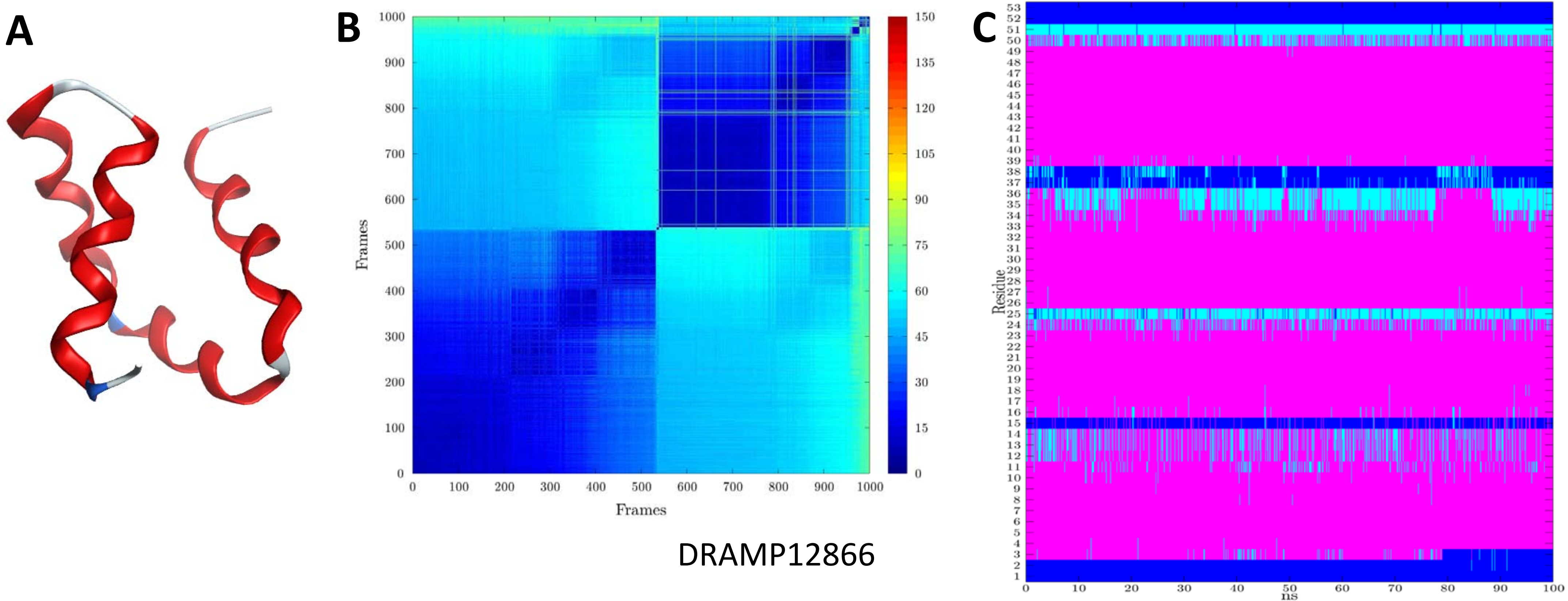

DRAMP12866

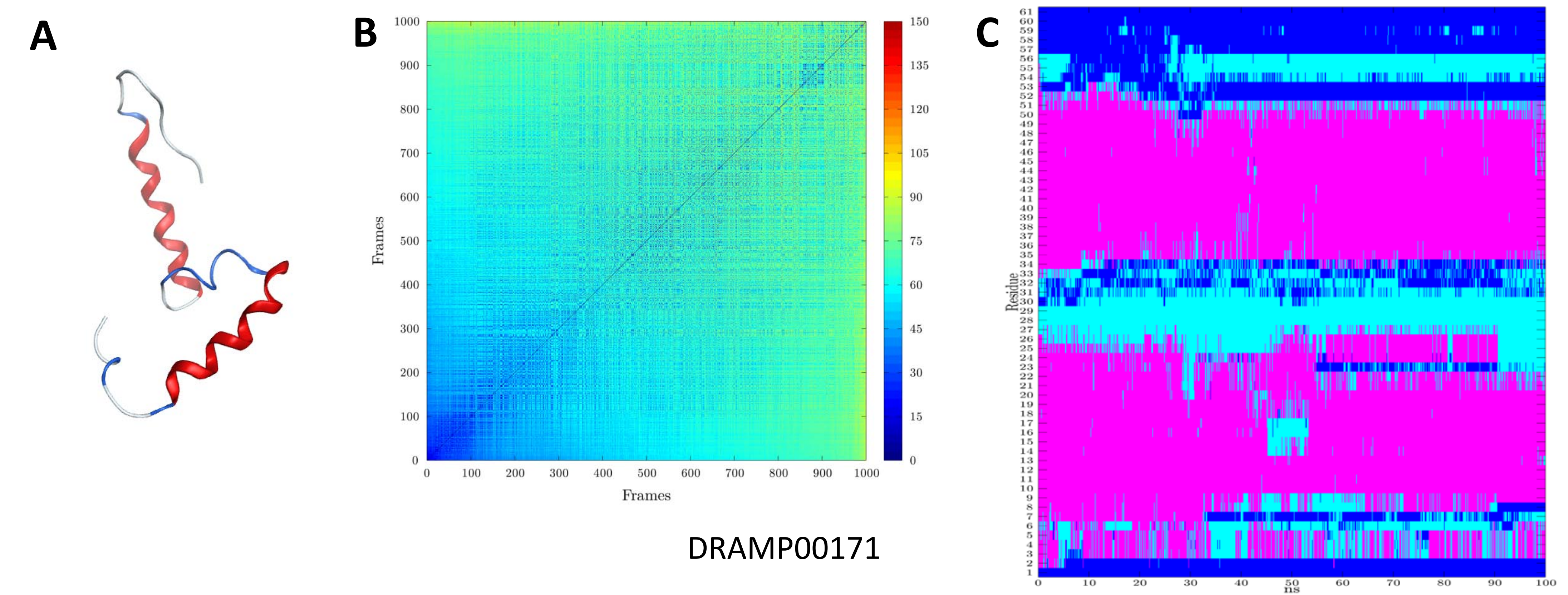

DRAMP00171

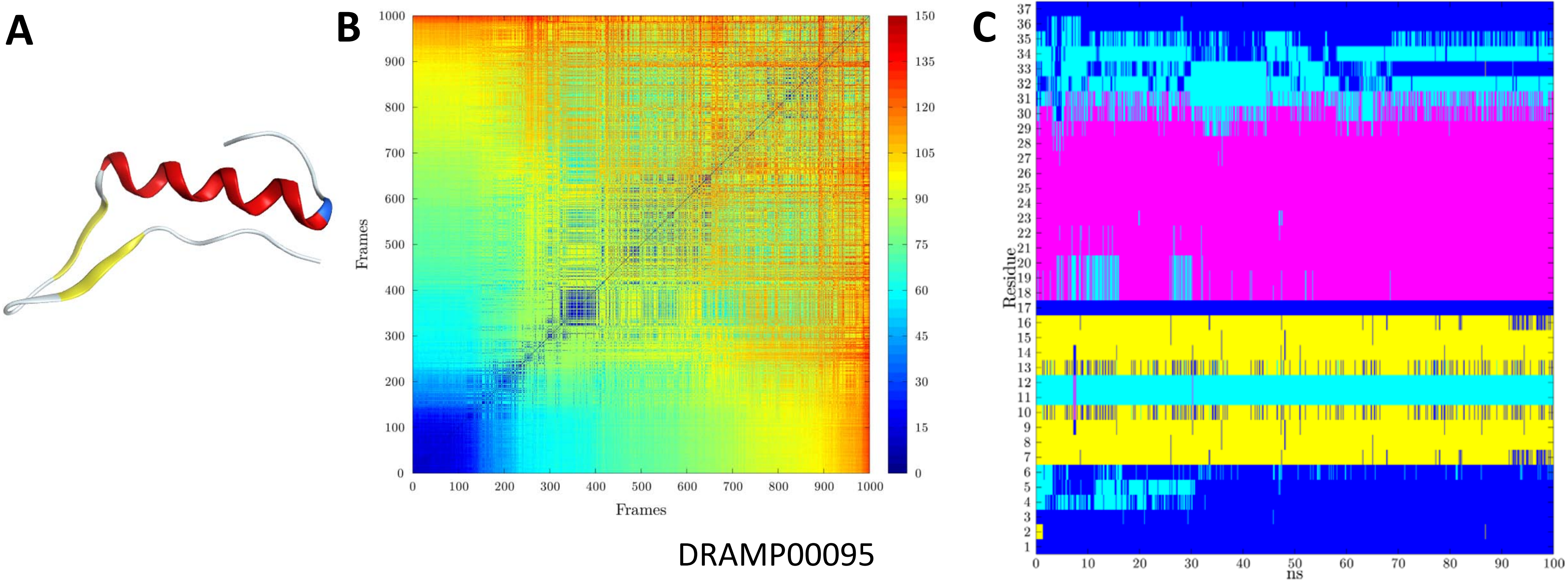

DRAMP00095

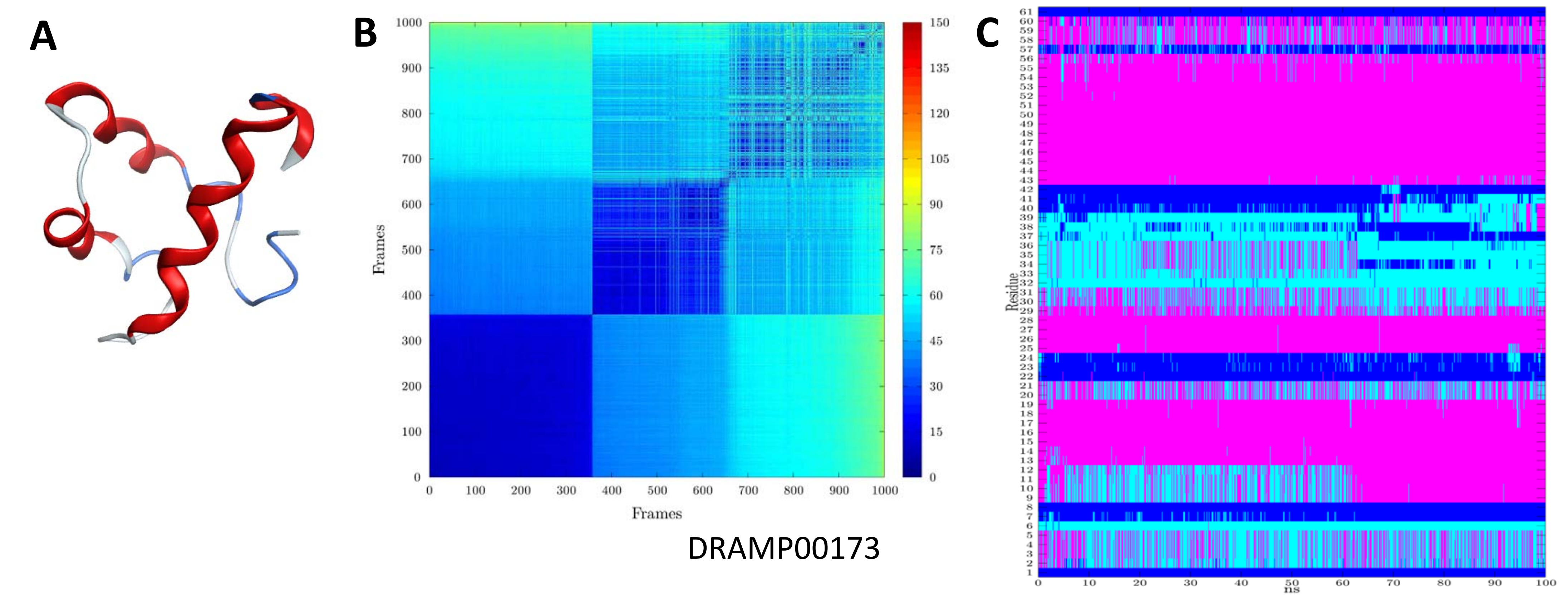
A
B
C
Frames
Frames
DRAMP00173
Residue
ns

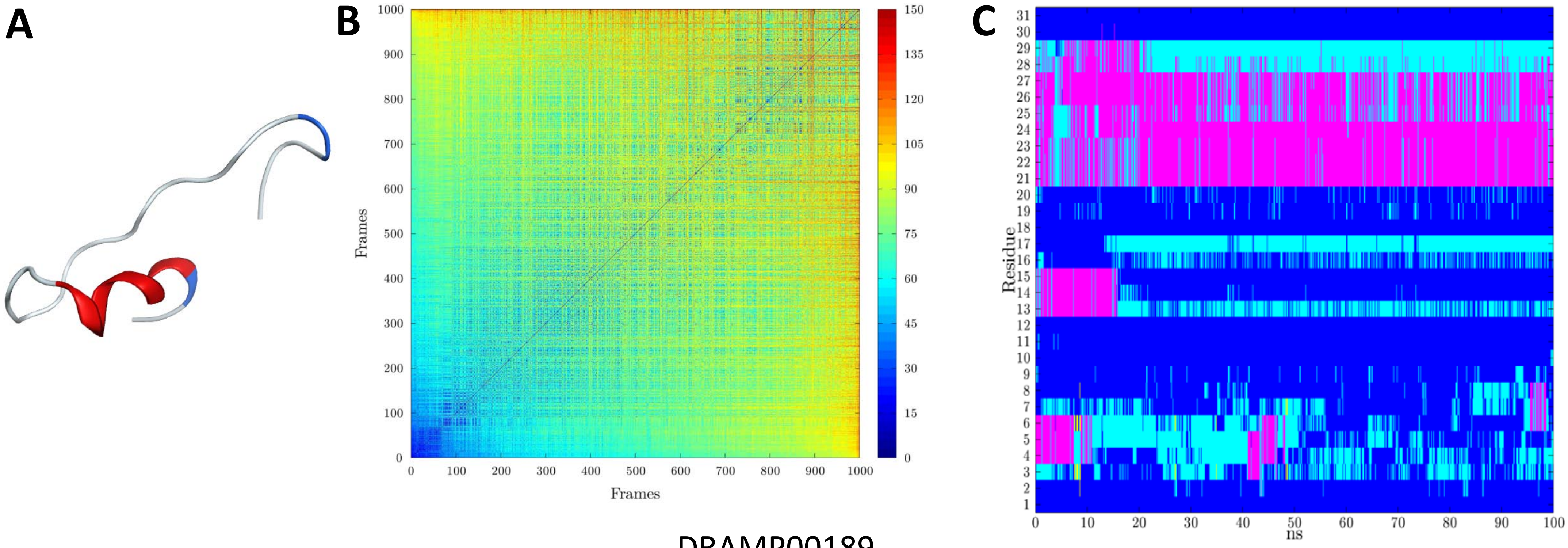

DRAMP00189

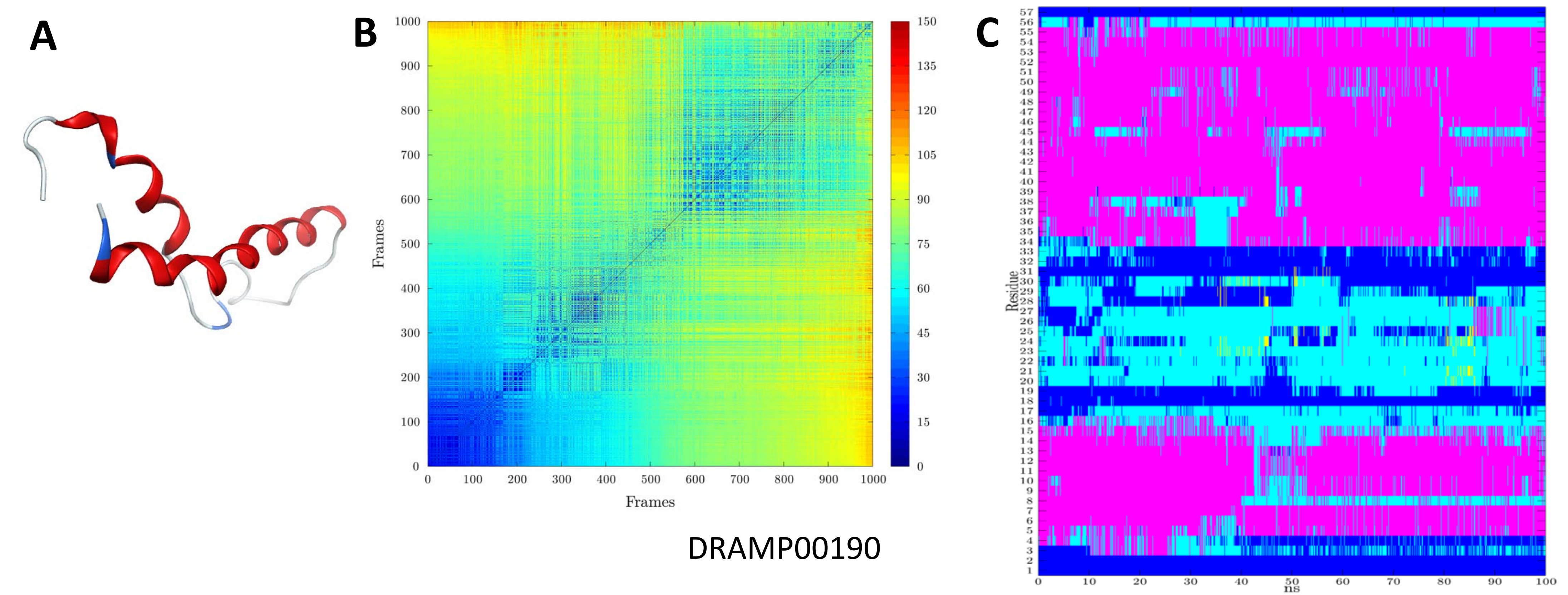
A
B
C
Frames
Frames
DRAMP00190
Residue
ns

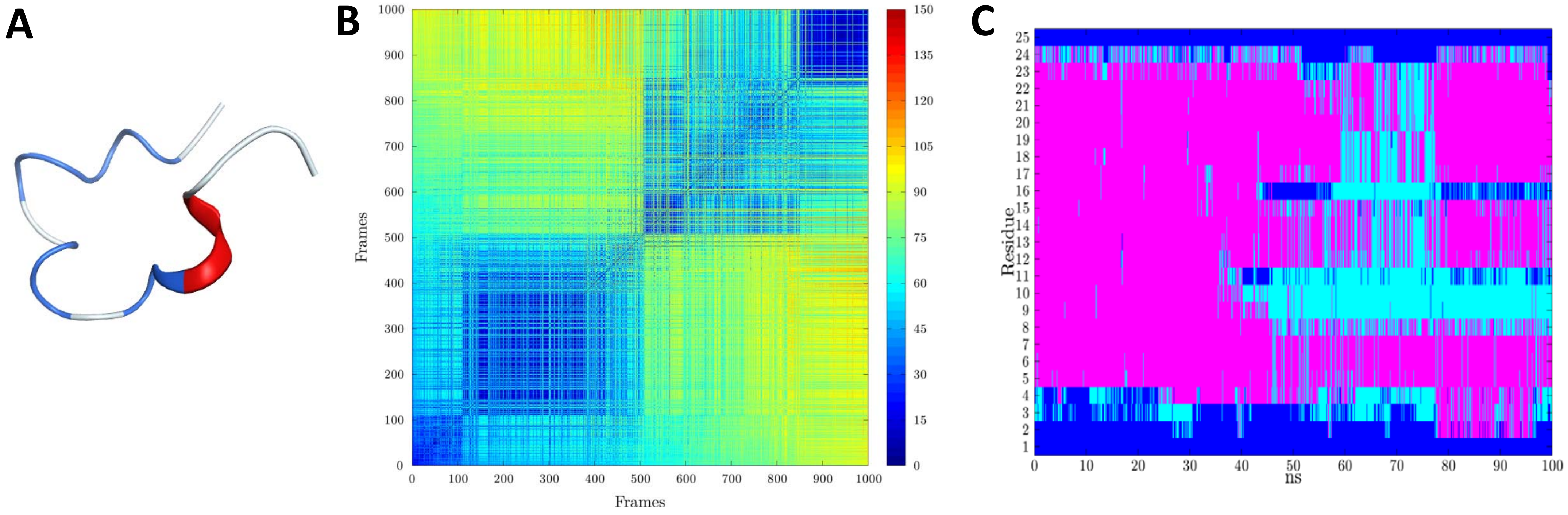

DRAMP00001

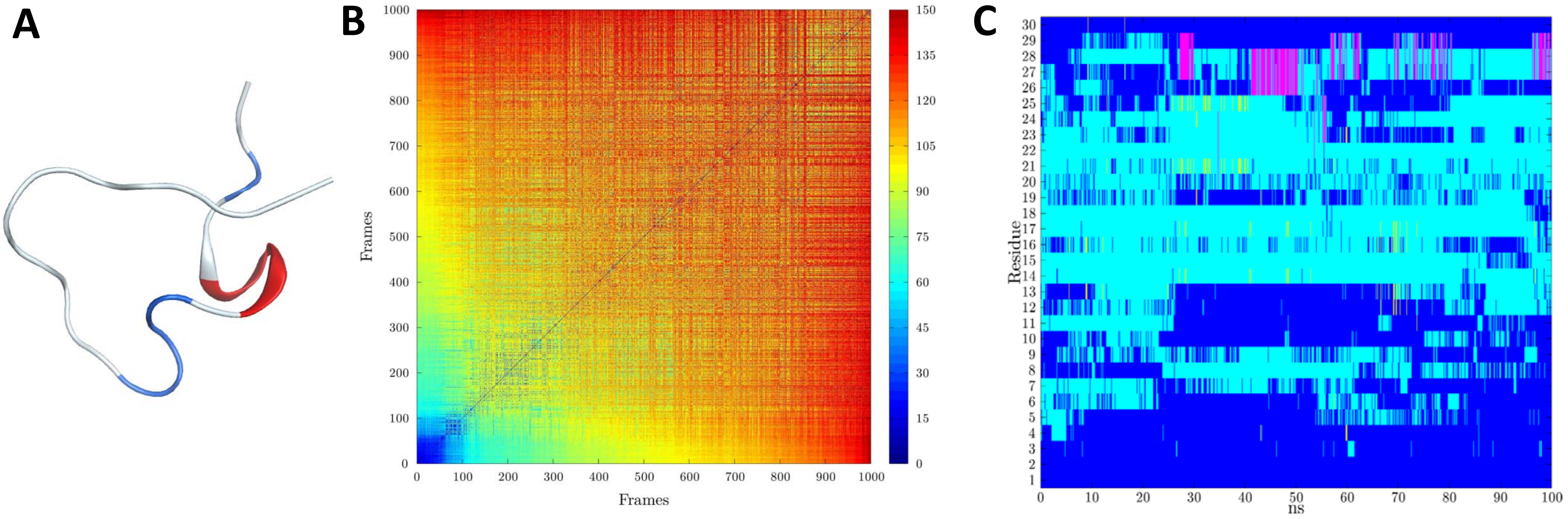

DRAMP00035

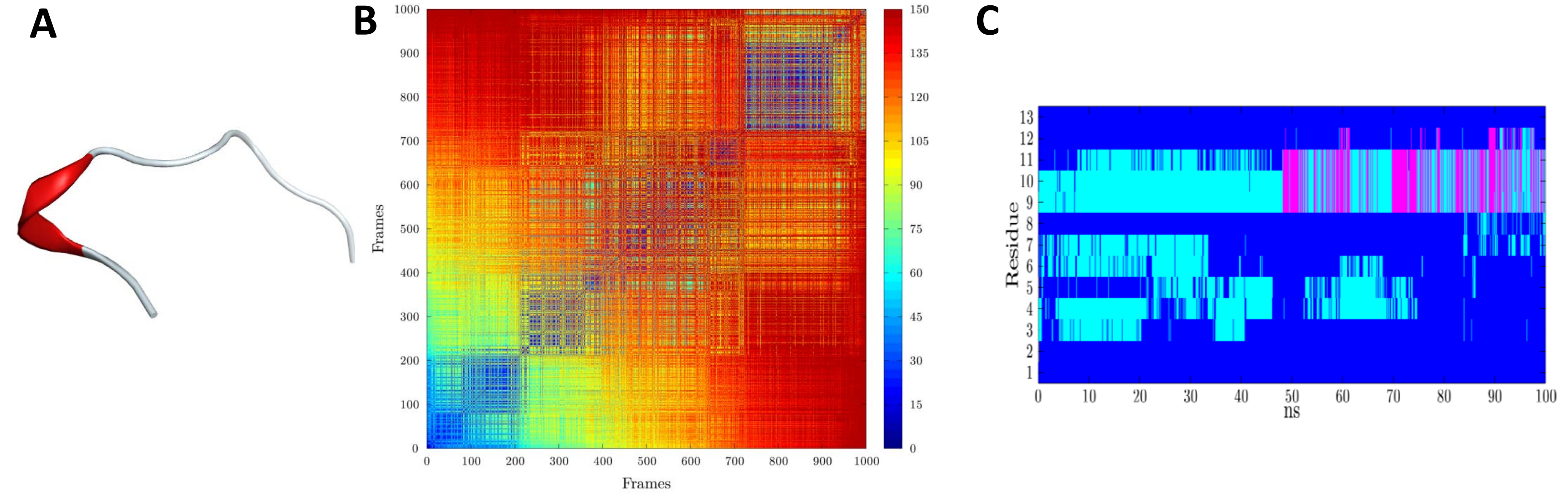

DRAMP18305

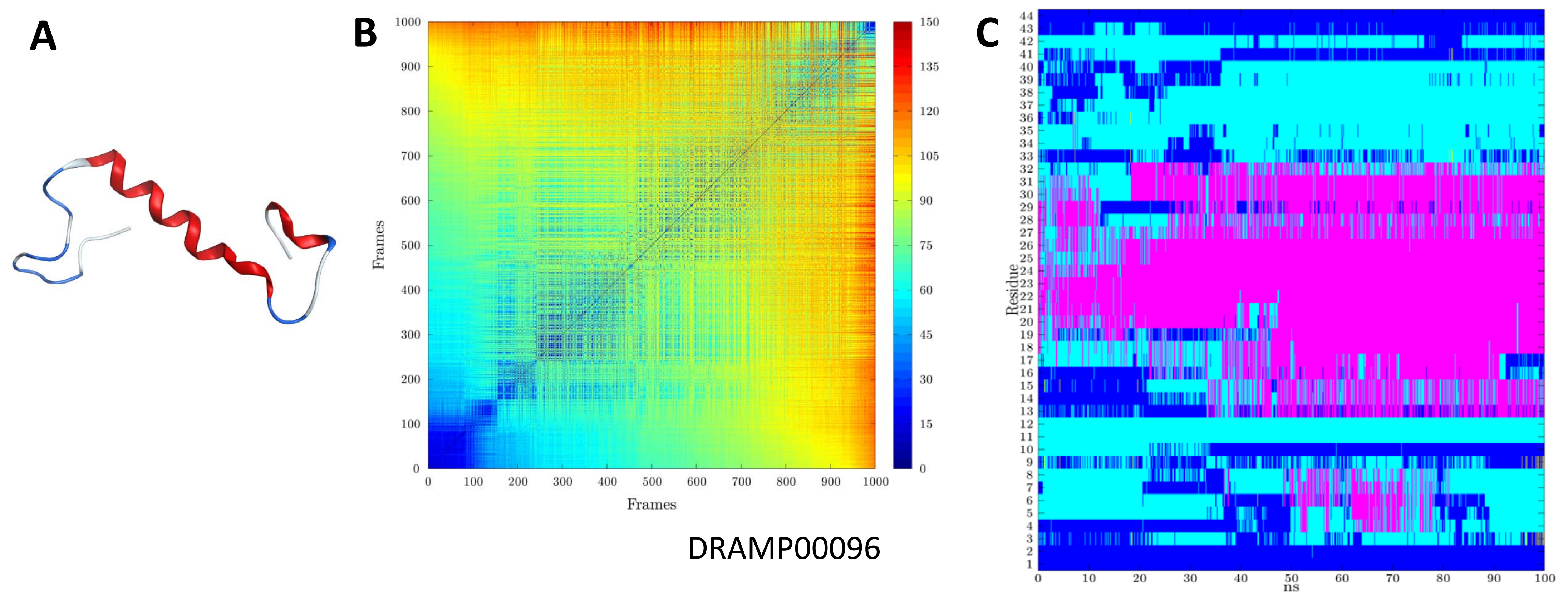

A
B
C
Frames
Frames
Residue
ns
DRAMP00096

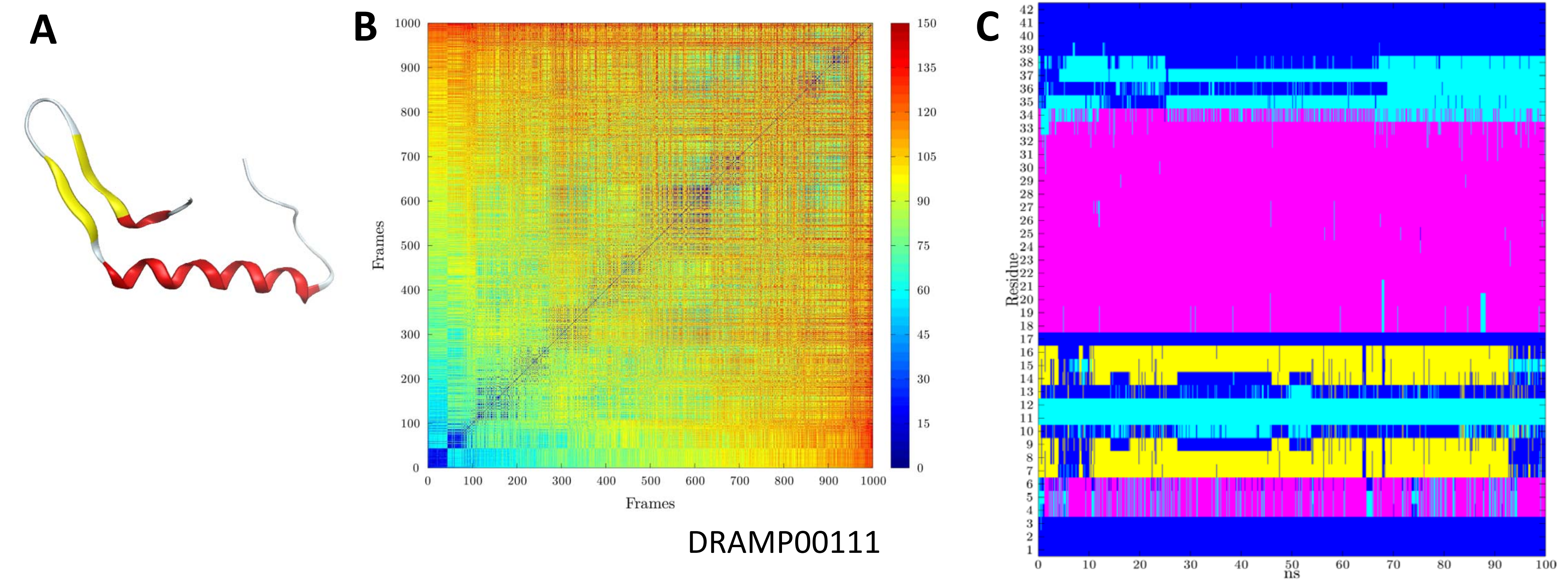

A
B
C
Frames
Frames
Residue
ns
DRAMP00111

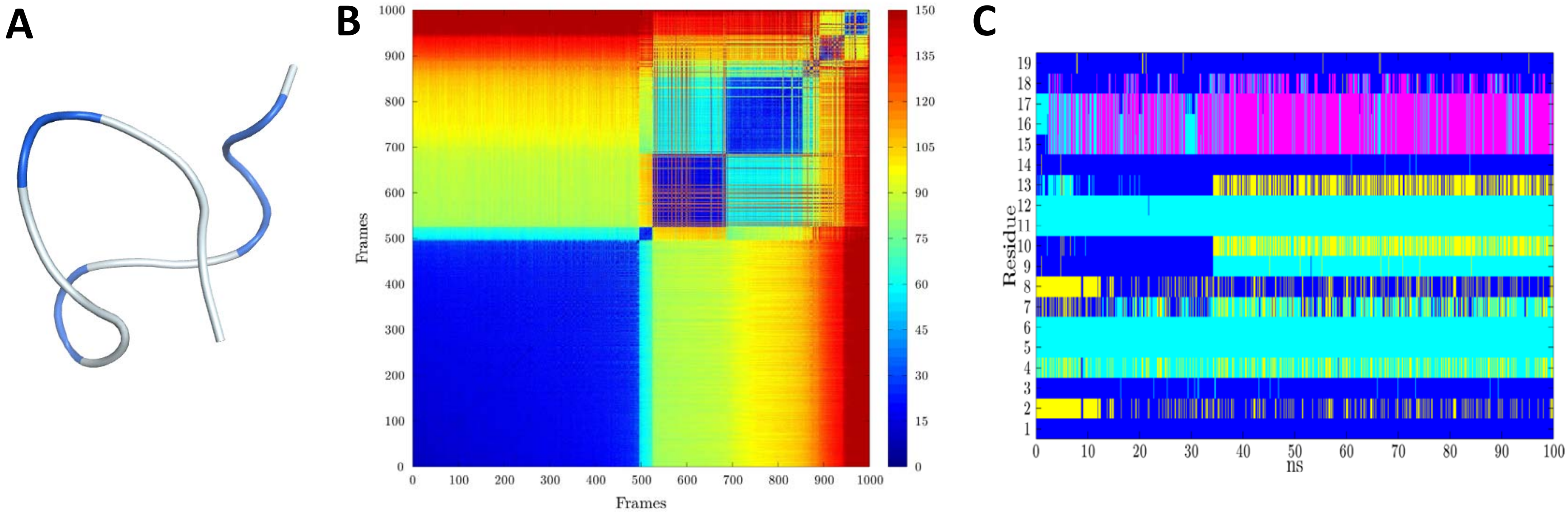

DRAMP18306

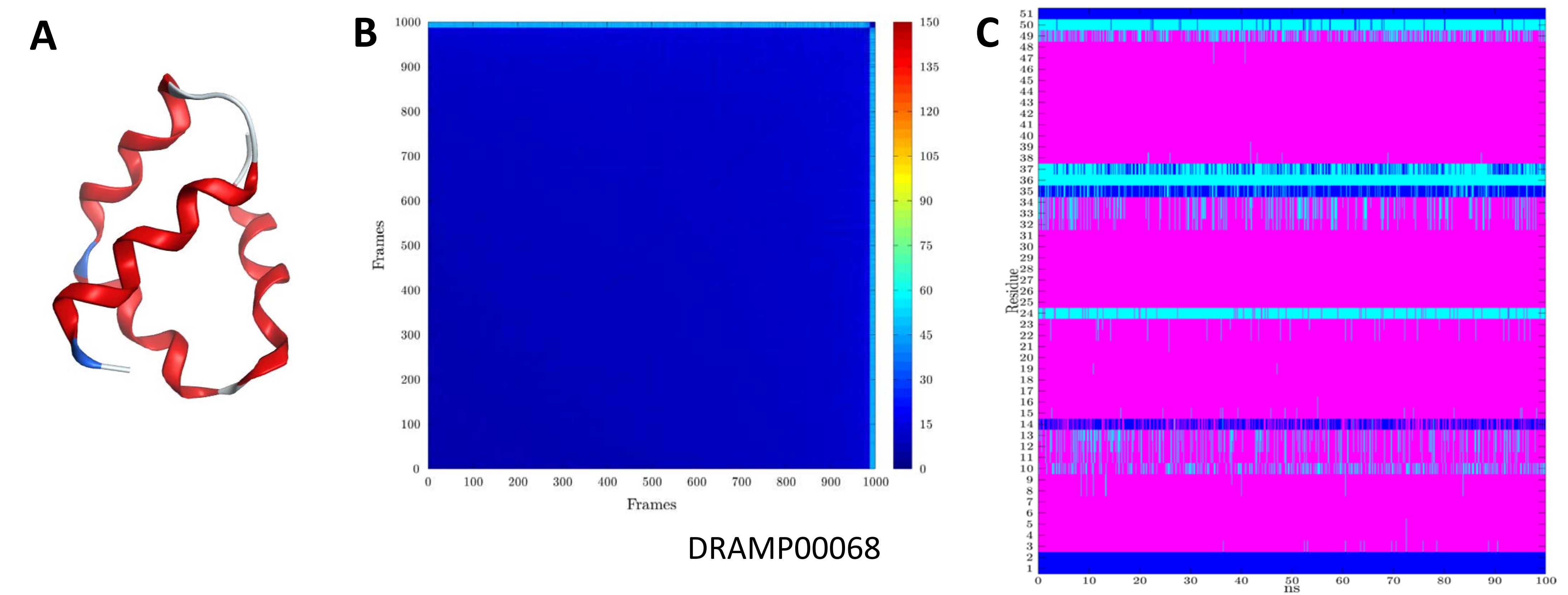
A
B
C
Frames
Frames
Residue
ns
DRAMP00068

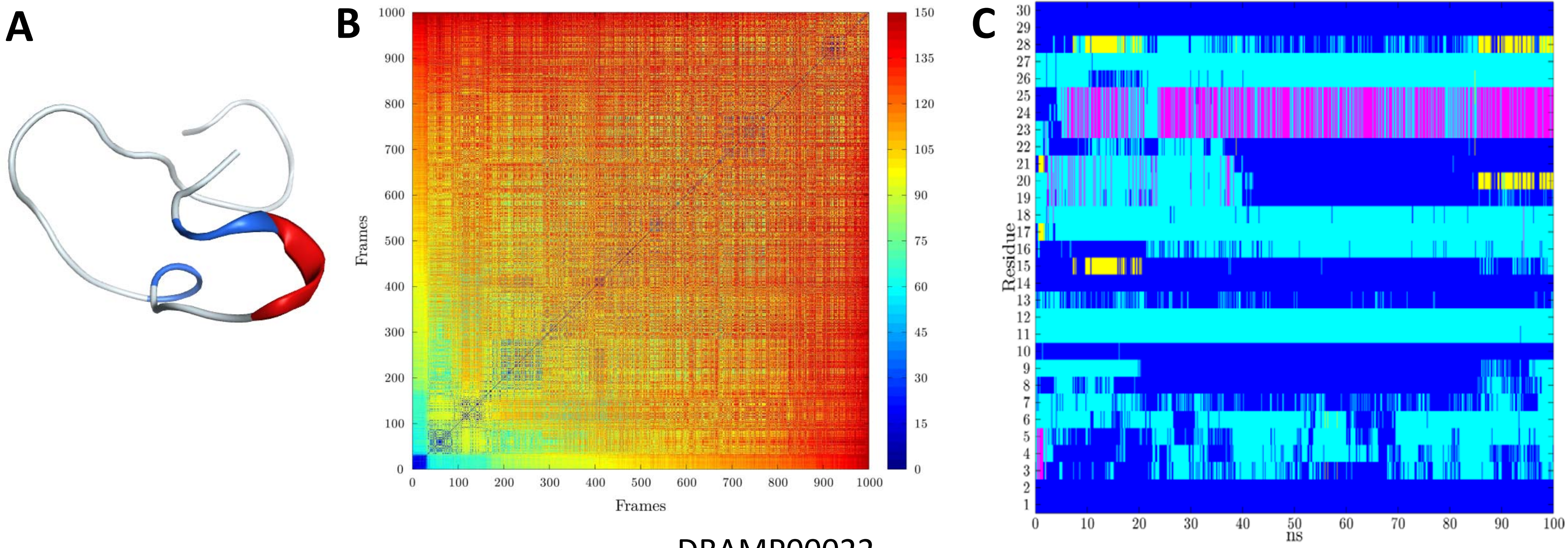

DRAMP00022

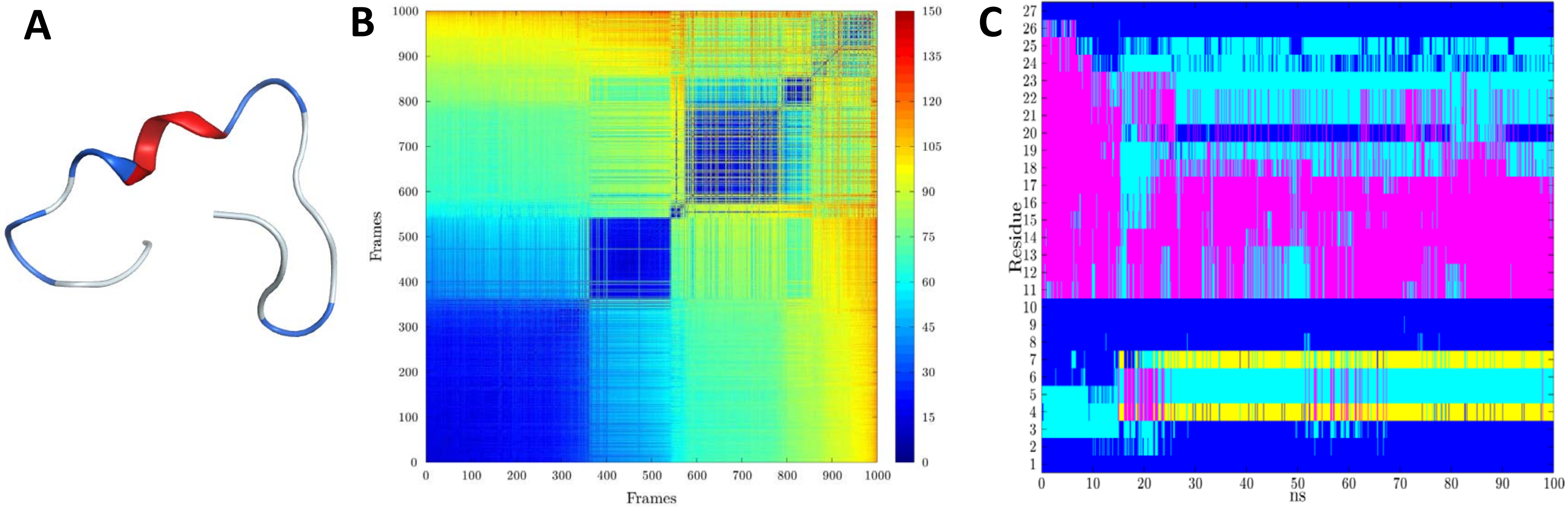

DRAMP00052

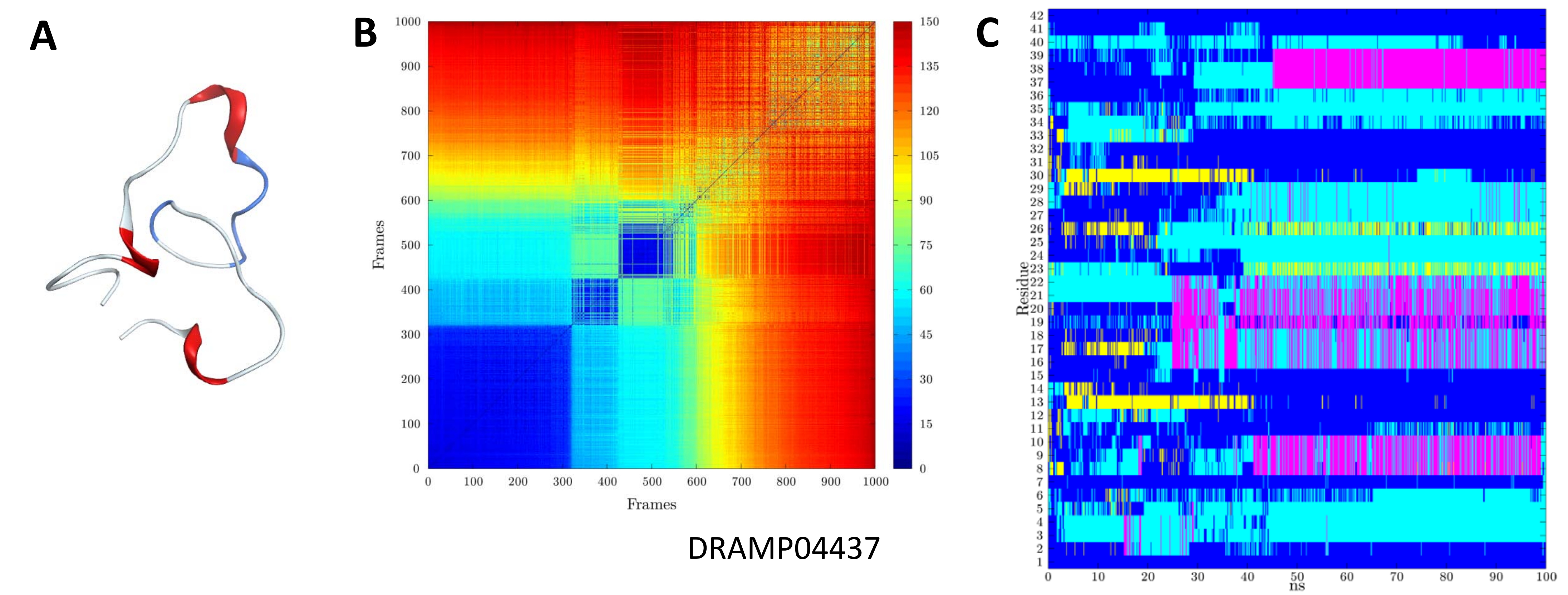
A
B
C
Frames
Frames
0
100
200
300
400
500
600
700
800
900
1000
150
135
120
105
90
75
60
45
30
15
0
DRAMP04437
Residue
ns
0
10
20
30
40
50
60
70
80
90
100

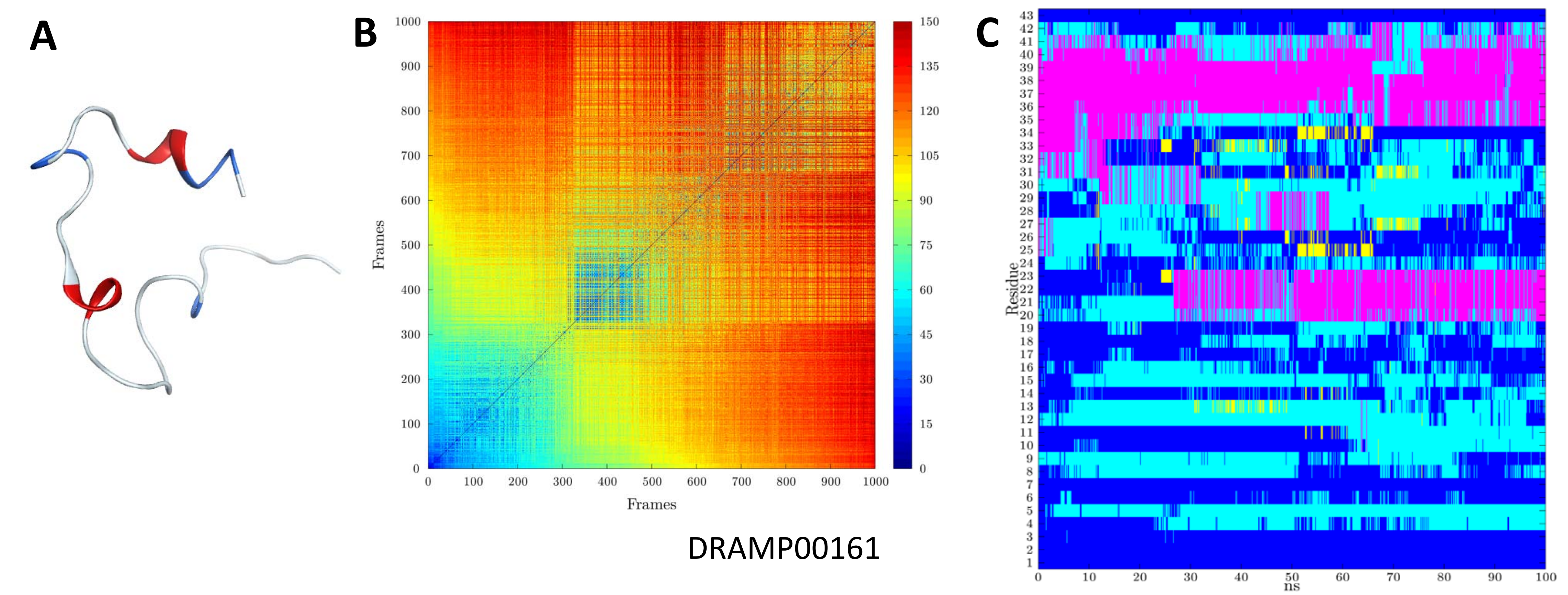
A
B
C
Frames
Frames
Residue
ns
DRAMP00161

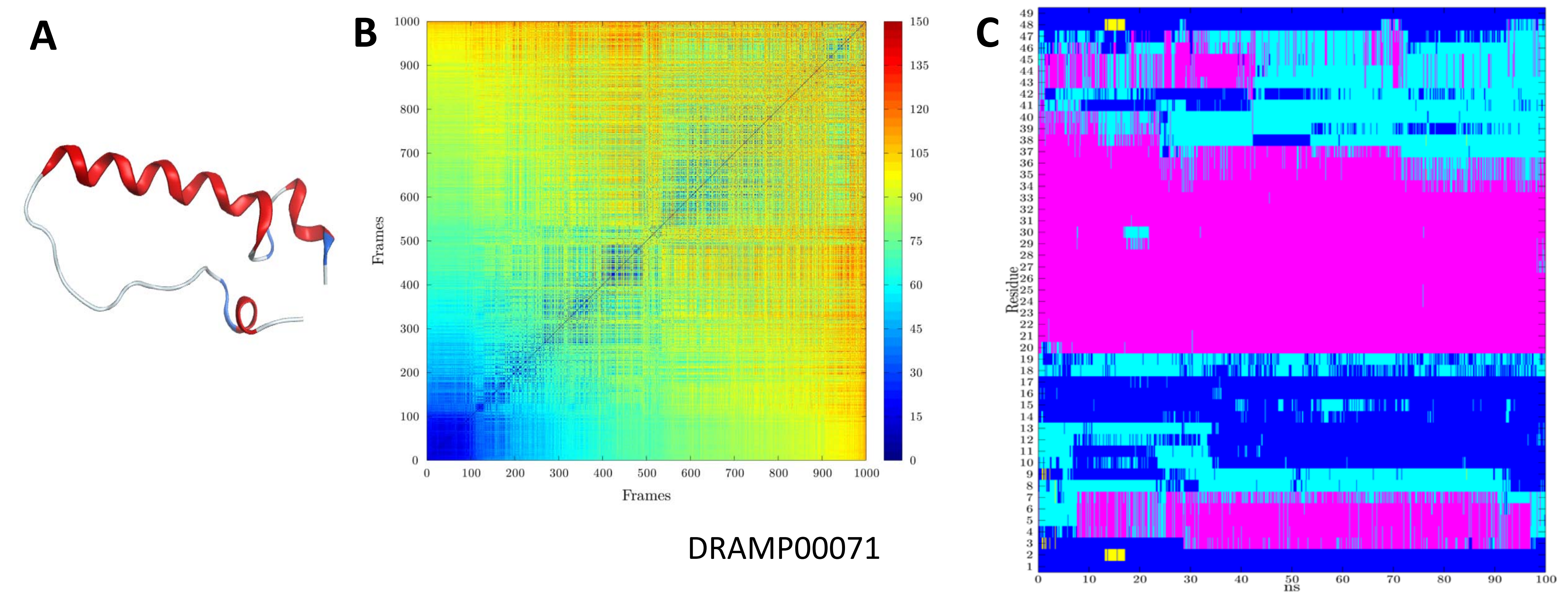
A
B
C
DRAMP00071
Frames
Frames
Residue
ns

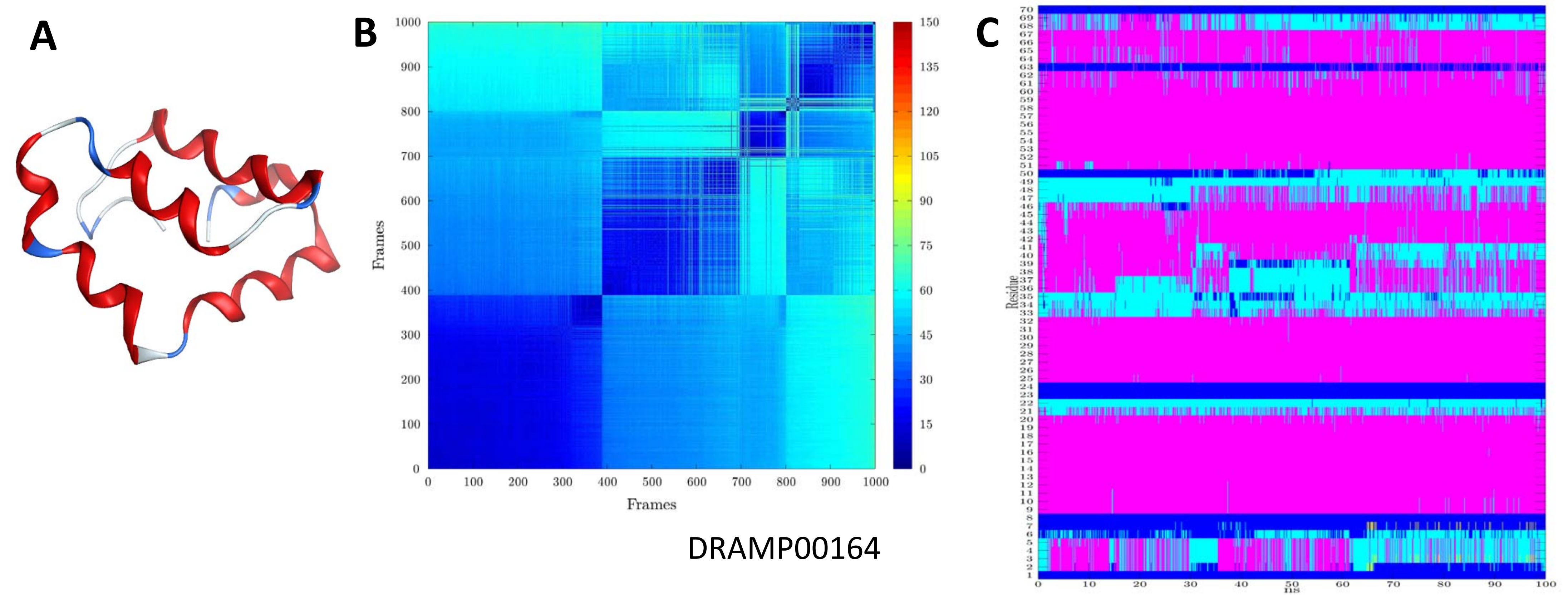

A
B
C
Frames
Frames
DRAMP00164
Residue
ns

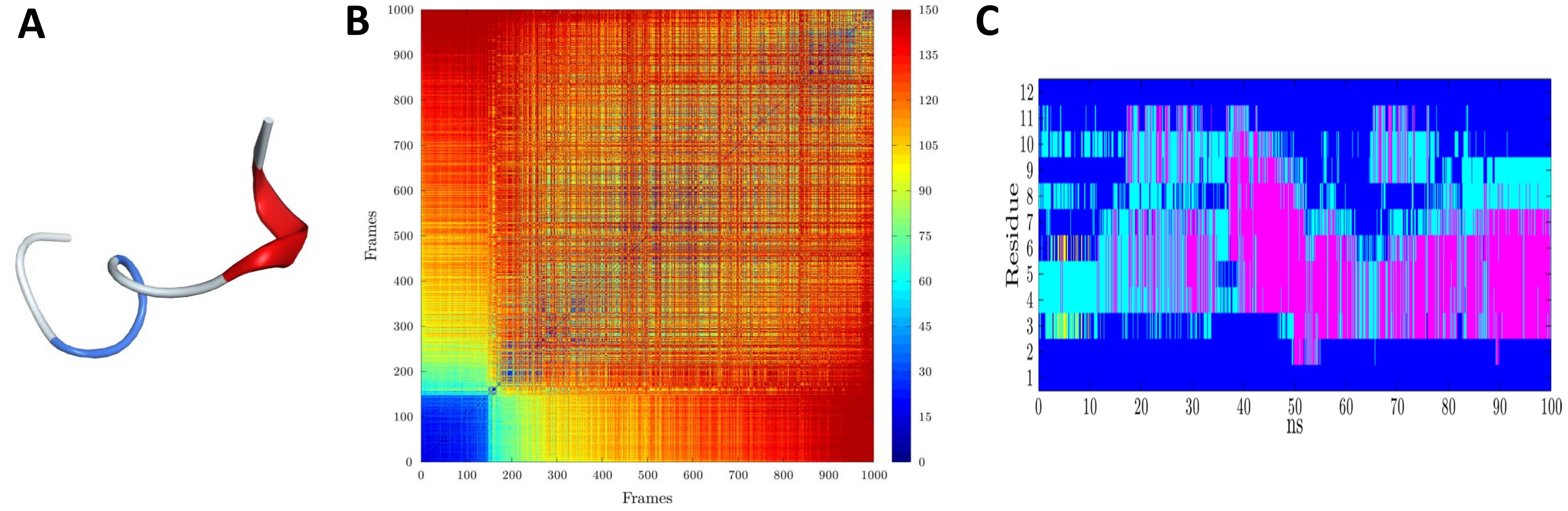
A
B
C
Frames
Frames
0
100
200
300
400
500
600
700
800
900
1000
0
15
30
45
60
75
90
105
120
135
150
Residue
ns
1
2
3
4
5
6
7
8
9
10
11
12
0
10
20
30
40
50
60
70
80
90
100

DRAMP02470

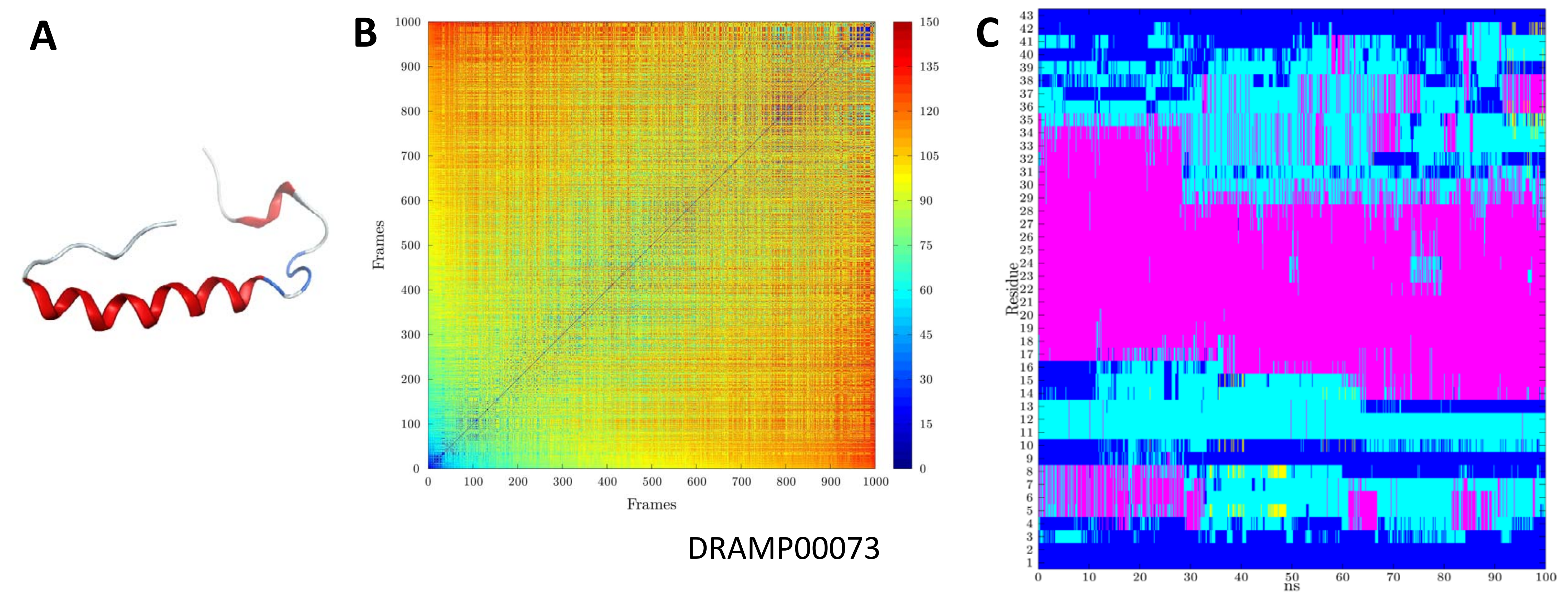
A
B
C
Frames
Frames
DRAMP00073
Residue
ns
